# Transcriptional and post-transcriptional regulation of CARMN and its anti-tumor function in cervical cancer through autophagic flux blockade and MAPK cascade inhibition

**DOI:** 10.1186/s13046-024-03229-y

**Published:** 2024-11-19

**Authors:** Xing Zhang, Wenjing Yan, Hua Jin, Bingjia Yu, Hao Zhang, Bo Ding, Xue Chen, Yan Zhang, Qianqian Xia, Dan Meng, Jing Hu, Haohan Liu, Yamei Nie, Fengying Liu, Yun Zheng, Yiran Lu, Juan Wang, Mulong Du, Meilin Wang, Evan Yi-Wen Yu, Xiuting Li, Shizhi Wang

**Affiliations:** 1 Key Laboratory of Environmental Medicine Engineering, School of Public Health, Ministry of Education, Southeast University, No. 87 Dingjiaqiao, Gulou District, Nanjing, China; 2 Clinical Laboratory, Affiliated Tumor Hospital of Nantong University (Nantong Tumor Hospital), Nantong, China; 3 School of Health Management and Basic Science, Jiangsu Health Vocational College, Nanjing, China; 4 School of Biological Sciences & Medical Engineering, Southeast University, Nanjing, China; 5 Department of Gynecology and Obstetrics, School of Medicine, Zhongda Hospital, Southeast University, Nanjing, China; 6 School of Medicine, Shihezi University, Xinjiang, China; 7 Department of Environmental Genomics, Jiangsu Key Laboratory of Cancer Biomarkers, Prevention and Treatment, Collaborative Innovation Center for Cancer Personalized Medicine, Nanjing Medical University, Nanjing, China

**Keywords:** LncRNA, Cervical cancer, CARMN, Polymorphism, RNA stability

## Abstract

**Background:**

LncRNAs play essential roles in multiple tumors. However, research on genome-wide lncRNA alterations and their functions in cervical cancer (CC) is limited. This study aims to explore key lncRNAs in CC progression and uncover the molecular mechanisms involved in the development of CC.

**Methods:**

In this study, we analyzed 30 tissues from CC, cervical intraepithelial neoplasia (CIN), and normal (NOR) using transcriptome sequencing and weighted gene co-expression network analysis to establish gene modules related to the NOR-CIN-CC transition. Machine learning diagnostic models were employed to investigate the role of lncRNAs in this transition. Molecular biological experiments were conducted to elucidate the potential mechanisms of CARMN in CC, with a particular focus on its transcriptional and post-transcriptional regulation of abnormal expression in CC.

**Results:**

CARMN was identified as a hub gene in two modules significantly associated with the NOR-CIN-CC transition. Analysis using ten machine learning models confirmed its critical role in this progression. The results of RNA-seq, qPCR and RNAScope performed in another cohort of 83 cervical tissues all showed that CARMN was significantly downregulated in CC. CARMN significantly enhanced the interaction between Keap1 and Nrf2, leading to increased ROS levels. The elevated ROS levels suppressed the Akt/mTOR signaling pathway, leading to autophagy arrest via autophagic flux blockade. Additionally, CARMN interacted with TFAP2α to repress MAPK13 transcription, further inhibiting the MAPK cascade. A promoter SNP (rs12517403) was found to increase CC risk (OR = 1.34, 95% CI = 1.11–1.61) and reduce CARMN expression by decreasing SP1 binding. Furthermore, the RNA binding proteins that could modulate CARMN RNA stability were also determined using RNA-pulldown assay. The results demonstrated that YBX1, a component of the coding region instability determinant (CRD)-mediated mRNA stabilization complex, promoted CARMN RNA stability. DHX9, another component of complex, acted as a scaffold to bridge YBX1 and CARMN.

**Conclusions:**

CARMN exerts an anti-cancer effect in CC progression by inhibiting the Akt-mTOR and MAPK signaling pathways. rs12517403 and the YBX1/DHX9 complex are key mechanisms influencing its transcription and stability in CC cells. CARMN represents a promising biomarker for CC diagnosis and therapeutic target.

**Supplementary Information:**

The online version contains supplementary material available at 10.1186/s13046-024-03229-y.

## Introduction

Cervical cancer (CC) is one of the most common gynecological cancers, particularly in less developed countries, and is responsible for approximately 340,000 deaths per year [1]. Although the incidence and mortality rates of CC have fallen dramatically in developed countries due to the implementation of human papillomavirus (HPV) vaccination and screening programs in the past decades, the mortality rate in less developed countries remains high, with approximately 85% of patients progressing to death [2, 3]. The limited availability and coverage of vaccines still make HPV a remarkable public health issue [4, 5], and prophylactic vaccine is ineffective against pre-existing HPV infections [6]. Therefore, there is an urgent need to investigate factors in addition to persistent HPV infection [7] to curb the incidence of CC. Growing evidence suggests that individual epigenetic susceptibility, particularly the regulation of long non-coding RNAs (lncRNAs), plays a fundamental role in the occurrence and metastasis of CC [8–10]. LncRNAs has been reported to be involved in a variety of biological processes, such as inhibiting DNA synthesis, transcription and post-transcriptional regulation, protein translation modulation, and ultimately affecting the development of cancers [11]. Although several lncRNAs and their underlying mechanisms in CC have been previously reported [12], research on genome-wide alterations in lncRNA expression and their biological function remains limited.

Several studies have used high-throughput methods, such as RNA-seq or microarrays, coupled with scale-free network analyses, to investigate the mechanisms of lncRNAs underlying the development and progression of various cancers including CC [13–15]. In particular, weighted gene coexpression network analysis (WGCNA) has emerged as a powerful tool for revealing molecular gene networks underlying biological functions [16]. WGCNA enables the identification of modules of gene networks [17] that are involved in biological functions of interest.

In the present study, RNA-seq was used to obtain the transcriptome profile of 30 tissues from normal (NOR), cervical intraepithelial neoplasia (CIN), which is cervical precancerous lesion, and CC. WGCNA was then employed to identify the essential modules related to the NOR-CIN-CC transition. Using this approach, the lncRNA CARMN (cardiac mesoderm enhancer-associated noncoding RNA, also known as MIR143HG), a host gene of the well-known tumor suppressor miR-143/145 [18], was identified as a key modulator in the two modules associated with the NOR-CIN-CC transition.

The dysregulation of CARMN has been reported to participate in development, progression and chemosensitivity of malignancies [19–21], however, the underlying mechanism in relation to CC remains poorly understood. To address this gap, we conducted an analysis of cervical tissue sequencing data, identifying CARMN as a key lncRNA involved in the carcinogenic process of CC. Through animal and cellular experiments, we further elucidated the potential role of CARMN in CC. Furthermore, we investigated the molecular mechanisms underlying its downregulation in CC tissues, providing new insights into the progression of CC and potential precision treatment options.

## Materials and methods

### Study subjects

A total of 30 cervical tissues consisting of 10 CC tissues, 10 CIN tissues, and 10 normal cervical tissues, were utilized for RNA-seq analysis. Additional 83 tissues including 23 cervical squamous carcinomas, 20 adenocarcinomas, and 40 normal tissues, were enrolled to validate the expression of CARMN. The specimens were obtained from patients undergoing surgery at the Southeast University Affiliated Zhongda Hospital between 2018 and 2019 with informed consent and agreement. All samples were used in compliance with the institution’s ethical regulations. The patients’ information was presented in Table S1. For details, please see the methods section of the supplementary data.

The details of subjects for association analyses have been described elsewhere [22]. Briefly, a total of 571 CC cases and 657 controls were collected in the first phase as primary study, while additional subjects were enrolled in the second phase as secondary study, through which the included participants were 2293 consisting of 954 cases and 1339 controls. The demographic and clinical characteristics of all study subjects were presented in Table S2.

### RNA-seq analysis

The total RNA was extracted from frozen tissue specimens or cells using TRIzol reagent (Thermo, USA), and subsequently subjected to high-throughput sequencing on Illumina HiSeqTM 4000 by a contract service at the Gene Denovo Biotechnology Co. (Guangzhou, China). The specific procedures and data analysis pipeline were described in the supplementary data.

### RNA reverse transcription and real-time PCR (qPCR)

RNA extraction was performed using TRIzol reagent (Thermo, USA), followed by RNA quantification. The cDNA was then synthesized using a reverse transcription kit (Vazyme, China), which was utilized for subsequent qPCR analysis. The SYBR^®^ Green Realtime PCR Master Mix-Plus Kit (Toyobo, Japan) was used to quantify the expression of mRNA and miRNA with GAPDH and U6 as internal control, respectively. All primers involved in this study were listed in Table S3.

### WGCNA

A co-expression network for mRNAs and lncRNAs sourced from RNA-seq in 30 cervical tissues mentioned above was constructed using R package WGCNA [17]. The transcription factor (TF) prediction analysis was performed to identify TFs that might bind to the target RNAs using R package *TFBSTools*, of which the results were used to build a TF network and visualized using Cytoscape (version 3.9.1) (for details, please see the methods section of the supplementary data).

### Machine learning

CC-related datasets (GSE63514, GSE6791, GSE27678, and GSE75132) were obtained from the Gene Expression Omnibus (GEO) database (https://www.ncbi.nlm.nih.gov/gds/). Subsequently, machine learning models were constructed, with 75% of the samples randomly selected for model training and the remaining samples used for model validation. In this study, ten machine learning algorithms, labeled Decision Tree (DT), Random Forest (RF), Extreme Gradient Boosting (XGBOOST), Elastic Net Regression (ENR), Support Vector Machine (SVM), Multi-Layer Perceptron (MLP), Light Gradient Boosting Machine (Lightgbm), k-Nearest Neighbors (KNN), Logistic Regression (LR), and Stacking Ensemble (Stacking), were employed. Model performance was evaluated using receiver operating characteristic (ROC) and precision-recall (PR) curves and their respective areas under the curve (AUC) for both the training and validation sets. For interpreting variable importance within the models, descriptive machine learning explanations (DELAX) and shapley additive explanations (SHAP) were utilized to display the importance of variables for each model. For details, please see the methods section of the supplementary data.

### Cell culture, plasmid and lentiviral vectors

The details of cell lines and reagents used for this study were described in the supplementary data. Chemicals used for cell treatment were listed in Table S4. The plasmid and lentiviral vectors (Genechem (Shanghai, China); Table S5) were employed for RNA interference or gene overexpression. For stable transduction, the cell culture was subjected to antibiotic selection (for details, see the methods section of the supplementary data).

### Xenograft model

HeLa-CARMN and HeLa-NC cells (1 × 10^7^ per mouse) were injected subcutaneously to the nude mice to establish xenograft model. Each group had six mice. Xenograft growth curves were drawn during 30 days after inoculation (for details, see the methods section of the supplementary data).

### Luciferase assay

The reporter plasmids used for analyzing the transcriptional activity of the target gene promoter were synthesized on the basis of the pGL3-Basic vector (Promega, USA). The plasmids with or without siRNAs were transiently transfected into cells using Lipofectamine 2000 (Invitrogen, USA) with pRL-SV40 (Promega, USA), which contained the Renilla luciferase gene, as internal standard. After transfection at 48 h, cells were collected and Firefly-Renilla luciferase activities were measured using the Dual-Luciferase^®^ Reporter Assay System (Promega, USA). The detailed information of the constructs was shown in Table S5.

### CCK-8 assay

Cells were seeded in the 96-well plate (1 × 10^4^/well). Cell culture was continued for 24, 48, 72 and 96 h (unless otherwise specified), and followed by incubation with CCK-8 reagent (Beyotime, China) for another 1 h at 37 °C. The absorbance was then tested at a wavelength of 450 nm.

### Plate colony formation assay

Cells were seeded in 6-well plates and cultured in a complete medium for 14 days. Cells were then fixed with formaldehyde and stained with 0.1% crystal violet. Colonies were photographed and counted using ImageJ software.

### Transwell assay

Transwell assay was performed using the 12-well Transwell chambers (Corning Costar, USA) with a pore size of 8 μm, and described with details in the supplementary data.

### Flow cytometry

Cell proliferation and apoptosis were detected with propidium iodide (PI) and Annexin V-PE/7-AAD Apoptosis Detection Kit (Vazyme, China), respectively, using a flow cytometric method. The experimental details were described in supplementary materials.

### RNAScope

The abundance and location of lncRNA CARMN in cervical tissue can be detected by RNAScope experiment (#493421, ACD, USA). The experimental details were described in supplementary materials.

### Chromatin immunoprecipitation (ChIP)

ChIP was performed as previously reported [23] with a slight modification using antibody against SP1 (#9389, CST, USA), TFAP2α (#13019-3-AP, Proteintech, USA) or control IgG.

### RNA binding protein (RBP) immunoprecipitation assay (RIP)

The interaction between RBPs and CARMN was detected by RIP, which was performed with RIP™ RNA-Binding Protein Immunoprecipitation Kit (Merck Millipore, USA) as the manufacturer’s instructions and described with details in the supplementary data. The RIP antibodies against TFAP2α and tag protein HA were listed in Table S4. The abundance of CARMN bound to TFAP2α was quantified via qPCR.

### RNA-pulldown

RNA-pulldown was conducted using Pierce Magnetic RNA-Protein Pull-Down Kit (Thermo Fisher, USA) according to the manufacturer’s instructions and described with details in the supplementary data.

### Hematoxylin-Eosin (HE) staining

The paraffin embedded tissue was cut into thin slices, dewaxed successively with xylene, ethanol and distilled water, and then dyed in hematoxylin dye and eosin dye (C0105M, Beyotime, China). Then, it was washed and dehydrated with gradient ethanol and treated with xylene in turn, and finally sealed for microscopic observation.

### Immunohistochemistry (IHC)

IHC for Ki67, p-Akt, p-mTOR, P62, and Nrf2 was carried out as described in our previous publication [24]. The detailed procedures were described in the supplementary data.

### Western blot (WB)

WB was performed as described previously [23] using antibodies listed in Table S4. The levels of GAPDH and Lamin B1 were used as reference for cytoplasmic and nuclear proteins, respectively.

### Autophagic flux analysis

Autophagic flux was monitored by mRFP-GFP-LC3 lentivirus (Shanghai Genechem Co., Ltd., China), as described elsewhere [25].

### Nuclear-cytoplasmic fractionation assay

Cells in the logarithmic growth phase were seeded into culture dishes and harvested at approximately 80% confluence for total protein extraction and nuclear-cytoplasmic fractionation. A commercial kit (#78835, Thermo Fisher, USA) was used according to the manufacturer’s instructions. Briefly, cells were collected, lysed with cold buffer to extract the cytoplasmic fraction. The remaining pellet was subjected to strong lysis and ultrasonic disruption to isolate nuclear components.

### Genotyping

Genomic DNA was extracted using TIANamp Blood DNA Kit (TIANGEN Biotech, Beijing, China) from peripheral blood lymphocytes of included subjects. Genotypes for each SNP were analyzed using SNaPshot as previously described [26]. Briefly, the target fragments were amplified and extended using PCR. The primer sequences were listed in Table S13. The resulting extension products were then mixed with loading buffer followed by sequencing. The candidate promoter SNPs were further genotyped using TaqMan allelic discrimination method.

### Statistical analysis

For descriptive statistics, normally distributed continuous variables were presented as mean ± standard deviation, and non-normally distributed continuous variables were presented as median (interquartile range). Categorical variables were represented as frequency (percentage, %). The difference of categorical and continuous variables between groups was compared using chi-square test and ANOVA test, respectively, unless otherwise indicated. Unconditional logistic regression model was used to estimate the odds ratio (OR) and 95% confidence intervals (CI) for evaluating the association between genotypes and CC risk. Differences were considered statistically significant at *P* < 0.05. To account for multiple testing, a false discovery rate correction was applied. All statistical analyses were performed by R software (version 4.2.3 and 4.4.1). The analysis results were visualized by GraphPad Prism 9.0. More details of materials and methods are described in Supplementary Materials and Methods.

## Results

### Transcriptome-based WGCNA combined with machine learning identified the hub lncRNA CARMN as related to the NOR-CIN-CC transition

**Fig. 1:**
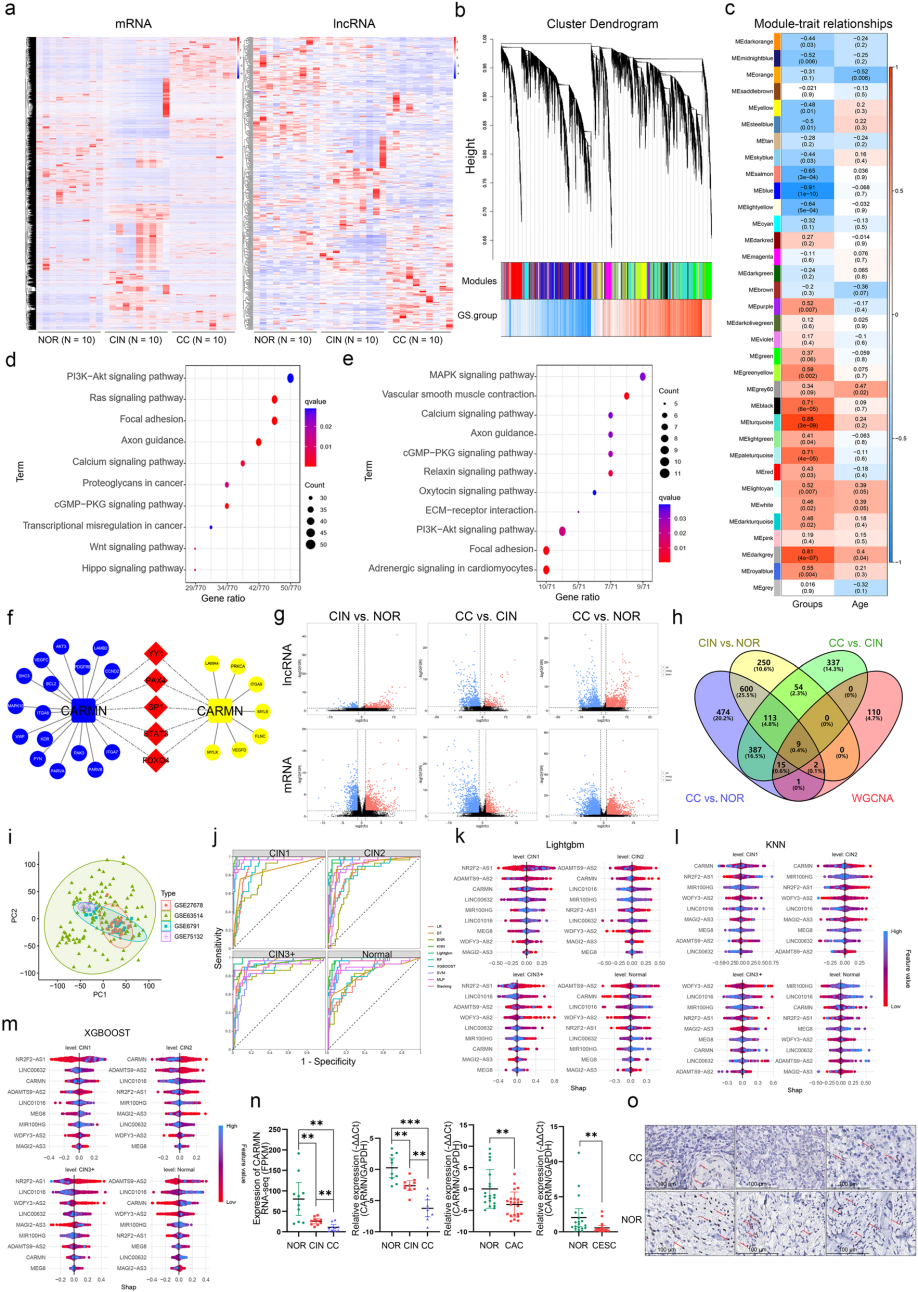
Transcriptome-based WGCNA analysis identified hub lncRNA CARMN related to the NOR-CIN-CC transition. **a **Heatmaps of expression levels of mRNAs (left) and lncRNAs (right) by RNA-seq in 10 CC, 10 adjacent normal tissues (NOR), and 10 CINs. **b** A total of 34 modules obtained by WGCNA analysis with a correcting cutHeight = 0.25 by the Dynamic Tree-Cut algorithm. **c** Associations of modules with groups and ages. **d**-**e** KEGG pathway analysis of hub mRNAs in the Blue (**d**) and Lightyellow (**e**) Module. **f** CARMN-TF-mRNA network in the two modules by WGCNA. Blue color indicates the molecule belongs to the blue module, while yellow color belongs to the lightyellow module. The circles represent mRNAs, the squares represent lncRNAs, and the red oblique square represent transcription factors. **g** The RNA-seq group comparison showed that red dots represented upregulated genes, blue dots represented downregulated genes, and black dots indicated no significant difference between the two groups. **h** Nine key lncRNAs involved in the cervical carcinogenesis process were identified by intersecting differentially expressed lncRNAs with those in key modules from WGCNA. **i** The PCA results showed that the sample characteristics were highly consistent across datasets after batch correction. **j** The ROC curves for each group were presented using ten different machine learning algorithms. **k**-**m** Lightgbm (**k**), KNN (**l**), and XGBOOST (**m**) demonstrated the best performance in predicting sample groups, with variable importance explained based on SHAP values. (**n**) Validation of CARMN expression in the original 30 and additional 83 cervical tissues. From left to right: RNA-seq data, qPCR results of original 30 cervical tissues, cervical adenocarcinomas (CAC, 20 tumors vs. 20 NOR), and additional tissues of cervical squamous cell carcinomas (CESC, 23 tumors vs. 20 NOR). **o** RNAScope results showed the abundance of CARMN in tumor and normal tissues. ns, *P* ≥ 0.05; *, *P* < 0.05; **, *P* < 0.01; ***, *P* < 0.001

To access the role of lncRNAs and mRNAs in the NOR-CIN-CC transition, the transcriptome expression profiles of tissues (*n* = 10 in each group) were determined using RNA-seq (Figs. 1a and S1a-c). Table S6 summarized the top 20 dysregulated lncRNAs and mRNAs in each comparison.

Given that co-expressed genes are biologically related, the study used WGCNA to group highly correlated genes. The integrated networks of mRNAs and their correlated lncRNAs were constructed in RNAs with a difference at fold-change ≥2 (Fig. S1d-e). A total of 34 modules were identified (Fig. 1b), among which 20 modules were markedly related to the NOR-CIN-CC transition, i.e., 8 negative and 12 positive correlations, while only 3 modules were found in relation to age (Fig. 1c). The Blue module had the most powerful correlation with CC progression (*r* = -0.91, *P* = 1 × 10^−10^). Furthermore, the Blue and Lightyellow modules were clustered together through eigengene adjacency analysis (Fig. S1f-h). The KEGG and GO enrichment analysis of co-expressed mRNAs illustrated many enriched pathways, including PI3K-Akt, Focal adhesion signaling pathways, MAPK, and Ras signaling pathways (Figs. 1d and e and S1i-j), which are critical in CC development and progression [27]. Since the Lightyellow module was also significantly related to CC progression (*r* = -0.64, *P* = 5 × 10^−4^), both the Blue and Lightyellow modules were selected as candidate modules for the following analyses. MAPK10, AKT3, FYN, CCND2, PDGFRB, KDR, SHC3, MIR99AHG, MEG8 and CARMN, were identified as the hub genes (edges > 20) in the Blue module, while ITGA9, LAMA4, MYLK, FLNC, MYL9 and CARMN were identified as the hub genes in the Lightyellow module (Fig. S1k). Intriguingly, two CARMN transcripts were respectively located in the Blue and Lightyellow modules, suggesting that CARMN plays a vital role in the development and progression of cervical carcinogenesis.

The study proposed a hypothesis that there might be some TFs linking the two modules through regulating the common essential hub genes like CARMN. Using a total of 34 predicted TFs, a mRNA/lncRNA-TF interaction network across the two modules was constructed. Among these TFs, YY1, STAT3, FOXO4, PAX4 and SP1 were noticeable due to their abundant linking nodes (Fig. S1l). The CARMN-TF-mRNA network shown in Fig. 1f was established based on highly correlated, differentiated expressed mRNAs and potentially regulating TFs (including YY1, STAT3, and SP1) in the two modules.

Based on the differentially expressed lncRNAs identified between the groups, we intersected the genes consistently differentially expressed across the NOR-CIN-CC transition (Fig. 1g) with key lncRNAs identified by WGCNA. This intersection yielded nine lncRNAs (LINC01016, NR2F2-AS1, CARMN, MAGI2-AS3, MIR100HG, WDFY3-AS2, LINC00632, ADAMTS9-AS2, and MEG8) that were highly associated with cervical carcinogenesis and were consistently downregulated, which were selected as candidate lncRNAs for further analysis (Fig. 1h).

To further investigate the relative contributions of the nine candidate lncRNAs in the progression of CC, we employed machine learning to assess the relative importance of these lncRNAs across different CC groups. The best-fitting model was determined using ROC and PR curves, with the goal of identifying the most critical lncRNAs associated with CC clinical staging. We downloaded four cervical cancer-related datasets from the GEO database, including GSE63514, GSE6791, GSE27678, and GSE75132, all of which were based on the GPL570 platform and possessed a relative larger sample size than 10. Finally, a total of 198 samples were included for the new dataset. After standardization and batch effect correction, the datasets were merged into a new dataset for further analysis. PCA results demonstrated that the batch effects between datasets were significantly removed after processing (Figs. 1i and S2). In our previous study [28], we used 9 machine learning algorithms to identify the key SUMOylation factors in the occurrence and development of CC. In the present study, in addition to the nine machine learning methods used above, we also incorporated a stacking ensemble (Stacking) algorithm to construct diagnostic models for CC, exploring the feasibility of predicting its occurrence and progression based on the nine key lncRNAs and further investigating their potential as biomarkers.

Through AUC comparison, the results showed that Lightgbm (AUC_ROC_ = 0.987, AUC_PR_ = 0.966), KNN (AUC_ROC_ = 0.968, AUC_PR_ = 0.929), and XGBOOST (AUC_ROC_ = 0.963, AUC_PR_ = 0.917) exhibited the best diagnostic performance among the ten models (Figs. 1j, S3 and Table S7). In these three models, CARMN consistently ranked among the top three in importance for the Normal, CIN1, and CIN2 groups (Fig. 1k and m), suggesting that CARMN could serve as a biomarker in the progression of CC.

To further validate the aberrant expression of CARMN in CC populations, we conducted a pan-cancer analysis including CC using the online platform GEPIA (Gene Expression Profiling Interactive Analysis; http://gepia.cancer-pku.cn/). The expression of CARMN from The Cancer Genome Atlas (TCGA) and Genotype-Tissue Expression (GTEx) showed a significant downregulation in 16 out of 31 tumor type cohorts compared with corresponding normal tissues, especially for cervical squamous carcinoma (CESC) (Fig. S4). Consistent with the RNA-seq results, qPCR analysis of the 30 original samples in this study confirmed decreased CARMN in the CIN and CC, as compared with the normal tissues (Fig. 1n). Downregulation of CARMN in CC was also validated using qPCR and RNAScope (Fig. 1n and o, respectively) in the 83 additional tissues. Taken together, the observation of consecutive decrement of CARMN during the NOR-CIN-CC transition indicated a strong association between CARMN and cervical carcinogenesis.

### CARMN acted as a tumor suppressor in CC

**Fig. 2:**
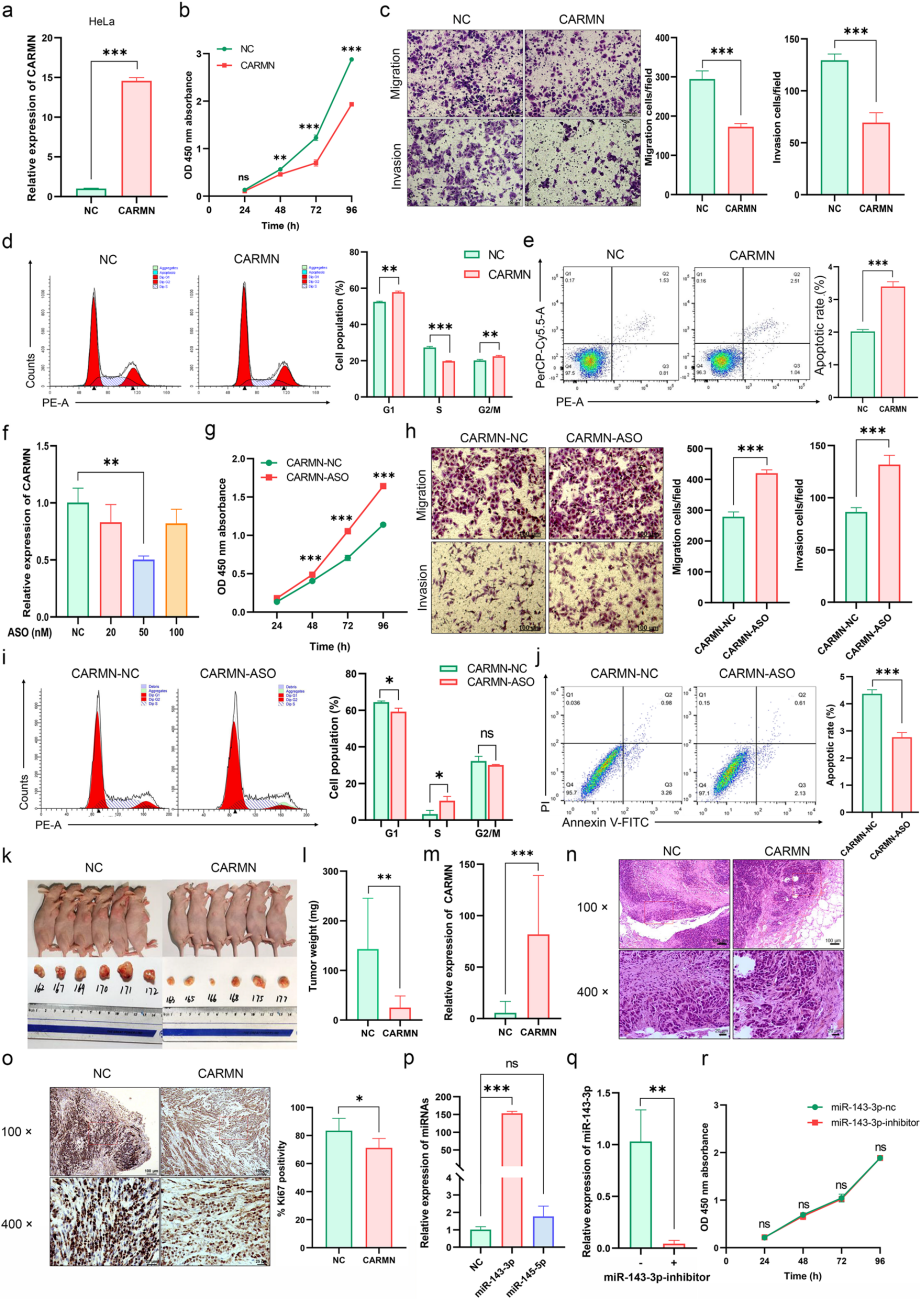
CARMN played an anti-tumor role in CC cells independent of miR-143/145. **a** The expression levels of CARMN in the CARMN stably high-expressed HeLa cell. **b** The effect of CARMN overexpression on the proliferation of HeLa cells by CCK-8. **c** The effect of CARMN overexpression on the migration and invasion abilities of HeLa cells by transwell assays. **d** The effect of CARMN overexpression on the cell cycles of HeLa cells by flow cytometry. **e** The effect of CARMN overexpression on apoptosis of HeLa cells by flow cytometry. **f** The expression levels of CARMN in the CARMN-ASO HeLa cells. **g** The effect of CARMN knockdown (at a concentration of 50 nM ASO) on the proliferation of HeLa cells by CCK-8. **h** The effect of CARMN knockdown (at a concentration of 50 nM ASO) on the migration and invasion abilities of HeLa cells by transwell assays. **i** The effect of CARMN knockdown (at a concentration of 50 nM ASO) on the cell cycles of HeLa cells by flow cytometry. **j** The effect of CARMN knockdown (at a concentration of 50 nM ASO) on apoptosis of HeLa cells by flow cytometry. **k** HeLa-CARMN and HeLa-NC cells were injected subcutaneously to the 12 nude mice to establish xenograft model. **l** The weight of xenografts. **m** The levels of CARMN in xenograft tissues. **n** HE staining of xenografts. **o** IHC staining of proliferative marker Ki67 in transplanted tumor tissues. **p** The expression of miR-143-3p and miR-145-5p from qPCR data. **q** The expression of miR-143-3p after miR-143-3p inhibitor treatment in HeLa cells by qPCR. **r** The effect of miR-143-3p inhibitor on cell proliferation by CCK-8 in the HeLa-CARMN cells. ns, *P* ≥ 0.05; *, *P* < 0.05; **, *P* < 0.01; ***, *P* < 0.001

So far, the role of CARMN in CC has yet to be fully elucidated. To address this issue, CARMN stably high-expressed cells were constructed using a lentiviral vector, and the higher expression of CARMN was confirmed (Figs. 2a and S5a). CARMN overexpression could decrease the ability of proliferation (Figs. 2b and S5b), migration and invasion (Figs. 2c and S5c) in CC cells. The influence of CARMN on cell cycle and apoptosis was determined by flow cytometry. The results illustrated that CARMN induced cell cycle arrest at G1/S phase (Figs. 2d, S5d) and led to reduction in expression of Cyclin D1 and Cyclin A1/A2 in HeLa and C33A cells, respectively (Fig. S5d-f). Although there are statistically significant differences, the absolute increase in apoptosis rate in CC cells overexpressing CARMN is not pronounced (less than 2%). Hence, we speculate that CARMN may not significantly promote apoptosis (Figs. 2e & S5g) in CC cells. Knockdown of CARMN in the original HeLa cells was achieved through antisense oligonucleotides (ASO) treatment, with a knockdown efficiency of about 50% (Figs. 2f and S6a). Therefore, we used 50 nM as the final concentration for the CARMN knockdown ASO in HeLa cells. In contrast to the overexpression trend, CARMN knockdown cells showed an upregulation in proliferation rate (Figs. 2g and S6b), increased ability of migration and invasion (Fig. 2h), acceleration of the S phase transition (Fig. 2i) as compared with the NC group in HeLa, but the C33A cell line displayed a different trend with a modest decrease in the S phase (Fig. S6c). Moreover, knockdown of CARMN could decrease apoptosis rate in CC cells (Figs. 2j and S6d).

To further investigate the role of CARMN in vivo, HeLa-CARMN and -NC cells were subcutaneously injected into nude mice to establish a xenograft tumor model. As shown in Figs. 2k and m and S6e, the xenografts of the CARMN group exhibited significantly smaller tumor volume and weight compared with the NC group. HE staining of transplanted tumor tissues revealed a significantly lower malignancy degree in the HeLa-CARMN group than in the HeLa-NC group (Fig. 2n). Expression of proliferation marker Ki67 (Fig. 2o) were detected in transplanted tumor tissues. The expression of Ki67 was significantly decreased in the transplanted tumor tissues highly expressing CARMN.

MiR-143/145 has reported to be co-transcribed with their host gene, CARMN [18, 29], making it essential to determine whether miR-143/145 contributes to CARMN’s tumor suppressor function in CC. Both RNA-seq and qPCR assays revealed that only miR-143-3p exhibited a significant upregulation, with over a 150-fold change in expression in HeLa-CARMN cells compared with controls (Figs. 2p and S6f). Conversely, miR-145-5p expression remained relatively unaffected, displaying a minor increase of up to 50%. miR-145-5p was excluded from further analysis. The application of a miR-143-3p inhibitor did not significantly impact CARMN expression, cell proliferation or cell cycle progression in HeLa-CARMN cells (Figs. 2q and r and S6g-h). These findings suggest that CARMN may exert its tumor suppressor role independently of miR-143/145.

### CARMN triggered autophagic flux blockade via the ROS/Akt/mTOR pathway

**Fig. 3:**
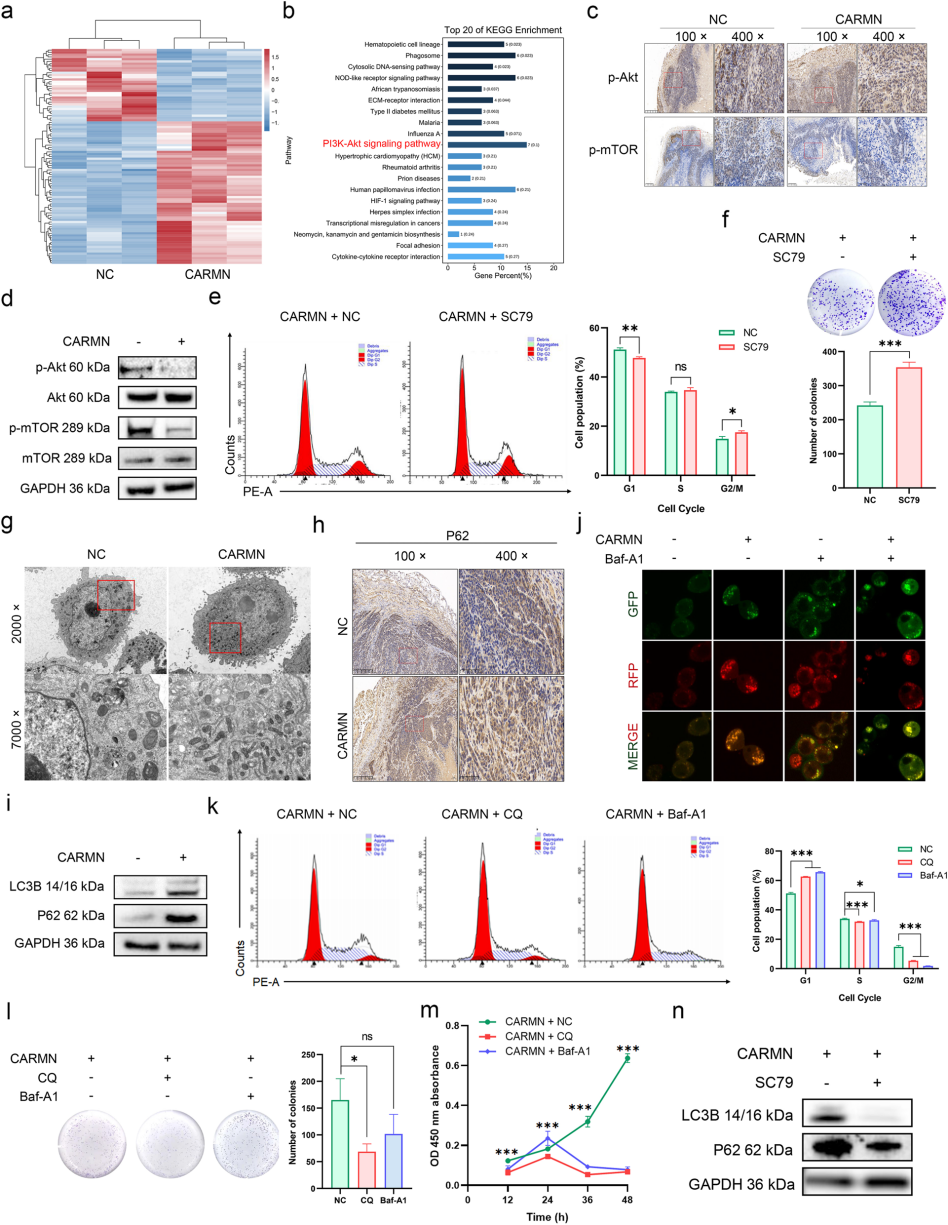
CARMN induced autophagic flux blockade by suppressing the Akt/mTOR pathway. **a** Heatmap plot showing differential expressed genes (DEGs) downstream of CARMN in HeLa cells identified by RNA-seq. **b** KEGG enrichment analysis of DEGs. **c** IHC staining of p-Akt (Thr308) and p-mTOR (Ser2448) in transplanted tumor tissues. **d** The expression of p-Akt (Thr308), p-mTOR (Ser2448) and their total proteins in HeLa cells with or without CARMN overexpression. **e** The effect of SC79 on the cell cycle in the HeLa-CARMN cells. **f** The effect of SC79 on the colony formation ability of HeLa-CARMN cells by plate colony formation assay. **g** The representative TEM images of autophagosome in the HeLa cells after CARMN overexpression. **h** IHC staining of P62 in transplanted tumor tissues. **i** The expression levels of LC3 and p62 protein in HeLa cells with or without CARMN overexpression. **j** The effect of CARMN on autophagic flux in the HeLa cells treated with or without Baf-A1 was detected by mRFP-GFP-LC3 reporter plasmid. Yellow puncta represent early autophagosomes, while red puncta late autolysosomeslate autophagosomes. **k** The cell cycles of the HeLa-CARMN cells treated with CQ or Baf-A1. **l** The colony formation ability of HeLa-CARMN cells treated with CQ or Baf-A1. **m** The proliferation of HeLa-CARMN cells treated with CQ or Baf-A1 by CCK-8. **n** The expression of LC3B and p62 proteins after SC79 treatment in HeLa-CARMN cells. ns, *P* ≥ 0.05; *, *P* < 0.05; **, *P* < 0.01; ***, *P* < 0.001

KEGG enrichment analysis of the blue and lightyellow modules, obtained through WGCNA analysis of cervical tissues, revealed significant enrichment of the PI3K-Akt pathway (Fig. 1d and e). Using transcriptome analysis of HeLa-CARMN and -NC cells, we explored the downstream targets of CARMN (Fig. 3a and Table S8), and also identified the PI3K-Akt pathway as one of the most significantly enriched pathways (Fig. 3b). The role of Akt/mTOR pathway in the anti-tumor effect of CARMN was explored. IHC assay demonstrated that the expression of p-Akt (Thr308) and p-mTOR (Ser2448) was significantly down-regulated in the transplanted tumor tissues highly expressing CARMN (Fig. 3c). WB showed that their expression was also decreased in the cervical cancer cells overexpressing CARMN (Figs. 3d and S7a). The inhibited expression of p-Akt and p-mTOR can be rescued by the addition of SC79, an activator of Akt phosphorylation (Fig. S7b). Flow cytometry results showed a significant decrease in the proportion of cells in the G1 phase (Figs. 3e and S7c) in the SC79-treated group. The levels of Cyclin D1 (Fig. S7d) and Cyclin A1/A2 (Fig. S7e) were also rescued by SC79 treatment. Meanwhile, the abilities of colony formation were significantly increased after SC79 treatment (Figs. 3f and S7f).

Interestingly, we found a significant increase in autophagosome levels in the CARMN group compared with the control group using transmission electron microscope (TEM) (Fig. 3g). Given that mTOR is a well-established negative regulator of autophagy, a process involving the lysosomal degradation of cytoplasmic components and organelles [30], and that CARMN could regulate the Akt/mTOR pathway, we assumed that CARMN may trigger autophagy in CC by modulating the Akt/mTOR pathway.

Furthermore, we observed a significant upregulation of p62 expression, an indicator of reduced autophagic flux [31–33], and an increase in the LC3B-II protein levels with CARMN overexpression (Figs. 3h and i and S8a). These results implied that while CARMN stimulated an increase in the quantity of autophagosomes, it simultaneously impeded autophagic flux in CC cells [34]. The above results indicate that although CARMN can enhance autophagosome formation via the Akt/mTOR pathway, it impedes the fusion of autophagosomes with lysosomes, reducing autophagic flux, resulting in the accumulation of p62 and LC3B-II, ultimately impacting cellular malignant phenotypes.

Chloroquine (CQ) or Bafilomycin A1 (Baf-A1) prevents autophagy at the late stage by disrupting autophagosome-lysosome fusion, thus impairing autophagic flux [35]. Their treatment resulted in the accumulation of LC3B-II and p62 (Fig. S8b-c), in accordance with the effect of CARMN overexpression (Figs. 3i and S8a). The combination of CARMN with CQ or Baf-A1 resulted in the highest levels of p62 accumulation and LC3B-II among the groups (Fig. S8d-e). Impaired autophagic flux and disrupted autophagosome-lysosome fusion were further confirmed using an mRFP-GFP-LC3 reporter plasmid. Compared to the control group, both the number of red and green fluorescent dots increased in the CARMN overexpression group, indicating a blockade in autophagic flux [36], consistent with the trend observed after treatment with the positive control Baf-A1 (Fig. 3j). CARMN in combination with CQ or Baf-A1 further exacerbated cycle arrest in the G1 or G2/M (Figs. 3k and S8f) phase in CC cells, respectively, compared with CARMN overexpression alone. In addition, the capacities for cell colony formation (Figs. 3l and S8g) were significantly reduced in the CARMN group when combined with CQ. However, the combination with Baf-A1 did not result in a statistically significant change in colony formation capacity. The results for cell proliferation demonstrated that treatment with both CQ and Baf-A1 can significantly inhibit the proliferation of CC cells (Figs. 3m and S8h). Finally, the accumulation of p62 and LC3B-II induced by CARMN was reversed upon treatment with SC79 (Figs. 3n and S8i), which correlated negatively with the levels of p-Akt (Thr308) and p-mTOR (Ser2448). These findings suggested that CARMN triggers autophagic flux blockade through negative modulation of the Akt/mTOR signaling pathway.

**Fig. 4:**
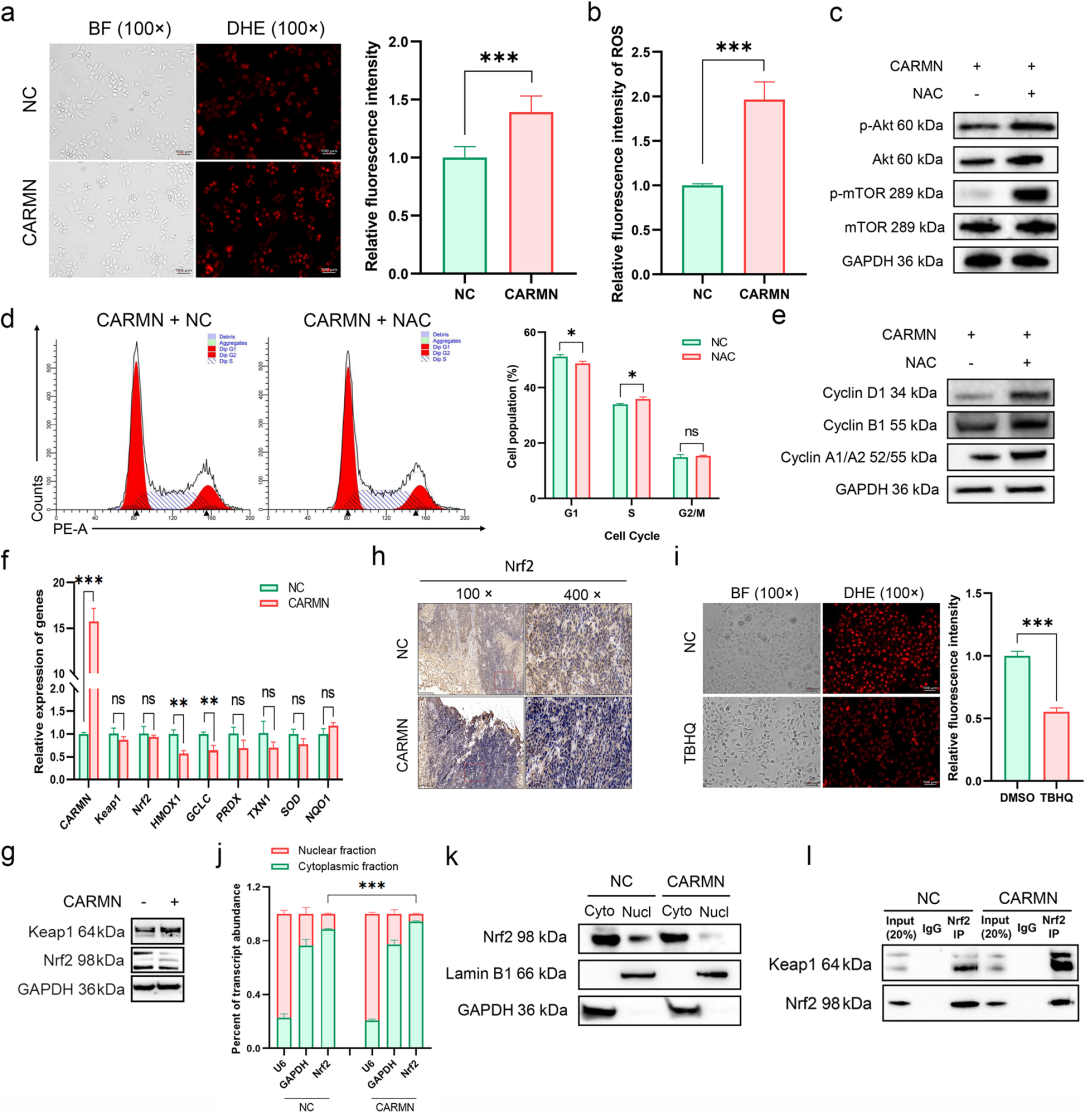
CARMN elevated ROS levels by inhibiting the nuclear translocation of Nrf2 to repress the Akt/mTOR pathway. **a** ROS levels using DHE probe with flow cytometry in HeLa cells with or without CARMN overexpression. **b** Detection of the Relative Quantity of ROS-Positive Cells by Flow Cytometry. **c** The effect of NAC on the Akt and mTOR expression in HeLa cells with CARMN overexpression. **d** Cell cycles in the HeLa-CARMN cells treated with NAC. **e** WB revealing expression of cell cycle-related markers in the HeLa-CARMN cells treated with NAC. **f**-**g** The expression of Keap1, Nrf2 and Nrf2 target genes in the HeLa-CARMN and -NC cells detected by qPCR (**f**) and WB (**g**). **h** IHC staining of Nrf2 in transplanted tumor tissues. **i** The effect of TBHQ on ROS level in HeLa cells. **j**-**k** Subcellular localization of Nrf2 mRNA (**j**) and protein (**k**) in the HeLa-CARMN and -NC cells. **l** The influence of CARMN on the binding of Nrf2 to Keap1 in the HeLa cell cytoplasm by co-IP. ns, *P* ≥ 0.05; *, *P* < 0.05; **, *P* < 0.01; ***, *P* < 0.001

We observed a significant increase in ROS levels in the CARMN group, as measured by the dihydroethidium (DHE) probe (Figs. 4a and b and S9a-b). Given that studies have reported that elevated ROS can inhibit Akt phosphorylation and induce autophagy [37, 38], we proposed that CARMN might influence autograph through the modulation of ROS levels. The significantly down-regulated expression of p-Akt and p-mTOR induced by CARMN returned to normal levels following N-acetylcysteine (NAC), a ROS inhibitor, treatment (Figs. 4c and S9c). Similarly, cell cycle arrest in the S phase (Figs. 4d and S9d) was alleviated with NAC treatment. The reduced levels of Cyclin D1 and Cyclin A1/A2 (Figs. 4e and S9e) were also rescued after NAC treatment. CCK-8 assays showed that the dampened proliferation abilities of CARMN-overexpressing cells were significantly recovered after NAC treatment (Fig. S9f-S9g). These results suggest that CARMN may induce cell autophagic flux blockade through the ROS/Akt/mTOR signaling pathway.

### CARMN elevated ROS levels by preventing Nrf2 nuclear translocation

Previous studies have shown that the Keap1-Nrf2-antioxidant response element (ARE) signaling pathway maintains intracellular redox homeostasis by regulating ROS levels [39, 40]. To determine whether the Keap1/Nrf2 complex participate in ROS production in the CARMN-overexpressing cells, their expression were detected. The findings revealed that Nrf2 protein levels were significantly reduced in cells overexpressing CARMN, although both Keap1 and Nrf2 mRNA levels remained unchanged (Fig. 4f and g). The expression of Nrf2 target genes, HMOX1 and GCLC, also decreased. Moreover, the protein expression of Nrf2 was decreased in the transplanted tumor tissues highly expressing CARMN (Fig. 4h).

We hypothesized that the observed reduction in antioxidant capacity in CARMN-overexpressing cells might be due to ferroptosis, a process known to impact glutathione peroxidase activity and promote ROS accumulation [41, 42]. To test this, we examined the expression of ferroptosis-related genes. However, the results did not support a ferroptosis-mediated mechanism for the increased ROS levels (Fig. S10a-b). Tert-butylhydroquinone (TBHQ), an activator of Nrf2 (Fig. S10c), significantly reduced the levels of ROS (Fig. 4i) and restored the expression of HMOX1 (Fig. S10d-e).

Further analysis showed that although there was no difference in total Nrf2 mRNA expression between the two groups (Fig. 4f), the proportion of Nrf2 mRNA in the nucleus was significantly reduced after CARMN treatment (Fig. 4j), suggesting that CARMN may promote the process of Nrf2 mRNA out of the nucleus. Although the proportion of Nrf2 mRNA was higher in the cytoplasm, its protein expression did not change markedly in the cytoplasm but was lower in the nucleus after CARMN treatment (Fig. 4k). This suggested that CARMN might impede the nuclear translocation of Nrf2 by promoting its degradation in the cytoplasm. Previous studies have shown that Keap1 targets Nrf2 for degradation via the ubiquitin-proteasome pathway [43, 44]. In our study, a co-IP assay demonstrated increased Keap1 binding to Nrf2 in the cytoplasm of CARMN-overexpressing cells (Fig. 4l). The levels of Nrf2-bound Keap1 and ubiquitinated Nrf2 increased in the presence of CARMN, as evidenced by their expression relative to total Nrf2 (Fig. S10f). These findings collectively suggest that CARMN may facilitate ubiquitination-mediated degradation of Nrf2 by promoting a stronger association between Keap1 and Nrf2 in the cytoplasm.

### CARMN suppressed the MAPK13-mediated MAPK cascade in CC

**Fig. 5:**
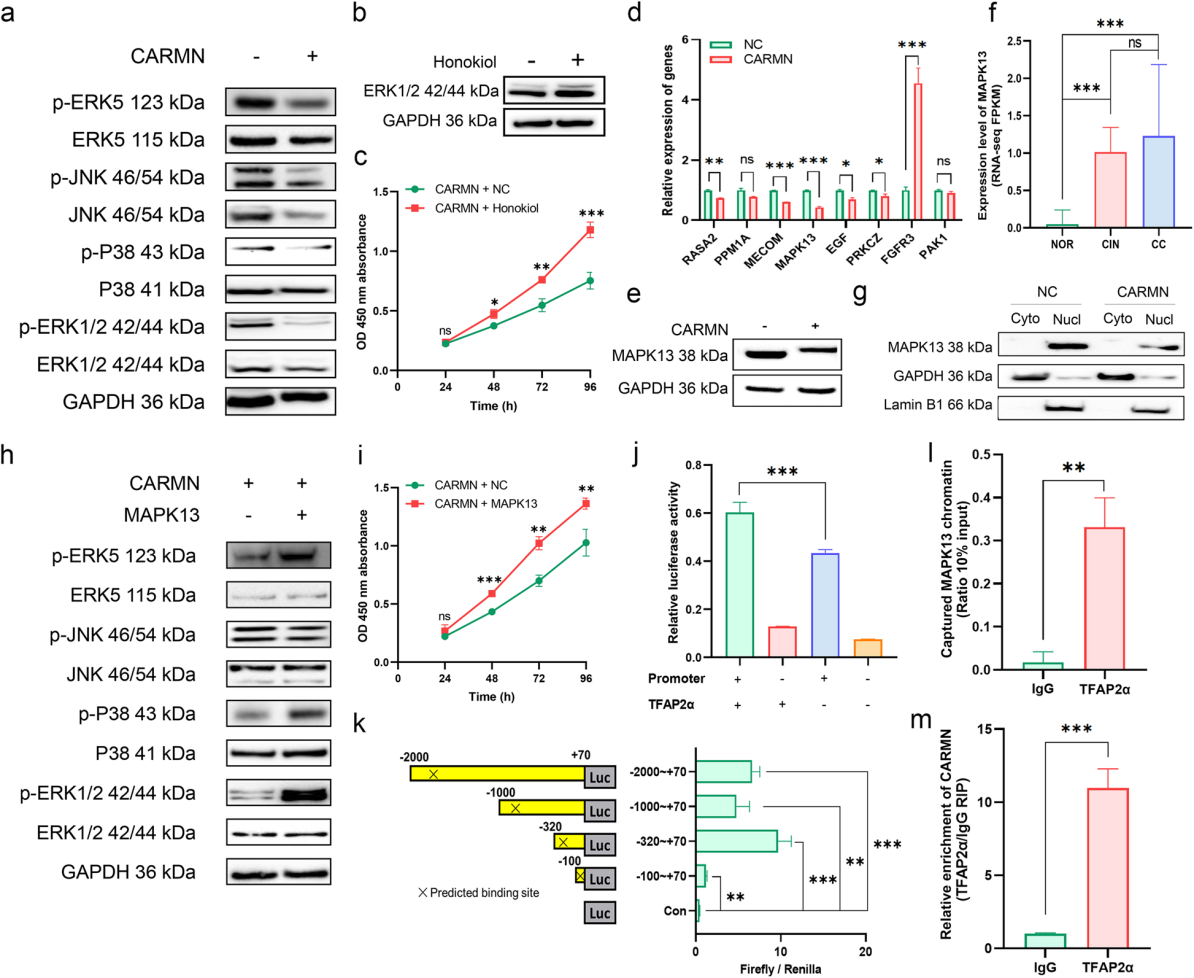
CARMN inactivated MAPK13-mediated MAPK cascade by repressing the transcriptional activation of MAPK13 via its sense chain. **a** The expression of MAPK cascade in the HeLa cells with or without CARMN overexpression. **b** Restoration of the MAPK signaling pathway in CARMN-overexpressing HeLa cells using honokiol. **c** CCK-8 assay results showed that honokiol treatment enhanced the proliferation ability of CARMN-overexpressing HeLa cells. **d** The expression of eight dysregulated genes related to MAPK signaling pathway in the HeLa-CARMN and -NC cells validated by qPCR. **e** MAPK13 expression of the HeLa-CARMN and -NC cells. **f** MAPK13 expression in the RNA-seq data of 30 cervical tissues. **g** MAPK13 expression in the cytoplasm and nucleus of the CC cells with or without CARMN overexpression. **h** The effect of MAPK13 on the expression of branches of MAPK cascade in the HeLa-CARMN and -NC cells. **i** The proliferation of HeLa-CARMN cells with or without MAPK13 overexpression by CCK8. **j** Luciferase reporter assay detecting the influence of TFAP2α on the transcriptional activity of MAPK13 promoter in the HeLa cells. **k** Luciferase reporter plasmids containing varied length of MAPK13 promoter regions were constructed to determine the potential binding sites of TFAP2α. **l** The levels of immunoprecipitated chromatin by anti-TFAP2α and control IgG in HeLa cells by ChIP assay. **m** RIP assay revealed the directly binding of CARMN and TFAP2α (HeLa). ns, *P* ≥ 0.05; *, *P* < 0.05; **, *P* < 0.01; ***, *P* < 0.001

As with the PI3K-Akt pathway, the MAPK pathway was also significantly enriched in the Lightyellow module and downstream targets of CARMN (Fig. 1e). The role of MAPK pathway in the anti-tumor effect of CARMN was also explored. Results showed that CARMN overexpression significantly inhibited the expression of four branches (i.e., ERK5, p-JNK, p-p38 and p-ERK1/2) of the MAPK cascade in CC cells (Figs. 5a and S11a). Additionally, treatment with the ERK1/2 activator honokiol can rescue the inhibition of cell proliferation caused by CARMN overexpression (Figs. 5b and c and S11b).

To elucidate the potential mechanism of CARMN regulating the MAPK signaling pathway, a total of 8 genes relevant to the MAPK signaling pathway, identified from differentiated expressed genes in CARMN-overexpressing cells (i.e., MAPK13, EGF, FGFR3, MECOM, PAK1, PPM1A, PRKCZ, RASA2; Fig. 5d), were verified by assessing their expression in the CC cells. Results showed that MAPK13 was the only gene that exhibited predominant lower expression in the CARMN-overexpressing cells (Figs. 5d and e and S11c). To investigate the role of miR-143 in this process, we knocked down miR-143-3p in CC cells stably overexpressing CARMN, results showed that the inhibition of MAPK13 by CARMN overexpression was not significantly restored, suggesting that miR-143 may play a limited role in CARMN’s regulation of MAPK13 (Fig. S11d). According to previous studies, MAPK13 plays an important regulatory role in inflammatory response, cell growth, and tumor formation and progression [45, 46]. RNA-seq analysis of 30 cervical tissues revealed that MAPK13 abundance significantly elevated during CC progression (Fig. 5f), which is opposite to the decrease in CARMN during CC progression (Fig. 1n). Through nucleocytoplasmic separation experiments, we found that MAPK13 was primarily distributed within the cell nucleus, consistent with the nuclear localization of CARMN (Fig. 5g), consistent with our previous report of CARMN [47]. In contrast to CARMN’s inhibition of the MAPK cascade, MAPK13 overexpression via transient transfection of plasmids (Fig. S11e) activated MAPK signal pathways, especially the branches of ERK5, p38 and ERK1/2 pathways (Figs. 5h and S11f). Furthermore, MAPK13 rescued the suppressed proliferation (Figs. 5i and S11g) and migration (Fig. S11h) abilities of CC cells overexpressing CARMN.

### CARMN repressed transcriptional activation of MAPK13 by interacting with TFAP2α via its sense chain

Our results showed that CARMN overexpression led to a significant downregulation of MAPK13 mRNA, indicating a potential regulation of MAPK13 by CARMN at the transcriptional level. We performed in silico analyses to screen potential TFs of MAPK13 using the PROMO and Alibaba 2.0 website (Table S9-S10). TFAP2α was predicted by both websites and had four predicted binding sites with high scores within MAPK13 promoter using JASPAR website (Table S11). Given its well-established functions [48], TFAP2α was selected for further analysis.

Upregulation or downregulation of TFAP2α in CC cells led to increased or decreased levels of MAPK13, respectively (Fig. S12a-b), but the expression of CARMN showed no consistent trend in different cervical cell lines (Fig. S12c). A reporter plasmid containing the promoter of MAPK13 (-2000 to + 70 bp) was employed to determine the specific binding site of TFAP2α to the promoter. The results revealed that TFAP2α significantly elevated luciferase activity of the reporter plasmids containing the MAPK13 promoter (Figs. 5j and S12d). Reporter plasmids with various truncated promoters of MAPK13 (-2000 ~ + 70, -100 ~ + 70, -320 ~ + 70, -100 ~ + 70) demonstrated that the plasmid with the − 320 ~ -100 bp promoter fragment had the highest luciferase activity among the four plasmids, indicating the most probable binding site of TFAP2α to MAPK13 (Figs. 5k and S12e). Notably, the overexpression of TFAP2α does not affect the changes in CARMN in CC cells (Fig. S12f). ChIP assays further confirmed this region was readily immunoprecipitated by anti-TFAP2α, demonstrating specific interaction of TFAP2α with the MAPK13 promoter in chromatin (Figs. 5l and S12g).

To test the hypothesis that CARMN down-regulates MAPK13 expression by competitively binding to TFAP2α, RIP and RNA-pulldown experiments were performed. The RIP results showed that CARMN was able to interact with TFAP2α (Figs. 5m and S12h), while the RNA-pulldown experiments revealed that TFAP2α could bind to CARMN (Fig. S12i). These findings raised the possibilities that CARMN suppresses transcriptional regulation of MAPK13 by competitively interacting with TFAP2α via its sense chain. Interestingly, we identified quite a number of TFAP2α target genes by ChIPBase dataset (Table S12), including ACE2, CCL5, DDX58, NCOA5, and RNASE4, among the downstream targets of CARMN, further supporting the notion that CARMN can regulate MAPK13 at the transcriptional level through its interaction with TFAP2α.

### Transcriptional dysregulation of CARMN in CC

**Fig. 6:**
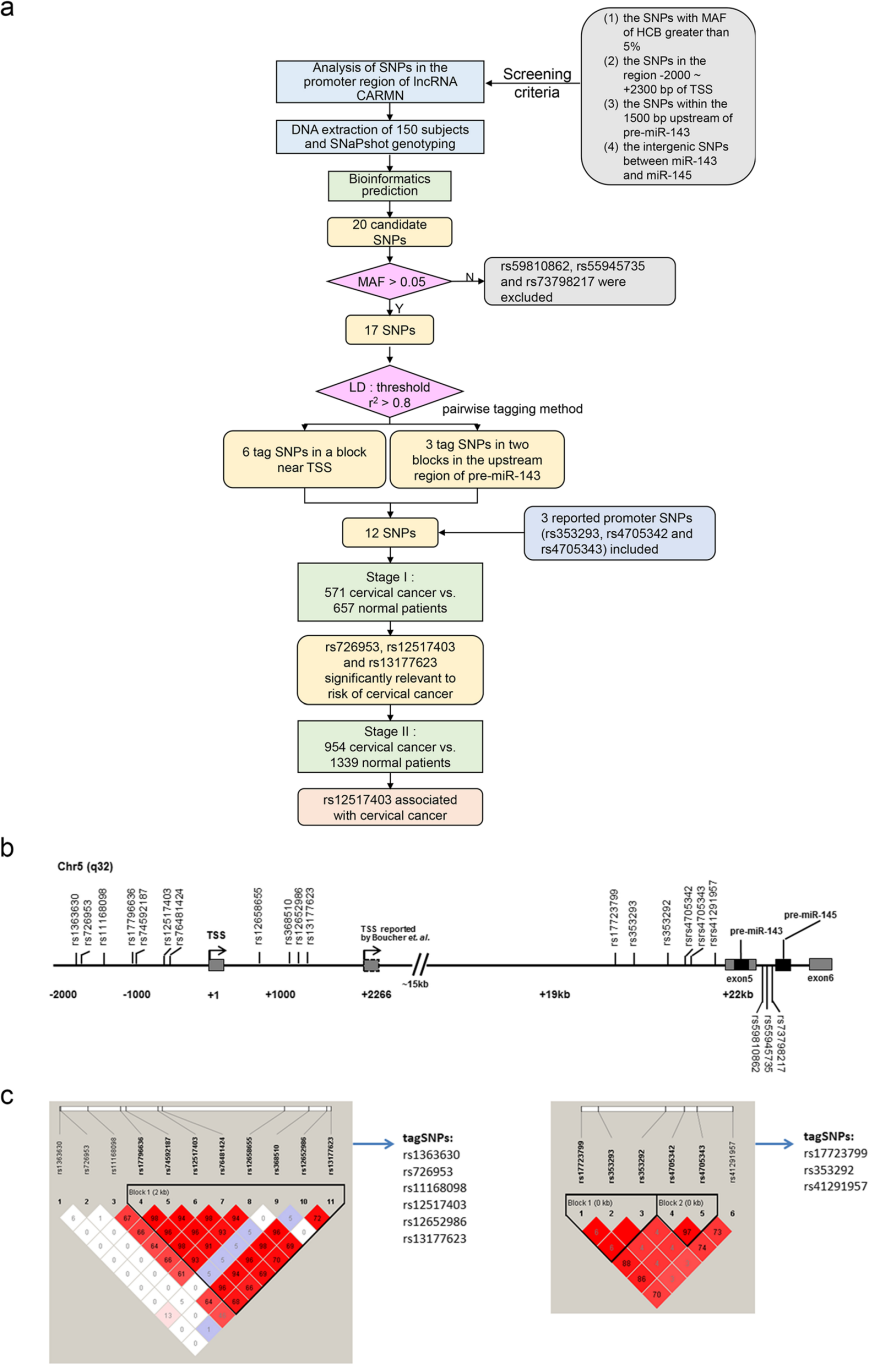
Illustration of genomic structure of CARMN and location of the 20 candidate SNPs. **a** Schematic diagram of screening CARMN promotor SNPs. **b** Genomic structure of CARMN and location of the 20 candidate SNPs. The grey box indicated pre-miR-143/145, while the black box indicated mature miR-143 and miR-145. TSS, transcriptional start site. **c** A total of 9 tagSNPs were selected from 17 candidate sites using Haploview 4.2

To uncover the molecular mechanisms behind CARMN reduction in CC, both transcriptional and post-transcriptional regulators were determined. Numerous studies have reported that promoter SNPs can affect gene expression by modulating TF binding to the promoter [49, 50]. The screening process of CARMN promoter SNPs was shown in Fig. 6a, with a total of 20 candidate promoter SNPs meeting the set criteria (Fig. 6b). These SNPs were subsequently genotyped in 150 healthy control subjects using the SNaPshot method, with 17 exhibiting a minor allele frequency (MAF) > 5% (Table S13). Further linkage disequilibrium (LD) analysis on the remaining SNPs identified 9 tagSNPs using Haploview 4.2 (r^2^ > 0.8, Fig. 6b). These 9 tagSNPs, in combination with 3 previously reported promoter SNPs (i.e., rs353293, rs4705342 and rs4705343) [51–53], were selected for subsequent analysis of their association with genetic susceptibility to CC in the Stage I study involving 571 CC patients and 657 healthy controls. The results showed that three SNPs (rs726953, rs12517403 and rs13177623) significantly correlated with CC susceptibility (*P* = 0.015, 0.0003 and 0.022, respectively) (Table S14). For enlargement of statistical power of the association study, more subjects were enrolled in the second-stage study, including 954 CC patients and 1339 healthy controls (Table 1). The aforementioned three risk-associated SNPs were further analyzed for their relationship with CC susceptibility. However, only rs12517403 (T > C) remained prominently relevant to risk of CC (dominant model: TC/CC vs. CC, OR = 1.34, 95% CI = 1.11–1.61; Table 1).

**Table 1:** Summary of association with CC risk for rs726953, rs12517403 and rs13177623 in the two stages

SNP	Stage	Cases^a^	Controls^a^	MAF^b^	OR (95% CI) ^c^			*P* ^||^
				Case/control	Additive/dominant/recessive model	Co-dominant model		Additive
						het^d^	hom^d^	
rs726953	Stage I	457/100/3	569/81/1	0.095/0.064	1.73 (1.25–2.39)	1.68 (1.18–2.40)	3.28 (0.31–34.72)	0.001
					1.71 (1.20–2.43)			
					1.83 (0.29–11.35)			
	Stage II	763/157/6	1087/217/11	0.091/0.091	1.09 (0.87–1.36)	1.13 (0.88–1.44)	0.87 (0.31–2.44)	0.452
					1.11 (0.88–1.42)			
					0.86 (0.31–2.39)			
rs12517403	Stage I	193/279/80	289/293/59	0.398/0.321	1.37 (1.14–1.64)	1.41 (1.09–1.83)	1.75 (1.17–2.62)	0.001
					1.48 (1.15–1.89)			
					1.50 (1.04–2.17)			
	Stage II	361/435/130	592/580/136	0.375/0.326	1.24 (1.08–1.43)	1.30 (1.07–1.58)	1.48 (1.10-2.00)	0.002
					1.34 (1.11–1.61)			
					1.29 (0.97–1.71)			
rs13177623	Stage I	279/238/43	376/234/41	0.289/0.243	1.29 (1.06–1.56)	1.37 (1.06–1.79)	1.44 (0.87–2.37)	0.01
					1.38 (1.08–1.78)			
					1.20 (0.76–1.91)			
	Stage II	466/378/76	717/506/93	0.288/0.263	1.13 (0.99–1.30)	1.16 (0.97–1.39)	1.22 (0.88–1.71)	0.078
					1.17 (0.99–1.39)			
					1.15 (0.83–1.59)			

^a^Genotypes were major homozygote/heterozygote/minor homozygote

^b^MAF, minor allele frequency in the controls

^c^Adjusted for age, parity and menopausal status in logistic regression model

^d^het, heterozygote vs. major homozygote; hom, minor homozygote vs. major homozygote

^||^*P* was for additive model

**Fig. 7:**
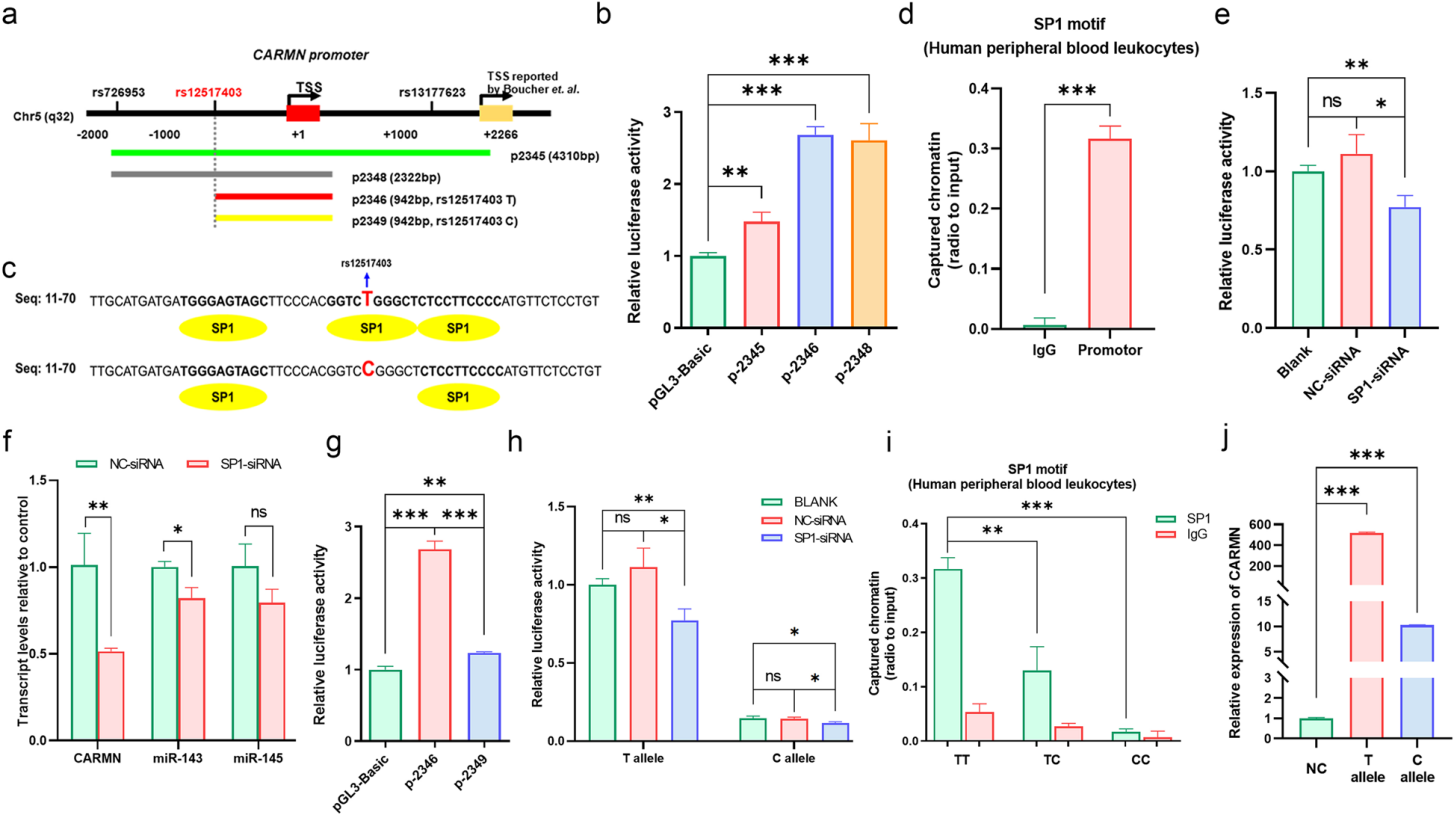
Transcriptional mechanism of CARMN downregulation in CC. **a** Schematic diagram of luciferase reporter plasmids containing varied length of CARMN promoter and three SNPs significantly related to CC susceptibility in the stage I association study. **b** Luciferase activities of reporter plasmids containing varied CARMN promoters in HeLa cells. **c** Schematic diagram showing the change of SP1-binding motifs around rs12517403 (T > C) within the CARMN promoter predicted by Alibaba2 website. **d** The respective levels of immunoprecipitated chromatin of human peripheral white blood cells by anti-SP1 and control IgG by ChIP assay. **e** Luciferase reporter assay detecting the effect of SP1 on the transcriptional activity of CARMN using SP1-siRNA in HeLa cells. **f** The expression of CARMN and miR-143/145 in the HeLa cells treated with or without SP1-siRNA. **g** Luciferase reporter assay detecting the effect of SNP rs12517403 T to C transition on the transcriptional activity of CARMN in HeLa cells. **h** The effect of SNP rs12517403 in combination of SP1 on the transcriptional activity of CARMN in HeLa cells. **i** The levels of immunoprecipitated chromatin of human peripheral white blood cells with different genotypes of rs12517403 by anti-SP1 and control IgG, respectively by ChIP assay. **j** The effect of rs12517403 polymorphism on CARMN expression in HeLa and SiHa cells. ns, *P* ≥ 0.05; *, *P* < 0.05; **, *P* < 0.01; ***, *P* < 0.001

Subsequently, three promoter fragments with varying sequences (from − 2000 to + 2310) were cloned into the pGL3-basic plasmid, namely p2345, p2348, and p2346 (Fig. 7a). Dual luciferase reporter assays demonstrated that p2346, containing the minimum promoter fragment, exhibited the strongest promoter activities among the three plasmids (Figs. 7b and S13a). In silico analysis identified several TFs within the p2346 promoter, with SP1 being the most predominant (Table S15). Interestingly, rs12517403 locates 640 bp upstream of the transcriptional start site (TSS), within the promoter region of p2346. Bioinformatics analysis indicated that transition from T to C allele could attenuate the binding affinity of SP1 (Fig. 7c). To confirm the critical role of SP1 in CARMN transcription, ChIP assay illustrated that CARMN promoter was readily immunoprecipitated by SP1 antibody but not IgG (Fig. 7d), indicating specific interaction of SP1 with the CARMN promoter. Moreover, p2346 was co-transfected with SP1 siRNA or NC (Fig. S13b-S13c), which showed a decreased luciferase activity after SP1 siRNA treatment compared with blank or NC (Figs. 7e and S13d). In addition, the effect of SP1 on CARMN expression was also validated in living cells, with both CARMN and its derived miR-143 down-regulated in CC cells after SP1 siRNA treatment (Figs. 7f and S13e). However, miR-145 was not significantly reduced in HeLa cells, indicating that miR-145 might not be closely co-transcribed with CARMN or miR-143 in HeLa cells [54].

To further test the hypothesis that rs12517403 influences the risk of CC through modulation of the binding affinity of SP1 to the promoter, a mutant vector containing rs12517403 C allele was constructed based on p2346 and named p2349 (Fig. 7a). The luciferase assays showed that p2349 exhibited lower luciferase activity than p2346 in CC cell lines (Figs. 7g and S13f), indicating that rs12517403 C allele dampened CARMN promoter activity. To determine whether the effect of rs12517403 on CARMN transcription relied on SP1, SP1 siRNA was co-transfected with either p2346 or p2349 into CC cells. Both reporter plasmids displayed reduced luciferase activities after SP1 siRNA treatment, and higher luciferase activities of p2346 compared with p2349 under any conditions were observed (Figs. 7h and S13g). The impact of rs12517403 on SP1 binding affinity was also assessed in vivo. Nine participants with three different genotypes, i.e., TT, TC and CC, were selected for ChIP assays. Chromatin with rs12517403 TT genotype was readily immunoprecipitated by SP1 antibody, and the binding of SP1 to the promoter was dramatically dampened with the increase of mutant C allele (Fig. 7i). The amount of captured chromatin with TC and CC genotypes was 42.0% and 5.6% of that with the TT genotype, respectively. The full-length CARMN containing the promoter with different rs12517403 genotypes was amplified and subcloned into the pCDNA3.1 overexpression vector. Results showed that the expression plasmids with C allele transcribed much lower amount of CARMN compare with their counterparts with T allele in CC cells (Figs. 7j and S13h). The above results suggest that the CARMN promoter polymorphism rs12517403 attenuated the binding affinity of SP1, resulting in decreased expression of CARMN at the transcriptional level, which conferred an increased risk of CC.

### Post-transcriptional regulation of CARMN in CC

**Fig. 8:**
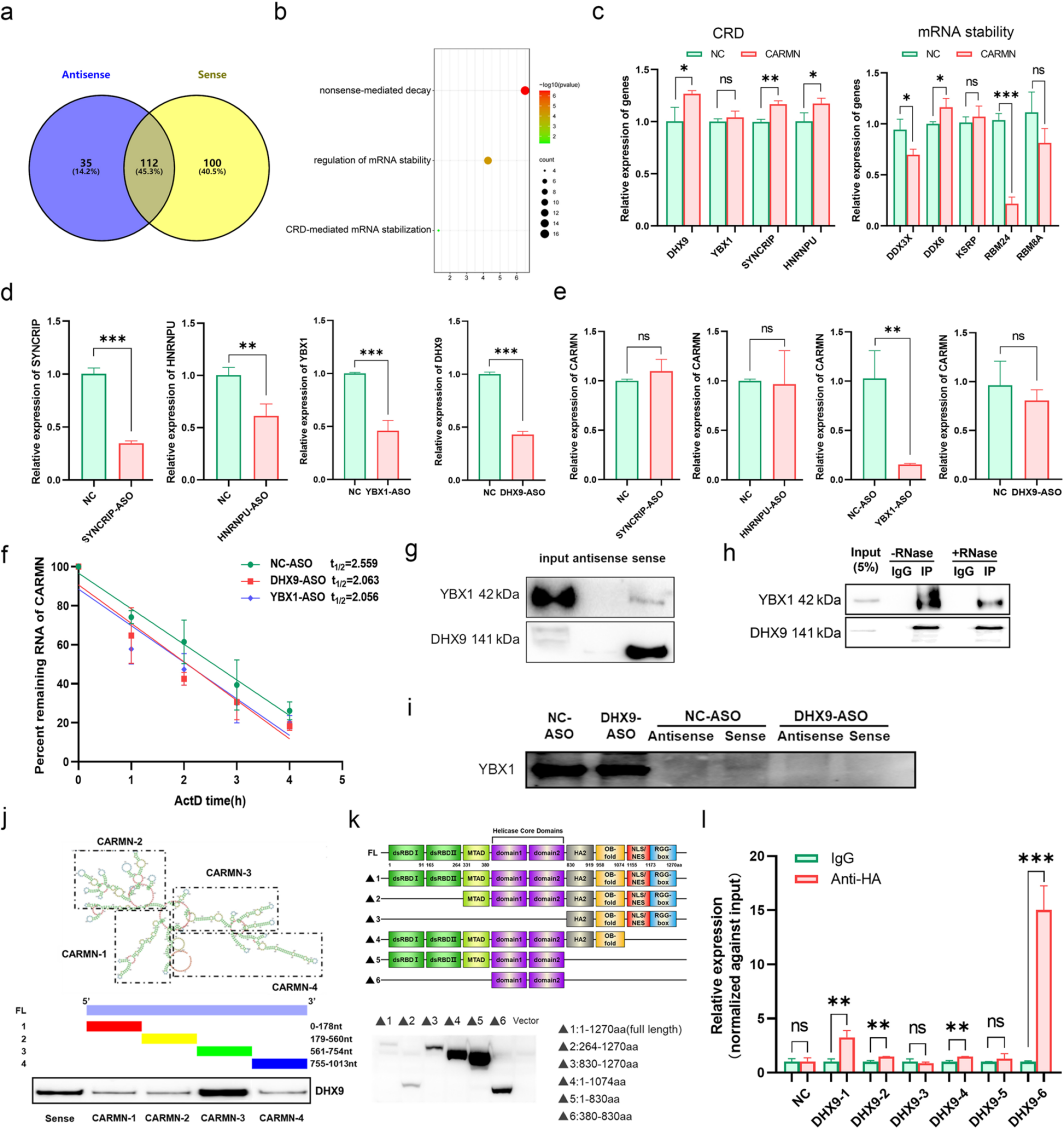
Post-transcriptional mechanism of CARMN downregulation in CC. **a** Venn diagram showing the number of RBPs bound to the sense and antisense strands of CARMN. **b** Bubble plot visualizing the GO enrichment analysis of RBPs. **c** The mRNA expression of core components of CRD (left) and mRNA stability (right) in the HeLa-CARMN cells. **d** The target RBPs were knocked down using ASO vectors, and the knockdown efficiency was assessed by qPCR in HeLa cell. **e** Detection of the effects of RBP knockdown on CARMN expression in HeLa cells by qPCR. **f** The RNA stability of CARMN treated with DHX9 ASO, YBX1 ASO, or NC in HeLa-CARMN cells. **g** RNA-pulldown showing the directly binding of CARMN to YBX1 and DHX9 via its sense strand in HeLa cell. **h** Co-IP showing the RNA-dependent interaction between YBX1 and DHX9 using RNase A in HeLa cell. **i** RNA-pulldown detecting the effect of DHX9 knockdown on the binding of YBX1 to CARMN sense strand in HeLa cell. **j** RNA-pulldown analyzing which sequence most critical for CARMN bound to DHX9 in HeLa cell. **k** WB detecting the constructed six truncated DHX9 proteins in HeLa cell. **l** RIP-qPCR analysis of CARMN expression which immunoprecipitated by antibodies of a series of truncated variants of DHX9 in HeLa cell. ns, *P* ≥ 0.05; *, *P* < 0.05; **, *P* < 0.01; ***, *P* < 0.001

The mechanism of post-transcriptional regulation of CARMN was also explored. RNA binding proteins (RBPs) interacting with CARMN were examined using RNA pull-down combined with liquid chromatography (LC)-mass spectrometry (MS). A total of 212 proteins were found to interact with CARMN’s sense strand, and 147 proteins with its antisense strand, respectively (Fig. 8a and Table S16). GO enrichment analysis of RBPs interacting with CARMN’s sense strand revealed three biological processes (BPs) involved in RNA stability, including coding region instability determinant-mediated mRNA stabilization (hereafter referred to as CRD), nonsense-mediated decay (NMD), and regulation of mRNA stability (Fig. 8b). This suggests the involvement of RBPs in regulation of RNA stability of CARMN. The expression of core CRD components, but not other BPs, consistently increased, suggesting an important role for CRD in the regulation of RNA stability of CARMN (Fig. 8c). The abundance of CARMN significantly reduced following YBX1 knockdown rather than other CRD components (Figs. 8d and e and S14). RNA decay analyses displayed that the half-life of CARMN was approximately 153 min; however, it was reduced by 30 min after DHX9 or YBX1 knockdown (Fig. 8f). RNA pull-down assays showed that CARMN strongly interacted with DHX9 but weakly interacted with YBX1, primarily through the sense chain (Fig. 8g). CRD components regulate RNA stability as complexes [55]. Co-IP assays verified the interaction between DHX9 and YBX1, with the association attenuated upon nuclease treatment, suggesting that DHX9 and YBX1 interaction was RNA-dependent (Fig. 8h). DHX9 knockdown nearly eliminated YBX1 binding to CARMN (Fig. 8i), implying that YBX1 might bind to CARMN mediated by DHX9 rather than directly binding.

Subsequently, specific interaction sites between DHX9 and CARMN were determined. Based on RNA secondary structure of CARMN predicted by RNAfold [56], its full sequence was divided into four parts potentially binding to DHX9 (Fig. 8j). RNA-pulldown analyses showed that CARMN-3, comprising nucleotides 561–754, was capable of immunoprecipitating most DHX9, emphasizing this sequence’s importance in binding to DHX9. Truncated DHX9 variants with different functional domains predicted by UniProt were also constructed (Fig. 8k). RIP-qPCR results showed that DHX9-1, -2, -4 and − 6 antibodies immunoprecipitated more CARMN than IgG, with DHX9-6 being the most significant (Fig. 8l). This suggests that the helicase core domain of DHX9 is essential for binding to CARMN. Furthermore, the observation of more immunoprecipitated CARMN in DHX9-1 and DHX9-6 compared with DHX9-2 and DHX9-5, respectively, supports the hypothesis that the dsRBD I/II domain may enhance the binding affinity of DHX9 to CARMN as the helicase core domain, while the MTAD domain demonstrated opposite results.

## Discussion

**Fig. 9:**
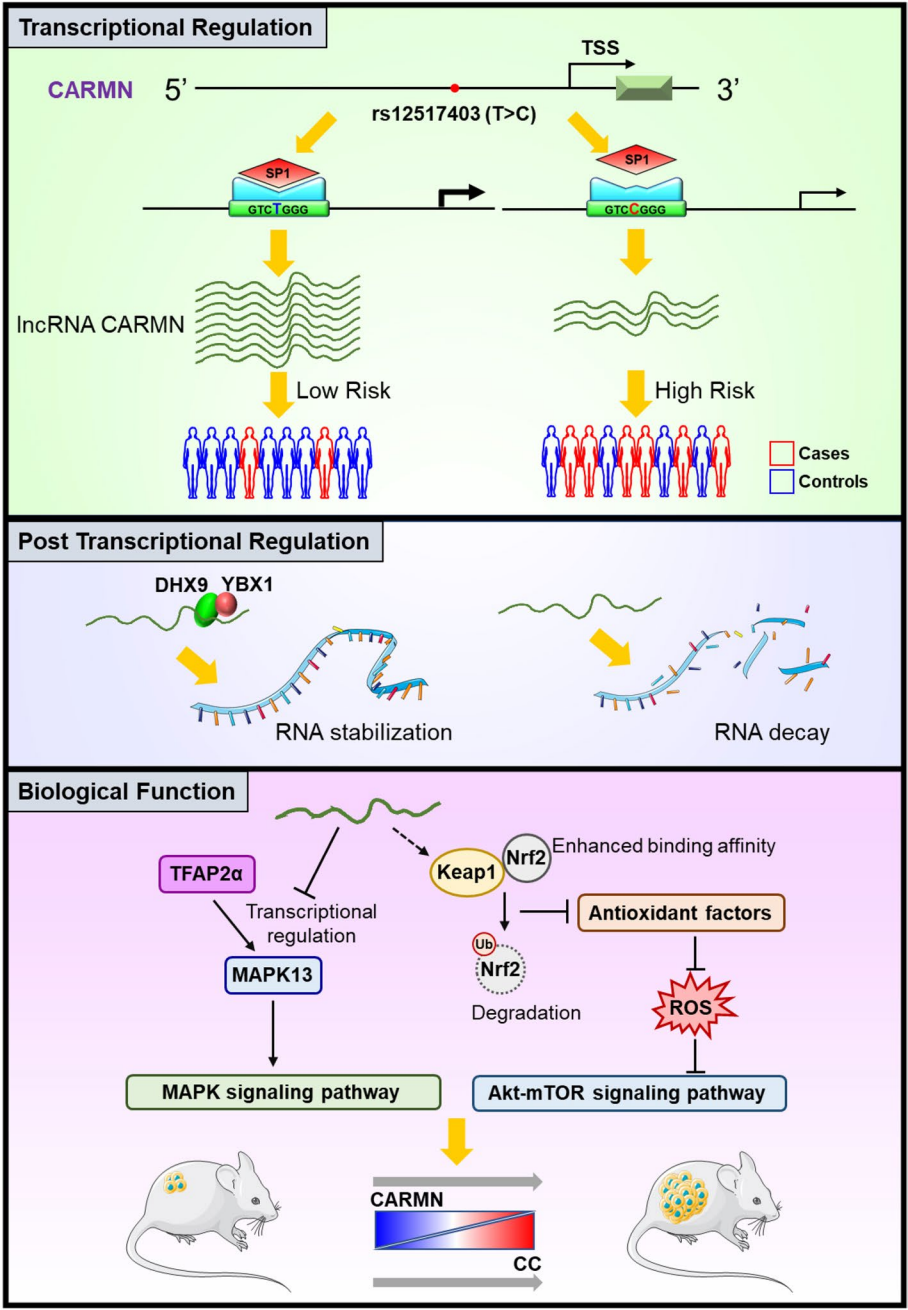
Schematic representation of the CARMN mechanism involving transcriptional and post-transcriptional regulation. This figure illustrated the molecular mechanism of CARMN regulation. At the transcriptional level, SNP rs12517403 (T > C) influenced the binding affinity of SP1 to the CARMN promoter region, modulating CARMN transcriptional activity, and was significantly associated with CC risk. At the post-transcriptional level, YBX1 and DHX9 bound to CARMN, synergistically enhancing its stability in cells. Functionally, CARMN inhibited MAPK13 expression by repressing TFAP2α transcription, thereby suppressing the MAPK signaling pathway. Concurrently, CARMN promoted Keap1-Nrf2 binding, facilitating Nrf2 ubiquitination and degradation, which indirectly inhibited Nrf2 nuclear translocation and antioxidant factor expression. This led to ROS accumulation, inhibiting the Akt-mTOR signaling pathway and suppressing the malignant progression of CC cells

This study leveraged 30 primary and 83 additional cervical tissues to validate the underexpression of CARMN and its involvement in the transition of NOR-CIN-CC. Furthermore, in vitro and in vivo studies indicated an anti-oncogenic role for CARMN in CC by regulating the ROS/Akt/mTOR and MAPK13-mediated MAPK pathways. The study also uncovered both genetic and epigenetic mechanisms underlying CARMN underexpression in CC. Specifically, a promoter polymorphism, rs12517403, located in CARMN, was found to attenuate the binding affinity of SP1, resulting in decreased transcriptional expression of CARMN. In an association study involving 954 CC cases and 1,339 healthy controls, this polymorphism conferred a 34% increased risk of CC. Furthermore, DHX9/YBX1, as part of the CRD-mediated mRNA stabilization complex, was shown to promote CARMN RNA stability at the post-transcriptional level (Fig. 9).

CARMN was initially identified in cardiac precursor cells, where it acted as a crucial regulator for cardiac cell differentiation and homeostasis [57], and contractile phenotype of vascular smooth muscle cells [58]. Dysregulation of CARMN has been linked to various diseases, such as atherosclerosis [58, 59], bone loss [60], and multiple cancers [20, 21, 61, 62]. Prior research has shown that CARMN (also known as miR143HG) inhibits cell proliferation and migration in Hirschsprung disease [63]), and has an anti-proliferative effect in several types of cancer [29, 61, 62, 64]. However, the role of CARMN in CC had not yet been thoroughly investigated. Using WGCNA, we identified gene modules related to the NOR-CIN-CC transition, and found that CARMN was a hub gene in two modules markedly associated with this transition. Analysis with ten machine learning diagnostic models revealed that CARMN is crucial in the NOR-CIN-CC transition and holds potential as a biomarker for the development and progression of CC. Both in vitro and in vivo experiments revealed CARMN’s anti-oncogenic role in CC, consistent with its function in other cancers. Our results showed decreased CARMN expression in CC and suggested its anti-oncogenic role through the regulation of ROS/Akt/mTOR and MAPK13-mediated MAPK signaling pathways.

In recent years, WGCNA and machine learning have emerged as essential tools for gene identification, greatly advancing the discovery of potential biomarkers across various diseases, including cancers [65–67]. Previous studies have applied these methods; for example, one study combined WGCNA with the LASSO algorithm to identify pivotal genes in CC [68]. Our study, however, took a more extensive approach by employing ten distinct machine learning algorithms, allowing for robust cross-validation and enhancing the reliability of our findings. Additionally, unlike studies that rely exclusively on publicly available datasets, we utilized a unique cohort from our own study population, which included not only normal and cancerous tissues but also a pre-cancerous lesion group (CIN). This dataset al.lowed us to delve deeper into disease progression, offering insights tailored to our study population with direct clinical relevance. The inclusion of the CIN group added a vital layer of biological complexity, enabling a more nuanced exploration of the transition from normal tissue to malignancy.

Autophagy is a highly conserved process in eukaryotic cells that helps maintain cellular homeostasis [69]. Tumors can hijack degradative ability of autophagy to favor their rapid growth with an adaptation to environmental challenges [69, 70]. Shi et al. reported that downregulation of lncRNA LINC00511 led to repression of proliferation and promotion of autophagy and apoptosis of CC cells [71]. In agreement with their findings, our study found that CARMN overexpression resulted in autophagy arrest via autophagic flux blockade, causing cell cycle arrest, apoptosis, and ultimately a reduced capacity for proliferation and colony formation. mTOR is a well-known key regulator of autophagy and is activated by Akt [72]. Intracellular ROS levels can down-regulate the Akt/mTOR signaling pathway, which activates autophagy [73]. In this study, KEGG pathway enrichment analysis identified the Akt/mTOR pathway as a key signaling pathway associated with CARMN. We found that CARMN could induce an increase in the number of cellular autophagosomes, potentially by activating intracellular ROS levels and inhibiting the Akt/mTOR pathway. However, it also induced autophagic flux blockade through unknown target(s). One potential target is p62 (also known as SQSTM1), which was present in the CARMN RNA-pulldown experiment (Table S16) and may act as one of the RBP of CARMN. Their interaction could enhance the stability of p62 RNA or protein, leading to an accumulation of p62 protein in cells overexpressing CARMN, which is a hallmark of cellular autophagic flux blockade [31–33]. The Keap1-Nrf2-antioxidant response element (ARE) signaling pathway is a master regulator of neutralizing cellular ROS and maintaining redox homeostasis [39, 40]. Recently, a few lncRNAs, such as HOTAIR, MALAT1, and MEG1 [74], have been identified as upstream regulators of Nrf2 activity. Our results showed that CARMN increased the binding affinity of Keap1 and Nrf2 in the cytoplasm and promoted ubiquitination-mediated Nrf2 degradation. This inhibited the entry of Nrf2 into the nucleus to initiate the transcription of antioxidant factor genes.

Our research also identified the MAPK signaling pathway as another critical pathway associated with CARMN. Numerous lncRNAs have been reported to regulate tumorigenesis and progression through the MAPK pathway [75]. Lin et al. reported that miR143HG exerts an anti-proliferative function partially by influencing the MAPK signaling pathway in hepatocellular carcinoma cells [61]. MAPK13, also known as p38δ, possesses a basilic function in the oncogenesis and cancer development, including proliferation, EMT, invasion, metastasis [76]. Our results demonstrated that CARMN inhibited proliferation in CC through regulating the MAPK cascade mediated by MAPK13. In addition, CARMN suppressed the transcriptional regulation of MAPK13 by interacting with TFAP2α via its sense chain.

The genetic mechanisms underlying CARMN underexpression in CC were determined. It has been well established that the promoter SNPs can alter the binding affinity of TFs, thus affecting gene transcription regulation. Guo et al. reported that the rs7463708 polymorphism, located at a distal enhancer that loops to the PCAT1 promoter, increases ONECUT2 binding, an androgen receptor-interacting TF, leading to PCAT1 upregulation during prolonged androgen treatment [77]. Hua et al. found that rs11672691, situated in the PCAT19-short promoter, possesses both promoter and enhancer functions, reducing NKX3.1 binding to the PCAT19-short promoter, resulting in weaker promoter but stronger enhancer activity [49]. So far, research on the effect of promoter SNPs on CARMN transcription is limited. We screened 20 potential promoter SNPs of CARMN using the SNaPshot method, and found that the transition of rs12517403 T to C allele weakened binding affinity of SP1 to the promoter, impairing transcriptional regulation and resulting in reduced CARMN expression. Furthermore, our association study displayed that the rs12517403 polymorphism increased CC risk by 34%. Liang et al. reported that the rs4705343 TC genotype was associated with a 37% increased risk of CESC, and the luciferase reporter assay showed lower luciferase activities for plasmids containing the C allele compared with the T allele [52]. Nevertheless, no significant association between rs4705343 and CC risk was observed in our stage I study (*P* = 0.15 for its genotype distribution between the cases and controls). The discrepancy may be due to genetic context dependent, as Liang’s and ours study subjects were from different regions of China. Xi et al. identified rs13177623 as a protective factor against glioma susceptibility [78]. We also examined the relationship between rs13177623 and risk of CC, but no significant association was found in the stage II study with more subjects included.

The epigenetic mechanisms responsible for CARMN underexpression in CC were also investigated. We employed RNA pull-down combined with LC-MS to identify CARMN-binding RBPs. Our findings revealed that YBX1 reduction decreased RNA stability of CARMN, with DHX9 acting as a scaffold to bridge them. Moreover, the dsRBD I/II domain and the helicase core domain of DHX9 enhanced binding affinity to the 561–754 nucleotides of CARMN. YBX1 and DHX9 have been extensively studied in cancer research. Evidence suggests that YBX1, DHX9, and other CRD components are essential for stabilizing c-myc mRNA via the CRD [55]. YBX1 cooperates with IGF2BPs to stabilize BCL2 and MYC mRNA [79]. Ding et al. reported that DHX9 was upregulated in CC tissue and promoted CC cell motility and angiogenesis. Lnc-CCDST promotes DHX9 degradation through the ubiquitin proteasome pathway by serving as a scaffold to facilitate MDM2 and DHX9 complex formation [80]. While our results align with the reported role of YBX1 in promoting RNA stability, further research is needed to clarify precise function of DHX9 in CC.

Some limitations should be acknowledged. Firstly, the exact regulatory mechanism by which CARMN inhibits the nuclear translocation of Nrf2 remains unclear. We observed that CARMN reduced the abundance of Nrf2 in the nucleus and increased the binding affinity between Keap1 and Nrf2 in the cytoplasm. However, since CARMN is primarily located in the nucleus, the direct mechanism by which it regulates the binding affinity of Nrf2 and Keap1 in the cytoplasm requires further clarification. Secondly, multiple regulatory mechanisms may contribute to CARMN downregulation in CC, including TF and RBP modulation, histone modification, and m^6^A RNA methylation, which regulate CARMN expression at transcriptional or post-transcriptional levels. Further investigation into these critical regulatory mechanisms is warranted. Thirdly, a portion of the molecular and cell experiments in this study were performed on HeLa cells, which are derived from adenocarcinoma. While HeLa cells are a widely used model for studying CC, they may not fully reflect the biology of squamous CC, which is the most common subtype. This limitation will be addressed in future studies by incorporating experimental results from squamous carcinoma cell lines to improve the relevance and generalizability of our findings. Finally, our epidemic association analysis lacks information on HPV, a necessary cause of CC. Since HPV is not required for CC diagnosis (NCCN Clinical Practice Guidelines in Oncology: Cervical Cancer), patient records often omit this information. Nevertheless, we believe that HPV infection has a minimal impact on the association between rs12517403 and risk of CC, as HPV infection is detected in 99.7% of CC cases [81] and 75% of all sexually active individuals at some point during their lifetimes [82].

In summary, we employed WGCNA to identify gene modules corresponding to the NOR-CIN-CC transition and discovered that CARMN functions as a hub gene and tumor suppressor in CC by modulating both ROS/Akt/mTOR and MAPK13-mediated MAPK signaling pathways. Our research on CARMN underexpressed in CC revealed that the rs12517403 polymorphism attenuated SP1 binding affinity to the promoter region, leading to reduced transcriptional activity, and thus lower CARMN and its derived miR-143 expression, consequently contributing a 34% increased risk of CC. In addition, YBX1 was found to stabilize CARMN, with DHX9 acting as a scaffold to bridge them. Further in-depth functional and association studies with larger sample sizes are warranted to confirm and extend our findings.

## Conclusion

CARMN mediates its anti-cancer effects through the inhibition of the Akt-mTOR and MAPK signaling pathways, as well as the suppression of autophagic flux. Its expression is regulated by the rs12517403 polymorphism and the YBX1/DHX9 complex. CARMN holds potential as a biomarker and therapeutic target for cervical cancer.

## Supplementary Information

Supplementary Material 1.

## Data Availability

The data described in this article can be freely and openly accessed at Gene Expression Omnibus database (GEO, https://www.ncbi.nlm.nih.gov/gds/), Genotype-Tissue Expression Project (GTEx, https://www.gtexportal.org/), and The Cancer Genome Atlas (TCGA, https://portal.gdc.cancer.gov/). For other sequencing data, please refer to the supplementary data or contact the corresponding author at any time.

## Abbreviations

ASO: Antisense Oligonucleotides
Baf-A1: Bafilomycin A1
CAC: Cervical Adenocarcinoma
CARMN: Cardiac Mesoderm Enhancer-Associated Noncoding RNA
CC: Cervical Cancer
CESC: Cervical Squamous Carcinoma
CIN: Intraepithelial Neoplasia
CQ: Chloroquine
CRD: Coding Region Instability Determinant
DELAX: Descriptive Machine Learning Explanations
DHE: Dihydroethidium
GEO: Gene Expression Omnibus
GEPIA: Gene Expression Profiling Interactive Analysis
GTEx: Genotype-Tissue Expression
LD: Linkage Disequilibrium
MAF: Minor Allele Frequency
NMD: Nonsense-Mediated Decay
NOR: Normal
RBP: RNA Binding Protein
SHAP: Shapley Additive Explanations
TCGA: The Cancer Genome Atlas
TEM: Transmission Electron Microscope
TSS: Transcriptional Start Site
WGCNA: Weighted Gene Coexpression Network Analysis
