# Supplementary material for: Transcriptional and post-transcriptional regulation of CARMN and its anti-tumor function in cervical cancer through autophagic flux blockade and MAPK cascade inhibition
Source: J Exp Clin Cancer Res. 2024 Nov 19;43:305. doi: 10.1186/s13046-024-03229-y (PMC11575122; doi:10.1186/s13046-024-03229-y)
Supplement: Supplementary file 1 — Supplementary Material 1. [file 13046_2024_3229_MOESM1_ESM.zip › Supplementary_materials_ESM.docx]

Supplementary Materials for

**Transcriptional and Post-Transcriptional Regulation of CARMN and Its Anti-Tumor Function in Cervical Cancer through Autophagic Flux Blockade and MAPK Cascade Inhibition**

Xing Zhang^1,†^, Wenjing Yan^1,†^, Hua Jin^2,†^, Bingjia Yu^3^, Hao Zhang^4^, Bo Ding^5^, Xue Chen^1^, Yan Zhang^1,6^, Qianqian Xia^1^, Dan Meng^1^, Jing Hu^1^, Haohan Liu^1^, Yamei Nie^1^, Fengying Liu^1^, Yun Zheng^1^, Yiran Lu^1^, Juan Wang^2^, Mulong Du^7^, Meilin Wang^7^, Evan Yi-Wen Yu^1,*^, Xiuting Li^3,*^, Shizhi Wang^1,*^

**Shizhi Wang**. Email: shizhiwang2009@seu.edu.cn

**This PDF file includes:**

Supplementary Materials and Methods

Supplementary Table. S1-S16

Supplementary Figure Legends of Fig. S1-S14

**Supplementary Materials and Methods**

**Ethics statement**

The study was conducted according to guidelines approved by the Institutional Review Board of Southeast University. All participants signed informed consent.

**Study subjects**

In this study, all cervical tissues (30 tissues for RNA-seq and 83 tissues for qPCR) used were fresh specimens obtained from the human cervix. The specimens were collected from patients undergoing surgery at Southeast University Affiliated Zhongda Hospital between 2018 and 2019, with informed consent and agreement. The inclusion criteria of the case group were: 1) patients with a pathological and histological diagnosis of CC; 2) no prior chemotherapy or radiotherapy treatment; 3) exclusion of patients with metastatic cancer from other origins; and 4) provision of informed consent by patients. The control group consisted of individuals with no history of gynecological tumors, no biological relationship to the case group, and age-matching with the case group subjects. The tissues were cut into small pieces (< 3 mm) and immersed in RNA Keeper Tissue Stabilizer (Vazyme, China) to prevent RNA degradation. After overnight storage at 4°C, they were temporarily stored at -80°C, awaiting subsequent processing for RNA extraction. Additionally, for RNAScope analysis, the tissues were paraffin-embedded, sectioned, and stained.

The details of subjects for association study have been described elsewhere [1]. Briefly, a total of 571 cases and 657 controls were collected for the primary association study in the first stage. More subjects were enrolled to enlarge the statistic power, and the final sample size reached 954 cases and 1339 controls in the stage II study. The samples were mainly from outpatients and inpatients at the hospitals in Southeast University Affiliated Zhongda Hospital and Nantong Tumor Hospital since January 2007.The inclusion and exclusion criteria for the samples were consistent with those outlined for the population in the preceding paragraph. The demographic and clinical characteristics of all study subjects was shown in Table S2.

In the genotyping section of the study, peripheral blood samples were collected from 2,293 participants. After obtaining informed consent, 2 ml of leftover blood from each participant's routine blood tests was preserved. The blood samples were treated with 2% disodium ethylenediaminetetraacetic acid (EDTA) as an anticoagulant, followed by erythrocyte removal and leukocyte lysis for subsequent DNA extraction and genotyping.

**RNA-seq and data processing**

The total RNA was extracted from frozen tissue specimens or cells using TRIzol reagent (Thermo, USA). Prior to this, cervical tissues were treated with RNA Keeper Tissue Stabilizer to prevent RNA degradation and then ground to a powder using a grinder, followed by the same treatment as applied to the cells. RNA quality was assessed by ensuring an absorbance ratio (A260/A280) between 1.8 and 2.0. Additionally, sample quality was evaluated through quality control (QC) and PCA analysis during the sequencing process. RNA samples that passed QC were subsequently subjected to high-throughput sequencing on the Illumina HiSeqTM 4000 platform, conducted by Gene Denovo Biotechnology Co. (Guangzhou, China). Raw sequences were filtered by fastp (version 0.18.0) and mapped to the human reference genome (Ensembl_release95) using HISAT (version 2.2.4). The mapped reads of each sample were assembled via StringTie (version 1.3.1) and a FPKM (fragment per kilobase of transcript per million mapped reads) value was calculated to quantify the expression abundance of each transcription region using RSEM software. Discriminant mRNAs, lncRNAs and differences between groups were analyzed using Bayes moderated t test (limma) with Benjamini Hochberg false discovery rate (FDR) at *P* < 0.05, unless otherwise specified. A two-fold cut-off difference was applied to select the up- and down-regulated miRNAs, unless otherwise specified.

**Weighted gene co-expression network analysis (WGCNA)**

A co-expression network for mRNAs and lncRNAs was performed using R package WGCNA (version 1.72-5) [2]. In this study, WGCNA was conducted based on whole-transcriptome sequencing data from 30 cervical tissue samples, consisting of 10 CC, 10 CIN, and 10 NOR. Before conducting the analysis, genes with low expression levels in cervical tissues (mean expression value < 1) were excluded, leaving a total of 15,935 genes and transcripts for further analysis. Briefly, a matrix of pairwise Pearson correlation of all genes was calculated by WGCNA, and it was converted into an adjacency matrix through a power adjacency function in the package. The gene-gene adjacency was considered as the distance between genes, which would be served as the basis for subsequent grouping. The power β = 9 was chosen based on a scale-free fit R^2^ > 0.84 for samples to obtain a high-confidence network. All gene pairs were divided into 34 modules after the above steps, and the expression of each module was quantified with its priority component (Eigengene). Module in this process was defined as a cluster of densely connected genes. Next, among these modules, we focused on the most significant one related to CC, and its potential biological pathways were predicted through functional enrichment analysis. Finally, the transcription factor (TF) prediction analysis was performed to quarry the credible TFs which might bind to the target RNAs using R package TFBSTools (version 1.42.0), and a TF network was built based on the results. These results were visualized by Cytoscape (version 3.9.1).

**Machine learning**

The data we used for machine learning were from the GEO database. There are numerous cervical cancer-related datasets in the GEO database, and we screened the relevant ones based on the following criteria: i) only expression profiling chips were included, excluding high-throughput sequencing data; ii) the gene chip manufacturers had to be the same; and iii) the gene chip platforms had to be consistent. These criteria helped exclude unreliable results that may arise from differences in principles, methods, and detection depth when combining gene expression data. Ultimately, the four cervical cancer datasets included in our study met these consistent criteria: all were expression profiling microarrays, all were from Affymetrix GeneChip, and all used the GPL570 platform (Human Genome U133 Plus 2.0). The technical consistency of the datasets ensured the reliability and accuracy of the data merging and subsequent analyses.

The four datasets included the entire dataset from GSE63514, cervical tissue-related data from GSE6791, a subset of GPL570 data from GSE27678, and normal cervical tissues from GSE75132, totaling 198 samples for the new dataset. Prior to merging the datasets, each dataset was normalized separately, followed by batch effect correction of the normalized data. Principal Component Analysis (PCA) was performed to assess the consistency of the samples before and after processing. The construction of the new dataset was then completed. The SMOTE method was employed to balance the training data, and five-fold cross-validation was used for model training, with grid search employed for hyperparameter optimization. For outcome variable classification, CIN3 and worse were grouped together as CIN3+[3-5], resulting in four categories: Normal, CIN1, CIN2, and CIN3. In this study, ten machine learning algorithms, labeled Decision Tree (DT), Random Forest (RF), Extreme Gradient Boosting (XGBOOST), Elastic Net Regression (ENR), Support Vector Machine (SVM), Multi-Layer Perceptron (MLP), Light Gradient Boosting Machine (Lightgbm), k-Nearest Neighbors (KNN), Logistic Regression (LR), and Stacking Ensemble (Stacking), were employed. Based on areas under the curve (AUC) analysis, DT, RF, SVM, and LR models performed less effectively in sample classification compared to other models. Therefore, we combined these four models to create an ensemble model, Stacking. The first layer of Stacking consists of the four aforementioned models, with their output values used for subsequent learning by a LASSO classifier in the second layer. Finally, this analysis was performed using R software, with dataset normalization carried out using the “limma” package (version 3.60.3), batch effect correction using the “sva” package (version 3.50.0), DALEX analysis using the “DALEX” package (version 2.4.3), and SHAP explanations using the “shapley” package (version 0.3). Other model constructions were based on the “tidymodels” package (version 1.2.0). Additional details can be referenced in our previous publications[6].

**Cell culture, plasmid and lentiviral vectors**

CC cell lines HeLa (RRID: CVCL_0030) and SiHa (RRID: CVCL_0032) were cultured in a 37℃, 5% CO_2_ incubator in DMEM/HIGH glucose Complete medium (KeyGEN BioTECH, China) containing 10% FBS (Sigma, USA) and 1% penicillin-streptomycin. C33A (RRID: CVCL_1094) was cultured in MEM/Minimum essential medium (KeyGEN BioTECH, China), and other culture conditions were consistent with HeLa cells. Cells after three generations of culture were regularly tested for mycoplasma to ensure mycoplasma-free. Chemicals used for cell treatment were listed in Table S4.

The plasmid and lentiviral vectors, listed in Table S5, were employed for RNA interference or gene overexpression, which were purchased from Genechem (Shanghai, China). The packaging plasmids pHelper 1.0 and pHelper 2.0 were also from Genechem, and lentiviral packaging was performed in 293T cells.

For stable transduction, cells were infected by lentiviral particles in combination of polybrene and incubated for 12 h. Thereafter, the culture medium was replaced with complete medium containing puromycin (5μg/ml), and then replaced every 2 days until 1 week after all the control cells had died. Positive cells were maintained in 1μg/ml puromycin for 2 weeks and later frozen down until use. In this study, we used CC cell lines HeLa, SiHa, and C33A for sequential lentiviral infection to construct stable cell lines overexpressing CARMN, which were utilized for subsequent investigations. Plasmid transfection was performed using Lipofectamine 2000 (Invitrogen, USA). After 6 h of transfection, the cells were washed and replaced with complete medium, and cells were collected 48 h later for subsequent experiments. The ASO transfection process was similar to that of the plasmids, using an ASO transfection kit (Ribobio, China), with a 48-hour incubation period.

**Xenograft model**

In this study, the mouse strain used was BALB/c, purchased at 4 weeks of age. The female mice weighed between 16 and 20 grams, with six mice per group. The cell line injected was a HeLa cell line stably overexpressing CARMN. The cells were maintained in culture for no more than 10 passages. Prior to injection, the overexpression efficiency of CARMN was verified via qPCR to ensure the accuracy of the experiment. Tumor long and short diameters were measured every 3 days, and the tumor volume was calculated using the formula V = 1/2 (a×b^2^). V represents the volume of the transplanted tumor, a and b represent the long and short diameters of the transplanted tumor, respectively.

**Plate colony formation assay**

Cells were seeded in 6-well plates at a density of 500-1000 cells/well (HeLa cells 500 cells/well, C33A cells 1000 cells/well) and cultured in a normal medium for 14 days. Cells were then washed twice with PBS, fixed with formaldehyde for 20 min, and stained with 0.1% crystal violet. Cells were washed with PBS to remove excess dye. Plates were air-dried, and colonies were photographed under a microscope and counted using ImageJ software.

**Transwell assay**

The migration assay was performed using the 12-well Transwell chambers (Corning Costar, USA) with a pore size of 8 μm. Cells (1×10^5^/well) in the serum-free medium were added to the upper compartment of chamber, while culture medium supplemented with 15% FBS adding to the lower compartment. After 24 h incubation, cells having traversed to reverse face of the chamber membrane were fixed, stained and counted under a microscope. The invasion assay (5×10^5^/well) was done under the same conditions as the migration assay except for chamber membranes precoated with Matrigel (BD, USA).

**Flow cytometry for cell proliferation and apoptosis**

Briefly, cells were digested and washed with PBS, fixed with 70% ethanol, resuspended in PBS containing PI/RNase, and analyzed by flow cytometry. Cell apoptosis was detected using Annexin V-PE/7-AAD Apoptosis Detection Kit (A213-01, Vazyme, China) and Annexin V-Alexa Fluor 647/PI Cell Apoptosis Detection Kit (40304ES50, Yeasen, China). Briefly, cells were stained with Annexin V-PE/7-AAD at room temperature in the dark. The apoptosis rate was analyzed by flow cytometry.

**RNAScope**

To visualize the target RNA, RNAScope probes (#493421; ACD, USA) were employed to bind to RNA within the tissues, and signal amplification was utilized to visualize CARMN within the tissues. The tissue section was treated with the target repair reagent at 100℃ for 15 min and then incubated with hydrogen peroxide at room temperature for 10 min to expose RNA. After drawing a hydrophobic barrier around the tissue, the section was rinsed with distilled water and ethanol, followed by incubation with protease III at 40℃ for 30 min in a humid environment. The probe was added within the barrier and incubated at 40℃ for 2 h. The section was then sequentially rinsed and hybridized with Amp1 through Amp6. Finally, the section was stained with DAB and examined under a microscope.

**Chromatin immunoprecipitation (ChIP)**

Genomic DNA from human peripheral blood leukocytes or CC cells were used for ChIP experiments using EZ-Magna ChIP™ G Kit (Merck Millipore, Germany). Briefly, chromatin fragments were obtained by sonication of the fixed chromatin, followed by immunoprecipitation using antibody against SP1 (#9389, dilution ratio 1:100, CST, USA), TFAP2α (#13019-3-AP, dilution ratio 1:100, Proteintech, USA) or control IgG. Subsequently, the protein was digested, and the precipitated targeted chromatin fragments were collected and quantified by qPCR assays.

**Immunoprecipitation assay (RIP)**

RIP was performed with RIP™ RNA-Binding Protein Immunoprecipitation Kit (Merck Millipore, USA) as the manufacturer’s instructions. Briefly, the cell lysate was incubated at room temperature for 30 min to prepare the magnetic bead-target protein complex. Next, the target antibody and the complex were mixed and incubated at 4°C overnight to obtain the RBP immunoprecipitated complex. Proteinase K was used subsequently to degrade proteins. Finally, RNA purification and RNA concentration determination were executed and PCR amplification was performed. In this study, the RIP antibodies used were against TFAP2α and tag protein HA. The abundance of CARMN bound to the target proteins was detected via qPCR.

**RNA-pulldown**

Using pcDNA3.1-CARMN as the template, a DNA template containing the T7 RNA polymerase promoter sequence was obtained by PCR amplification. Primers used to prepare templates were as follows: taatacgactcactatagggagctcatataaggtaaaaggcagag (F) and TGCCAAGGGCTGGAACTTTATTATT (R) for sense chain, taatacgactcactatagggTGCCAAGGGCTGGAACTTTATTATT (F) and agctcatataaggtaaaaggcagag (R) for antisense chain. Next, in vitro transcribed CARMN was obtained using MEGAscript Kit (Life Technologies, USA). Then, the Pierce Magnetic RNA-Protein Pull-Down Kit (Thermo scientific) was used to perform RNA pull-down experiments to adsorb proteins interacting with CARMN as follows. First, the biotin probe-labeled CARMN was obtained according to the procedure of Pierce RNA 3’ End Desthiobiotinylation Kit (Thermo scientific). Next, 50 pmol of biotin-labeled RNA was incubated with 30 μl of streptavidin magnetic beads for 30 min at room temperature to ensure the tight binding of RNA to magnetic beads. Then, the beads were washed with 100 μl 1X Protein-RNA Binding Buffer and incubated with whole HeLa cell lysate (100 μg per sample) for 2h at 4°C with rotation. After that, 50 μl of Elution Buffer was added and incubated at 37°C with rotation for 30min to obtain CARMN-bound protein complexes. Pulldown proteins were then identified by mass spectrometry and verified by WB analysis.

**Liquid chromatography (LC)-Mass spectrometry (MS) for protein identification**

LC/MS analysis for protein identification was conducted by Wuhan Genetic Engineering Co. Ltd. (China). Sample preparation consisted of enzymatic digestion of protein gels and zip desalting, followed by dilution of the peptide samples. The samples were then loaded into the mass spectrometer using the Triple TOF 5600+ LC/MS system (AB Sciex, USA) coupled with the Eksigent nanoLC system (AB Sciex, USA). For protein identification, the Paragon algorithm in ProteinPilot was used to search the UniProt database. The setup was as follows: instrument was TripleTOF 5600, cysteines were modified with iodoacetamide, and biological modification was selected as the ID focus. Peptides with an unused score greater than 1.3 (indicating a confidence level greater than 95%) were considered reliable, and proteins containing at least one unique peptide were accepted. After excluding common contaminant proteins such as keratins, antibodies, and serum albumins, the number of proteins identified in the CARMN-antisense and CARMN-sense samples was 147 and 212, respectively.

**Hematoxylin-Eosin (HE) staining**

The paraffin embedded tissue was cut into 6 μm thin slices and dried on the slide for 30 min. The slices were rinsed and dewaxed successively with xylene, ethanol and distilled water. The slices were dyed in hematoxylin dye (C0105M, Beyotime, China) for 10 minutes, and then the slices were dyed in eosin dye for 2 min after cleaning. Then, it was washed and dehydrated with gradient ethanol and treated with xylene in turn, and finally sealed for microscopic observation.

**Immunohistochemistry (IHC)**

The detailed experimental procedure has been described in our previous publication [7]. Briefly, xenograft tissues were paraffin-embedded and sectioned. The prepared slices were incubated with polyclonal rabbit anti-Ki67 antibody (A20018, dilution ratio 1:500; Abclonal, China) overnight at 4 °C and then with DAB (Zhongshan Biotech, Beijing, China) which stained the target protein in brown, and photographed under a microscope. ImageJ software with the IHC Profiler plug-in was used for immunohistochemical scoring [8]. The IHC Profiler takes the average gray value (staining intensity) and the percentage of positive area (staining area) of positive cells as IHC measurement indicators. The other antibodies used in the study were p-mTOR (67778-1-Ig, dilution ratio 1:500; Proteintech, USA), Nrf2 (AF300189, dilution ratio 1:100; AiFang biological, China), P62 (AF300544, dilution ratio 1:100; AiFang biological, China), and p-Akt (AF300996, dilution ratio 1:100; AiFang biological, China). QuickBlock™ Primary Antibody Dilution Buffer for Immunol Staining (P0262, Beyotime, China) was used to dilute antibody to working concentration.

**Western blot (WB)**

Cells were lysed using a detergent lysis buffer (50 mM Tris, pH 7.4; 150 mM NaCl; 1% NP-40; 0.5% sodium deoxycholate; 0.1% SDS; and 1% phosphatase inhibitor cocktail (Merck Millipore, Germany). WB was performed as previously described [9] using antibodies listed in Table S4. The levels of GAPDH and Lamin B1 were used as reference for cytoplasmic and nuclear proteins, respectively.

**Autophagic flux analysis**

After infecting HeLa cells with lentiviral vectors (CARMN overexpression and/or Baf-A1 treatment) for autophagic flux detection, the cells were subjected to drug selection and amplification, followed by observation under a confocal microscope to assess red, green, and yellow fluorescence signals. GFP, marked by green fluorescence, is more sensitive to pH and is completely quenched in autolysosomes. Its presence indicates a blockage in autolysosome formation. Activation of both red and green signals results in yellow fluorescence, suggesting a blockage in autolysosome formation and late-stage autophagy. If green fluorescence is quenched, only red fluorescence appears, indicating smooth progression of the autophagic process. A decrease in red fluorescence would suggest early-stage autophagy blockage.

**Reference**

[1] S. Wang, H. Zhu, B. Ding, X. Feng, W. Zhao, M. Cui, Y. Xu, M. Shi, J. Chen, H. Jin, Genetic variants in microRNAs are associated with cervical cancer risk, Mutagenesis, 34 (2019) 127-133.

[2] P. Langfelder, S. Horvath, WGCNA: an R package for weighted correlation network analysis, BMC Bioinformatics, 9 (2008) 559.

[3] Z. Ye, Y. Zhao, M. Chen, Q. Lu, J. Wang, X. Cui, H. Wang, P. Xue, Y. Jiang, Distribution and diagnostic value of single and multiple high-risk HPV infections in detection of cervical intraepithelial neoplasia: A retrospective multicenter study in China, J Med Virol, 96 (2024) e29835.

[4] J. Lee, D.J. Kim, H.J. Lee, Assessment of malignant potential for HPV types 16, 52, and 58 in the uterine cervix within a Korean cohort, Scientific reports, 14 (2024) 14619.

[5] S. Das, N. Wentzensen, G.F. Sawaya, D. Egemen, A. Locke, W. Kinney, T. Lorey, C.C. L, Primary human papillomavirus testing versus CO-testing: clinical outcomes in populations with different disease prevalence, J Natl Cancer Inst, (2024).

[6] X. Zhang, J. Cao, X. Li, Y. Zhang, W. Yan, B. Ding, J. Hu, H. Liu, X. Chen, Y. Nie, F. Liu, N. Lin, S. Wang, Comprehensive Analysis of the SUMO-related Signature: Implication for Diagnosis, Prognosis, and Immune Therapeutic Approaches in Cervical Cancer, Biochem Genet, (2024).

[7] S. Wang, S. Wu, H. Zhu, B. Ding, Y. Cai, J. Ni, Q. Wu, Q. Meng, X. Zhang, C. Zhang, X. Li, M. Wang, R. Chen, H. Jin, Z. Zhang, PSCA rs2294008 polymorphism contributes to the decreased risk for cervical cancer in a Chinese population, Scientific reports, 6 (2016) 23465.

[8] F. Varghese, A.B. Bukhari, R. Malhotra, A. De, IHC Profiler: an open source plugin for the quantitative evaluation and automated scoring of immunohistochemistry images of human tissue samples, PLoS One, 9 (2014) e96801.

[9] B. Gu, M. Li, Y. Zhang, L. Li, K. Yao, S. Wang, DR7 encoded by human herpesvirus 6 promotes glioma development and progression, Cancer Manag Res, 11 (2019) 2109-2118.

**Supplementary Tables**

Table S1. Characteristics of the study subjects for RNA-seq and qPCR validation

| **Experiment** | **Group** | **CC** | **CIN** | **NOR** |
| --- | --- | --- | --- | --- |
| RNA-seq | Number of samples | 10 | 10 | 10 |
|  | Median age (range, years) | 53 (50-70) | 45 (33-48) | 47 (44-59) |
| qPCR | Number of cervical squamous carcinoma | 23 |  | 20 |
|  | Median age (range, years) | 50 (32-66) |  | 50 (32-66) |
|  | Number of cervical adenocarcinoma | 20 |  | 20 |
|  | Median age (range, years) | 43 (22-63) |  | 43 (22-63) |

Abbreviations: CC, cervical cancer; CIN, cervical intrepithelial neplasia; NOR, normal control.

Table S2. Demographic characteristics and clinical features of the study subjects at different stages

| Variables^a^ | Stage I | |  | *P*^c^ | Stage II | |  | *P*^c^ |
| --- | --- | --- | --- | --- | --- | --- | --- | --- |
|  | Cases | Controls |  |  | Cases | Controls |  |  |
|  | n = 571 (%) | n = 657 (%) |  |  | n = 954 (%) | n = 1339 (%) |  |  |
| Age, year (mean ± SD) | 47.5 ± 10.1 | 47.3 ± 10.6 |  | 0.726 | 49.6 ± 11.0 | 49.5 ± 11.7 |  | 0.941 |
| Parity |  |  |  |  |  |  |  |  |
| 0-1 | 324 (58.3) | 446 (75.2) |  | < 0.001 | 486 (57.2) | 1019 (78.3) |  | < 0.001 |
| ≥ 2 | 232 (41.7) | 147 (24.8) |  |  | 364 (42.8) | 282 (21.7) |  |  |
| Abortion |  |  |  |  |  |  |  |  |
| No | 158 (29.5) | 154 (27.7) |  | 0.503 | 286 (32.6) | 409 (34.0) |  | 0.496 |
| Yes | 377 (70.5) | 402 (72.3) |  |  | 592 (67.4) | 794 (66.0) |  |  |
| Menopausal status |  |  |  |  |  |  |  |  |
| Premenopausal | 306 (56.0) | 476 (78.9) |  | < 0.001 | 497 (52.8) | 736 (59.4) |  | 0.002 |
| Postmenopausal | 240 (44.0) | 127 (21.1) |  |  | 444 (47.2) | 502 (40.6) |  |  |
| Histologic types |  |  |  |  |  |  |  |  |
| Squamous cell carcinoma | 538 (94.2) |  |  |  | 830 (91.5) |  |  |  |
| Adenocarcinomas | 24 (4.2) |  |  |  | 51 (5.6) |  |  |  |
| Adenosquamous carcinoma | 4 (0.7) |  |  |  | 16 (1.8) |  |  |  |
| Others^b^ | 5 (0.9) |  |  |  | 10 (1.1) |  |  |  |
| Depth of invasion |  |  |  |  |  |  |  |  |
| I | 383 (68) |  |  |  | 483 (55.3) |  |  |  |
| II | 145 (25.8) |  |  |  | 276 (31.6) |  |  |  |
| III | 28 (5.0) |  |  |  | 101 (11.6) |  |  |  |
| IV | 7 (1.2) |  |  |  | 13 (1.5) |  |  |  |
| ^a^ Some cases lack information of selected variables. | | | | | | | | |
| ^b^ Other histological types, such as cervical choriocarcinoma. | | | | | | | | |
| ^c^ χ^2^ test for the frequency distribution of demographic and clinical variables between the cases and controls. | | | | | | | | |

Table S3. Primer sequences used in qPCR and ChIP experiments

| **Gene** | **Experiment** | **Sequences（5’-3’）** |
| --- | --- | --- |
| CARMN-F | qPCR | GGCCTCGTTTG CATGATGAT |
| CARMN-R | qPCR | CCAGTCCTGGCTCCTGGAA |
| MAPK13-F | qPCR | CCACGTTAAACTGCCCATC |
| MAPK13-R | qPCR | CACCAGGTTTGTGGTAATTCG |
| EGF-F | qPCR | TGTGATTGCTTTCCTGGGTA |
| EGF-R | qPCR | TGGCTGAGCAGAGTTCCATA |
| FGFR3-F | qPCR | ACTGTCTGGGTCAAGGATGG |
| FGFR3-R | qPCR | GAGGATGGAGCGTCTGTCAC |
| MECOM -F | qPCR | CCAGTGGGACAAAGCTGACT |
| MECOM -R | qPCR | GAAAGGAGTGGGTCTTGCAT |
| PAK1-F | qPCR | CGTGGCTACATCTCCCATTT |
| PAK1-R | qPCR | GGATCGCCCACACTCACTAT |
| PPM1A -F | qPCR | GGGTAATGGGTTGCGATATG |
| PPM1A -R | qPCR | TGACCACGATTCAAGTCCAC |
| PRKCZ -F | qPCR | TACGCCATGAAAGTGGTGAA |
| PRKCZ -R | qPCR | TGACCAGGAACAACCGACTT |
| RASA2-F | qPCR | CCTACCACAAACAGCCAGGT |
| RASA2-R | qPCR | TTTGCCTGGACATAGAGTGG |
| TFAP2α-F | qPCR | TCCCCCCAAAATGGATCTGCT |
| TFAP2α-R | qPCR | GCAAATTGTATTAATATTCGATTTTGGGGAGG |
| LC3-F | qPCR | GAGCAGGAGAAAGACGAGGA |
| LC3-R | qPCR | CGGTAGAGGCAGCTCAGTTC |
| p62-F | qPCR | ACAGATGCCAGAATCCGAAG |
| p62-R | qPCR | CTGGGAGAGGGACTCAATCA |
| Bax-F | qPCR | TTTGCTTCAGGGTTTCATCC |
| Bax-R | qPCR | CAGTTGAAGTTGCCGTCAGA |
| Nrf2-F | qPCR | AGTCCAGAAGCCAAACTGACAGAAG |
| Nrf2-R | qPCR | GGAGAGGATGCTGCTGAAGGAATC |
| HO-1-F | qPCR | CCTCCCTGTACCACATCTATGT |
| HO-1-R | qPCR | GCTCTTCTGGGAAGTAGACAG |
| SOD -F | qPCR | ATCCTCTATCCAGAAAACACGG |
| SOD -R | qPCR | GCGTTTCCTGTCTTTGTACTTT |
| NQO1-F | qPCR | AAGCCGCAGACCTTGTGATATTCC |
| NQO1-R | qPCR | CATGGCAGCGTAAGTGTAAGCAAAC |
| Keap1-F | qPCR | ATTCAGCTGAGTGTTACTACCC |
| Keap1-R | qPCR | CAGCATAGATACAGTTGTGCAG |
| TIGAR -F | qPCR | ATTAGCAGCCAGTGTCTTAGTT |
| TIGAR -R | qPCR | GGAACACTTAAGGTCAGTCAGA |
| ERK-F | qPCR | ATCTCAACAAAGTTCGAGTTGC |
| ERK-R | qPCR | GTCTGAAGCGCAGTAAGATTTT |
| miR-143-F | qPCR | ACACTCCAGCTGGGGGTGCAGTGCTGCAT |
| miR-143-R | qPCR | TGGTGTCGTGGAGTCG |
| miR-145-F | qPCR | ACACTCCAGCTGGGGTCCAGTTTTCCCAGGA |
| miR-145-R | qPCR | TGGTGTCGTGGAGTCG |
| YBX1-F | qPCR | TGATGGAGGGTGCTGACAAC |
| YBX1-R | qPCR | CCTGCGGAATCGTGGTCTAT |
| DHX9-F | qPCR | TCCAACTGGAATCCTTGGAC |
| DHX9-R | qPCR | TTTTCCCACATCCAGTAGCC |
| HNRNPU-F | qPCR | CCCTTACAATGGAGAGTTTTGC |
| HNRNPU-R | qPCR | GACCAGCCAATACGAACTTCA |
| SYNCRIP-F | qPCR | ACTGTTGAATGGGCTGATCC |
| SYNCRIP-R | qPCR | CCTCCAAGTCTTTGCCATTC |
| KSRP-F | qPCR | ATCATCAACGACCTCCTCCA |
| KSRP-R | qPCR | CTTTCACATTCTCGCCACCT |
| U6-F | qPCR | CTCGCTTCGGCAGCACA |
| U6-R | qPCR | AACGCTTCACGAATTTGCGT |
| β-Actin-F | qPCR | ATCCGCAAAGACCTGT |
| β-Actin -R | qPCR | GGGTGTAACGCAACTAAG |
| GAPDH-F | qPCR | GTCAAGGCTGAGAACGGGAA |
| GAPDH-R | qPCR | AAATGAGCCCCAGCCTTCTC |
| CARMN-ChIP-F | ChIP | CCAGTCCTGGCTCCTGGAA |
| CARMN-ChIP-R | ChIP | GGCCTCGTTTGCATGATGAT |
| MAPK13-ChIP-F | ChIP | ATCAGATACCCCAGGGCAGG |
| MAPK13-ChIP-R | ChIP | ATTGCTCCTCAAAGGAAATGGC |

Table S4. Chemicals and antibodies used in this study

| **Chemical/antibody** | **Manufacturer** | **Catalog Number** |
| --- | --- | --- |
| Chloroquine | Selleck (USA) | S6999 |
| Bafilomycin A1 | Selleck (USA) | S1413 |
| Diphenyleneiodonium chloride | Selleck (USA) | S8639 |
| Tert-butylhydroquinone | Selleck (USA) | S4990 |
| MG-132 | Selleck (USA) | S2619 |
| Acetylcysteine (N-acetylcysteine) | Selleck (USA) | S1623 |
| SC79 | Selleck (USA) | S7863 |
| Honokiol | Selleck (USA) | S2310 |
| Cycloheximide | Selleck (USA) | S7418 |
| Dactinomycin | Selleck (USA) | S8964 |
| TFAP2α ASO | Ribobio (Guangzhou, China) |  |
| YBX1 ASO | Ribobio (Guangzhou, China) |  |
| DHX9 ASO | Ribobio (Guangzhou, China) |  |
| KSRP ASO | Ribobio (Guangzhou, China) |  |
| HNRNPU ASO | Ribobio (Guangzhou, China) |  |
| SYNCRIP ASO | Ribobio (Guangzhou, China) |  |
| CARMN ASO | Ribobio (Guangzhou, China) |  |
| miR-143-3p inhibitor | Ribobio (Guangzhou, China) |  |
| SP1 antibody | CST | 9389 |
| TFAP2α antibody | Proteintech | 13019-3-AP |
| LC3 I/II antibody | CST | 3868 |
| p62 antibody | CST | 23214 |
| Caspase-3 antibody | Abcam | ab32351 |
| Caspase-9 antibody | Abcam | ab32539 |
| Cyclin D1 antibody | ABclonal | A0310 |
| Cyclin B1 antibody | ABclonal | A2056 |
| Cyclin A1/A2 antibody | ABclonal | A2635 |
| GAPDH antibody | Proteintech | AC002 |
| Keap1 antibody | CST | 4678 |
| Nrf2 antibody | CST | 12721 |
| ERK5 antibody | Abcam | ab196609 |
| p-ERK5 antibody | Santa | sc-135760 |
| JNK antibody | CST | 9252 |
| p-JNK antibody | Abclonal | AP0631 |
| p-p38 antibody | CST | 4511 |
| p38 antibody | CST | 9212 |
| p-ERK1/2 antibody | CST | 9101 |
| ERK1/2 antibody | CST | 9194 |
| MAPK13 antibody | CST | 2308 |
| Lamin B1 antibody | CST | 17416 |
| GCLC antibody | CST | 48005 |
| HMOX1 antibody | Abclonal | A1346 |
| KSRP antibody | CST | 5398 |
| GPX4 antibody | CST | 59735 |
| DHX9 antibody | CST | 70998 |
| YBX1 antibody | Abclonal | A3534 |
| HA-Tag antibody | Abclonal | AE008 |
| Ki67 antibody | Abclonal | A20018 |
| p-mTOR antibody | Proteintech | 67778-1-Ig |
| p-Akt antibody | AiFang Biological | AF300996 |
| P62 antibody | AiFang Biological | AF300544 |
| Nrf2 antibody | AiFang Biological | AF300189 |

Abbreviations: ASO, antisense oligonucleotides; DL, dilution ratio.

Table S5. Plasmid and lentiviral vectors

| **Target** | **Purpose** | **Vector type** | **Vector** | **Bacterial Resistance** | **Manufacturer** |
| --- | --- | --- | --- | --- | --- |
| CARMN | OE | Lentivirus | GV502 | Puromycin | Genechem (Shanghai, China) |
| SP1 | KD | Lentivirus | GV248 | Puromycin | Genechem (Shanghai, China) |
| CARMN | OE | Plasmid | pcDNA3.1 | Ampicillin | Genechem (Shanghai, China) |
| TFAP2α | OE | Plasmid | pcDNA3.1 | Ampicillin | Genechem (Shanghai, China) |
| MAPK13 | OE | Plasmid | pcDNA3.1 | Ampicillin | Genechem (Shanghai, China) |
| YBX1 | Identifying binding sites | Truncated fragment plasmid | pcDNA3.1 | Ampicillin | Generay Biotech (Shanghai, China) |
| CARMN | Identifying binding sites | Truncated fragment plasmid | pcDNA3.1 | Ampicillin | Generay Biotech (Shanghai, China) |
| mRFP-GFP-LC3 | Monitoring autophagic flux | Lentivirus | GV540 | Puromycin | Genechem (Shanghai, China) |
| CARMN promoter region | Dual-luciferase reporter assay | Plasmid | pGL3-Basic | Ampicillin | Genechem (Shanghai, China) |
| MAPK13 promoter region | Dual-luciferase reporter assay | Plasmid | pGL3-Basic | Ampicillin | Genechem (Shanghai, China) |

Abbreviations: OE, overexpression; KD, knock down

Table S6. Top 20 of significantly up- and down-regulated lncRNAs/mRNAs in each comparison

| **Group comparison** | **lncRNA** | **mean_exp** | **Log_2_FC** | ***P*-value** | **FDR** |  | **mRNA** | **mean_exp** | **Log_2_FC** | ***P*-value** | **FDR** |
| --- | --- | --- | --- | --- | --- | --- | --- | --- | --- | --- | --- |
| CIN vs. NOR | AC006063.2 | 0.0503 | -7.2192 | 0.0007 | 0.0148 |  | CTSB | 30.4933 | -14.2951 | 0.0000 | 0.0000 |
|  | AC092162.2 | 0.1693 | -6.4561 | 0.0002 | 0.0067 |  | ZNF410 | 1.6818 | -12.5063 | 0.0000 | 0.0000 |
|  | AC007786.2 | 0.1730 | -5.3808 | 0.0029 | 0.0407 |  | DDR1 | 2.0464 | -11.9233 | 0.0000 | 0.0000 |
|  | FRMD6-AS2 | 0.4547 | -5.3619 | 0.0001 | 0.0045 |  | TRIM6-TRIM34 | 2.0990 | -11.0355 | 0.0000 | 0.0000 |
|  | AC011474.2 | 0.1207 | -5.2895 | 0.0022 | 0.0330 |  | PDGFRL | 4.7210 | -10.9432 | 0.0000 | 0.0000 |
|  | AC093390.1 | 0.0593 | -4.8244 | 0.0035 | 0.0452 |  | L3MBTL3 | 2.2263 | -10.8857 | 0.0000 | 0.0000 |
|  | MGC15885 | 0.1117 | -4.5988 | 0.0025 | 0.0368 |  | ATG101 | 8.7330 | -10.8842 | 0.0002 | 0.0046 |
|  | MIR1-1HG-AS1 | 0.1403 | -4.4478 | 0.0006 | 0.0127 |  | KCNAB1 | 1.4500 | -10.8757 | 0.0000 | 0.0000 |
|  | AP003548.1 | 0.4823 | -4.2084 | 0.0011 | 0.0210 |  | CIZ1 | 1.7007 | -10.7805 | 0.0000 | 0.0000 |
|  | PGM5-AS1 | 0.6343 | -4.0971 | 0.0001 | 0.0028 |  | NAPEPLD | 2.5173 | -10.7682 | 0.0000 | 0.0000 |
|  | AL354754.1 | 0.7450 | 10.8376 | 0.0000 | 0.0000 |  | LIPA | 3.7583 | 14.2054 | 0.0000 | 0.0000 |
|  | AL355076.2 | 0.2927 | 8.9278 | 0.0000 | 0.0000 |  | RPS24 | 14.8667 | 13.4348 | 0.0004 | 0.0095 |
|  | AC023796.2 | 0.2330 | 8.7142 | 0.0002 | 0.0055 |  | LASP1 | 6.3183 | 13.3511 | 0.0000 | 0.0000 |
|  | SMILR | 0.2237 | 8.6724 | 0.0020 | 0.0313 |  | SEC16A | 5.6187 | 13.1660 | 0.0000 | 0.0000 |
|  | AC003965.2 | 0.3607 | 8.6221 | 0.0006 | 0.0132 |  | RNF145 | 6.9073 | 13.1093 | 0.0000 | 0.0000 |
|  | LINC01629 | 0.4307 | 8.5622 | 0.0001 | 0.0029 |  | SLC25A1 | 8.7520 | 12.9676 | 0.0000 | 0.0000 |
|  | AC025176.1 | 0.3297 | 8.4998 | 0.0001 | 0.0041 |  | NR3C1 | 3.7460 | 12.9112 | 0.0000 | 0.0000 |
|  | AC080129.1 | 0.6417 | 8.4009 | 0.0000 | 0.0007 |  | C4orf3 | 7.1237 | 12.8900 | 0.0000 | 0.0000 |
|  | AL138902.1 | 0.2640 | 8.3174 | 0.0000 | 0.0001 |  | AGL | 3.1873 | 12.8378 | 0.0000 | 0.0000 |
|  | AC009262.1 | 0.2270 | 8.2854 | 0.0000 | 0.0015 |  | UCP2 | 4.1730 | 12.8084 | 0.0000 | 0.0000 |
| CC vs. CIN | LINC00261 | 1.3227 | -11.5915 | 0.0000 | 0.0000 |  | SCGB2A2 | 9.8357 | -14.1538 | 0.0000 | 0.0000 |
|  | AC011524.2 | 0.3837 | -9.9396 | 0.0000 | 0.0000 |  | FOLR1 | 3.9107 | -13.9963 | 0.0000 | 0.0000 |
|  | LINC01594 | 0.3330 | -9.4009 | 0.0000 | 0.0000 |  | BPIFB1 | 102.2857 | -12.8999 | 0.0000 | 0.0000 |
|  | AC084866.1 | 3.7097 | -9.2710 | 0.0000 | 0.0000 |  | EEF1D | 21.5193 | -12.7125 | 0.0000 | 0.0000 |
|  | LHX1-DT | 0.2747 | -9.2456 | 0.0000 | 0.0000 |  | ENPP3 | 1.9587 | -12.4556 | 0.0000 | 0.0000 |
|  | BARX1-DT | 0.1897 | 9.1472 | 0.0000 | 0.0000 |  | ADCYAP1R1 | 1.1537 | -12.4260 | 0.0000 | 0.0000 |
|  | AC109462.1 | 0.2210 | -9.1344 | 0.0000 | 0.0001 |  | ANK2 | 1.7800 | -12.2568 | 0.0000 | 0.0000 |
|  | AC079160.1 | 0.1883 | 9.1241 | 0.0000 | 0.0000 |  | FBLN2 | 13.3703 | -12.2255 | 0.0000 | 0.0000 |
|  | AC011524.1 | 0.2660 | -9.0224 | 0.0000 | 0.0005 |  | CDH12 | 1.7960 | -12.0967 | 0.0000 | 0.0000 |
|  | LINC00906 | 1.3123 | -8.8486 | 0.0000 | 0.0000 |  | LMBRD1 | 5.3833 | -12.0924 | 0.0000 | 0.0000 |
|  | BARX1-DT | 0.1897 | 9.1472 | 0.0000 | 0.0000 |  | NPM1 | 7.7140 | 14.1121 | 0.0000 | 0.0000 |
|  | AC079160.1 | 0.1883 | 9.1241 | 0.0000 | 0.0000 |  | PLK2 | 8.3887 | 13.0081 | 0.0000 | 0.0000 |
|  | HOXC-AS3 | 0.1350 | 8.4717 | 0.0005 | 0.0091 |  | SPG11 | 5.1537 | 12.9764 | 0.0000 | 0.0000 |
|  | LINC01526 | 0.1157 | 8.4305 | 0.0000 | 0.0000 |  | CPS1 | 2.7053 | 12.9624 | 0.0000 | 0.0000 |
|  | AC034223.2 | 0.1130 | 8.3966 | 0.0025 | 0.0304 |  | HACD3 | 8.9023 | 12.5338 | 0.0000 | 0.0000 |
|  | AC002076.1 | 0.0843 | 7.9715 | 0.0014 | 0.0199 |  | CALB1 | 1.7770 | 12.3740 | 0.0000 | 0.0000 |
|  | AC022031.1 | 0.0547 | 7.1898 | 0.0028 | 0.0333 |  | GRAMD1B | 1.3177 | 11.7406 | 0.0000 | 0.0000 |
|  | AC116049.2 | 0.0457 | 7.0768 | 0.0035 | 0.0393 |  | RAB40C | 1.8787 | 11.5541 | 0.0000 | 0.0000 |
|  | HOXC-AS2 | 0.0770 | 6.9658 | 0.0041 | 0.0441 |  | MRPL42 | 1.9667 | 11.4019 | 0.0000 | 0.0000 |
|  | LINC02154 | 5.5220 | 6.9644 | 0.0000 | 0.0000 |  | FGF11 | 1.3793 | 11.1555 | 0.0000 | 0.0000 |
| CC vs. NOR | LINC00906 | 1.3123 | -11.7628 | 0.0000 | 0.0000 |  | SCGB2A2 | 9.8357 | -14.8337 | 0.0025 | 0.0174 |
|  | PGM5-AS1 | 0.6343 | -10.8114 | 0.0000 | 0.0000 |  | PI16 | 5.6467 | -14.0125 | 0.0000 | 0.0000 |
|  | AC092691.1 | 0.6447 | -10.7507 | 0.0000 | 0.0005 |  | PENK | 6.7243 | -13.9706 | 0.0000 | 0.0000 |
|  | AL021408.1 | 0.5193 | -10.3826 | 0.0000 | 0.0000 |  | LMBRD1 | 5.3833 | -13.0118 | 0.0000 | 0.0000 |
|  | AC055874.1 | 0.3557 | -9.9054 | 0.0014 | 0.0099 |  | CADM3 | 6.2393 | -13.0007 | 0.0000 | 0.0000 |
|  | LINC02106 | 0.3057 | -9.8376 | 0.0084 | 0.0403 |  | TMOD1 | 9.4567 | -12.9851 | 0.0000 | 0.0000 |
|  | LINC01532 | 0.3753 | -9.7912 | 0.0000 | 0.0000 |  | GJA1 | 17.1790 | -12.7139 | 0.0000 | 0.0000 |
|  | LINC00261 | 1.3227 | -9.7830 | 0.0000 | 0.0000 |  | FBLN2 | 13.3703 | -12.6481 | 0.0000 | 0.0000 |
|  | EPHA5-AS1 | 0.2847 | -9.6257 | 0.0000 | 0.0006 |  | ADH1B | 2.1447 | -12.6207 | 0.0000 | 0.0000 |
|  | AC104794.4 | 0.2977 | -9.5868 | 0.0000 | 0.0000 |  | ABI3BP | 4.9020 | -12.5906 | 0.0000 | 0.0000 |
|  | AC005993.1 | 0.6680 | 10.8065 | 0.0000 | 0.0000 |  | LIPA | 3.7583 | 15.3274 | 0.0000 | 0.0000 |
|  | AC080129.1 | 0.6417 | 10.6312 | 0.0000 | 0.0000 |  | SHISA5 | 13.0600 | 15.0103 | 0.0000 | 0.0000 |
|  | LINC01833 | 0.5143 | 10.5245 | 0.0000 | 0.0000 |  | ADA2 | 7.2677 | 14.3965 | 0.0000 | 0.0000 |
|  | AC022031.2 | 0.4470 | 10.3476 | 0.0011 | 0.0084 |  | NPM1 | 7.7140 | 14.1121 | 0.0000 | 0.0000 |
|  | AL117329.1 | 0.5250 | 10.0000 | 0.0000 | 0.0000 |  | ANXA3 | 12.9073 | 14.0535 | 0.0000 | 0.0000 |
|  | FEZF1-AS1 | 0.3753 | 9.8626 | 0.0000 | 0.0000 |  | BZW1 | 6.0043 | 14.0416 | 0.0000 | 0.0000 |
|  | LINC01629 | 0.4307 | 9.8345 | 0.0000 | 0.0000 |  | INHBA | 7.3123 | 13.9946 | 0.0000 | 0.0000 |
|  | HOXC13-AS | 0.3060 | 9.6970 | 0.0000 | 0.0000 |  | DHCR24 | 20.0883 | 13.7565 | 0.0000 | 0.0000 |
|  | AL121790.2 | 0.3517 | 9.6294 | 0.0001 | 0.0009 |  | RNF145 | 6.9073 | 13.5367 | 0.0000 | 0.0000 |
|  | AC003965.2 | 0.3607 | 9.4242 | 0.0000 | 0.0000 |  | COMMD4 | 5.0967 | 13.3505 | 0.0000 | 0.0000 |

Table S7. Comparison of model performance

| Model | Traindata (75%) | |  | Testdata (25%) | |
| --- | --- | --- | --- | --- | --- |
|  | AUC of ROC | AUC of PR |  | AUC of ROC | AUC of PR |
| DT | 0.964 | 0.915 |  | 0.872 | 0.713 |
| RF | 0.896 | 0.769 |  | 0.857 | 0.679 |
| XGBOOST | 0.985 | 0.961 |  | 0.963 | 0.917 |
| ENR | 0.811 | 0.585 |  | 0.818 | 0.596 |
| SVM | 0.940 | 0.847 |  | 0.927 | 0.817 |
| MLP | 0.992 | 0.979 |  | 0.905 | 0.771 |
| Lightgbm | 1.000 | 1.000 |  | 0.987 | 0.966 |
| KNN | 1.000 | 0.999 |  | 0.968 | 0.929 |
| LR | 0.857 | 0.664 |  | 0.868 | 0.699 |
| Stacking | 0.979 | 0.943 |  | 0.963 | 0.908 |

Table S8. Top 30 differentially expressed genes in CARMN overexpressing HeLa cells

| **Symbol** | **Log_2_FC** | ***P*-value** | **FDR** |
| --- | --- | --- | --- |
| NCOA5 | -10.6499 | 0.0000 | 0.0000 |
| MAGEA2B | 10.3294 | 0.0000 | 0.0000 |
| ARFGEF3 | -3.9696 | 0.0003 | 0.0279 |
| ANKRD1 | 3.4180 | 0.0000 | 0.0002 |
| IL7R | 3.3788 | 0.0000 | 0.0000 |
| NAV3 | 3.3219 | 0.0002 | 0.0187 |
| NDUFA4L2 | -3.0287 | 0.0000 | 0.0004 |
| PLEKHG1 | -2.9758 | 0.0001 | 0.0153 |
| CTSS | 2.9615 | 0.0005 | 0.0406 |
| CA9 | -2.9224 | 0.0000 | 0.0000 |
| NCF2 | 2.8331 | 0.0000 | 0.0000 |
| SH3TC2 | 2.6393 | 0.0000 | 0.0000 |
| PRICKLE2 | 2.5615 | 0.0000 | 0.0000 |
| ENC1 | 2.4894 | 0.0002 | 0.0189 |
| RNF11 | -2.4214 | 0.0000 | 0.0000 |
| IL6 | 2.3692 | 0.0000 | 0.0042 |
| PDCD1LG2 | 2.2311 | 0.0004 | 0.0323 |
| CD180 | 2.2224 | 0.0000 | 0.0000 |
| GALNTL6 | -2.2224 | 0.0005 | 0.0379 |
| CCL5 | 2.1489 | 0.0000 | 0.0000 |
| ACE2 | 2.1240 | 0.0003 | 0.0253 |
| RNASE4 | -2.0849 | 0.0003 | 0.0279 |
| BMF | -2.0534 | 0.0000 | 0.0004 |
| DDX58 | 2.0209 | 0.0000 | 0.0000 |
| SLC2A4 | -1.9969 | 0.0000 | 0.0011 |
| TGM2 | 1.9877 | 0.0001 | 0.0144 |
| ARSB | -1.9820 | 0.0001 | 0.0090 |
| MYEOV | 1.9712 | 0.0001 | 0.0101 |
| SERPINE2 | 1.9675 | 0.0000 | 0.0000 |
| PPFIA4 | -1.9364 | 0.0001 | 0.0075 |

Table S9. Prediction of TFs binding to the promoter region of MAPK13 by PROMO

| **TFs** | **Matrix_width** |
| --- | --- |
| XBP-1 | 6 |
| YY1 | 4 |
| C/EBP-β | 4 |
| GR-β | 5 |
| TFII-I | 6 |
| IRF-2 | 6 |
| PAX-5 | 7 |
| p53 | 7 |
| TCF-4E | 7 |
| GR-α | 5 |
| NF-1 | 8 |
| HNF-3α | 8 |
| GR | 7 |
| TFIID | 7 |
| AP-2α | 6 |
| RXR-α | 7 |
| c-Jun | 7 |
| c-Fos | 10 |
| GCF | 9 |
| FOXP3 | 6 |
| c-Ets-1 | 7 |
| ER-α | 5 |

Table S10. Prediction of TFs binding to the promoter region of MAPK13 by Alibaba 2.0

| **TFs** | **Predicted binding numbers** |
| --- | --- |
| SP1 | 76 |
| C/EBP-α | 16 |
| AP-2α | 10 |
| AP-1 | 9 |
| GR | 6 |
| GATA-1 | 5 |
| NF-κB | 4 |
| C/EBP-δ | 3 |
| c-Jun | 3 |
| C/EBP-β | 2 |
| ER | 2 |
| C/EBP-γ | 1 |
| c-Fos | 1 |
| YY1 | 1 |
| IRF-1 | 1 |
| c-Ets-1 | 1 |

Table S11. The predicted TF binding sites of the MAPK13 promoter from JASPAR

| **Matrix ID** | **Name** | **Score** | **Relative score** | **Start** | **End** | **Strand** | **Predicted sequence** |
| --- | --- | --- | --- | --- | --- | --- | --- |
| MAPK13_p | TFAP2A | 10.374 | 0.999 | 1937 | 1945 | + | GCCCGGGGC |
| MAPK13_p | TFAP2A | 10.282 | 0.996 | 1937 | 1945 | - | GCCCCGGGC |
| MAPK13_p | TFAP2A | 9.783 | 0.98 | 254 | 262 | - | GCCGGGGGC |
| MAPK13_p | TFAP2A | 9.711 | 0.978 | 254 | 262 | + | GCCCCCGGC |
| MAPK13_p | TFAP2A | 9.514 | 0.971 | 183 | 191 | + | GCCTGAGGC |
| MAPK13_p | TFAP2A | 9.421 | 0.968 | 183 | 191 | - | GCCTCAGGC |
| MAPK13_p | TFAP2A | 9.269 | 0.963 | 211 | 219 | - | GCCCCAAGC |

Table S12. Potential Targets of TFAP2α Based on the ChIPBase Database in HeLa-S3 Cell Line

| A1BG | CASP3 | DPH5 | HAP1 | MCM3AP | PEMT | RPS23 | TBC1D17 | ZBTB37 |
| --- | --- | --- | --- | --- | --- | --- | --- | --- |
| A2M | CASP4 | DPH6 | HAPLN3 | MCM4 | PEPD | RPS25 | TBC1D23 | ZBTB4 |
| A3GALT2 | CASP5 | DPH7 | HARBI1 | MCM6 | PER1 | RPS27 | TBC1D24 | ZBTB40 |
| A4GALT | CASP6 | DPM1 | HARS1 | MCM7 | PER3 | RPS27L | TBC1D25 | ZBTB41 |
| AAAS | CASP7 | DPM2 | HAS1 | MCM8 | PERM1 | RPS29 | TBC1D2B | ZBTB42 |
| AACS | CASP8AP2 | DPM3 | HAS2 | MCM9 | PERP | RPS3A | TBC1D30 | ZBTB43 |
| AADAT | CASP9 | DPP3 | HASPIN | MCMBP | PES1 | RPS4X | TBC1D31 | ZBTB45 |
| AAGAB | CASQ2 | DPP7 | HAT1 | MCOLN1 | PET117 | RPS5 | TBC1D32 | ZBTB47 |
| AAMDC | CAST | DPP8 | HAUS1 | MCRIP1 | PEX10 | RPS6 | TBC1D3F | ZBTB48 |
| AAMP | CASTOR1 | DPP9 | HAUS2 | MCRIP2 | PEX11A | RPS6KA1 | TBC1D5 | ZBTB5 |
| AANAT | CASZ1 | DPY19L1 | HAUS3 | MCRS1 | PEX11B | RPS6KA4 | TBC1D7 | ZBTB6 |
| AAR2 | CAT | DPY19L3 | HAUS4 | MCTP1 | PEX11G | RPS6KA5 | TBC1D8 | ZBTB7A |
| AARS1 | CATSPER1 | DPY19L4 | HAUS6 | MCU | PEX12 | RPS6KB1 | TBC1D9 | ZBTB7B |
| AARS2 | CATSPERG | DPY30 | HAUS7 | MCUB | PEX13 | RPS6KB2 | TBC1D9B | ZBTB8A |
| AARSD1 | CAV1 | DPYD | HAUS8 | MCUR1 | PEX16 | RPS6KC1 | TBCA | ZBTB8OS |
| AASDH | CAVIN1 | DQX1 | HAVCR2 | MDC1 | PEX19 | RPS7 | TBCB | ZC2HC1A |
| AATF | CAVIN2 | DR1 | HAX1 | MDGA1 | PEX2 | RPS8 | TBCC | ZC2HC1C |
| AATK | CAVIN3 | DRAM1 | HBE1 | MDH1 | PEX26 | RPSA | TBCCD1 | ZC3H10 |
| ABCA12 | CBARP | DRAM2 | HBG2 | MDH1B | PEX3 | RPTN | TBCE | ZC3H12A |
| ABCA2 | CBFA2T2 | DRC3 | HBP1 | MDH2 | PEX5 | RPTOR | TBCEL | ZC3H12C |
| ABCA3 | CBFB | DRG1 | HBS1L | MDK | PEX6 | RPUSD1 | TBK1 | ZC3H14 |
| ABCA5 | CBL | DRG2 | HCAR1 | MDM1 | PFAS | RPUSD3 | TBL1X | ZC3H15 |
| ABCA7 | CBLB | DROSHA | HCCS | MDM2 | PFDN1 | RRAD | TBL1XR1 | ZC3H18 |
| ABCB10 | CBLL1 | DSCAML1 | HCFC1 | MDM4 | PFDN4 | RRAGA | TBL2 | ZC3H3 |
| ABCB11 | CBLN3 | DSCC1 | HCFC1R1 | MDN1 | PFKFB1 | RRAGB | TBL3 | ZC3H4 |
| ABCB6 | CBR3 | DSEL | HCFC2 | MDP1 | PFKFB2 | RRAGC | TBP | ZC3H7A |
| ABCB7 | CBR4 | DSG2 | HCN3 | ME2 | PFKFB3 | RRAS | TBPL1 | ZC3HAV1 |
| ABCB8 | CBWD5 | DSN1 | HCN4 | ME3 | PFKFB4 | RRAS2 | TBPL2 | ZC3HC1 |
| ABCB9 | CBX1 | DSP | HDAC1 | MEA1 | PFKL | RREB1 | TBX10 | ZCCHC10 |
| ABCC10 | CBX2 | DSTN | HDAC10 | MEAF6 | PFN1 | RRM1 | TBX2 | ZCCHC14 |
| ABCC2 | CBX3 | DSTYK | HDAC11 | MEAK7 | PFN3 | RRM2 | TBX3 | ZCCHC17 |
| ABCC3 | CBX4 | DTL | HDAC2 | MECOM | PFN4 | RRM2B | TBX6 | ZCCHC24 |
| ABCC4 | CBX5 | DTNB | HDAC3 | MECR | PGAM2 | RRN3 | TBXA2R | ZCCHC3 |
| ABCC5 | CBX6 | DTWD1 | HDAC4 | MED1 | PGAM5 | RRP1 | TC2N | ZCCHC4 |
| ABCD3 | CBX7 | DTWD2 | HDAC5 | MED10 | PGAP1 | RRP12 | TCAF1 | ZCCHC8 |
| ABCD4 | CBX8 | DTX3L | HDAC6 | MED11 | PGAP2 | RRP8 | TCAIM | ZCRB1 |
| ABCE1 | CBY1 | DTYMK | HDAC7 | MED12 | PGAP4 | RRP9 | TCEA1 | ZCWPW1 |
| ABCF1 | CBY3 | DUOX2 | HDDC3 | MED13 | PGAP6 | RRS1 | TCEA2 | ZCWPW2 |
| ABCF2 | CC2D1A | DUS1L | HDGF | MED14 | PGBD1 | RS1 | TCEA3 | ZDBF2 |
| ABCF2-H2BK1 | CC2D1B | DUS2 | HDGFL2 | MED16 | PGBD2 | RSAD1 | TCEANC | ZDHHC1 |
| ABCF3 | CC2D2B | DUS3L | HDHD2 | MED17 | PGBD4 | RSBN1 | TCEANC2 | ZDHHC11B |
| ABHD10 | CCAR1 | DUS4L | HDHD3 | MED18 | PGC | RSBN1L | TCERG1L | ZDHHC12 |
| ABHD11 | CCAR2 | DUS4L-BCAP29 | HDHD5 | MED19 | PGD | RSF1 | TCF20 | ZDHHC13 |
| ABHD12 | CCBE1 | DUSP1 | HEATR1 | MED20 | PGF | RSL1D1 | TCF25 | ZDHHC14 |
| ABHD13 | CCDC102A | DUSP10 | HEATR4 | MED22 | PGGT1B | RSL24D1 | TCF3 | ZDHHC16 |
| ABHD14A | CCDC103 | DUSP11 | HEATR5A | MED23 | PGK1 | RSPH1 | TCF7L2 | ZDHHC17 |
| ABHD14A-ACY1 | CCDC107 | DUSP13 | HEATR6 | MED24 | PGLS | RSPH3 | TCFL5 | ZDHHC18 |
| ABHD14B | CCDC113 | DUSP14 | HEATR9 | MED26 | PGLYRP1 | RSPRY1 | TCHP | ZDHHC2 |
| ABHD15 | CCDC115 | DUSP15 | HEBP1 | MED27 | PGLYRP4 | RSRC2 | TCIRG1 | ZDHHC20 |
| ABHD16A | CCDC117 | DUSP16 | HEBP2 | MED28 | PGM1 | RSRP1 | TCL1A | ZDHHC23 |
| ABHD17A | CCDC121 | DUSP2 | HECA | MED29 | PGM2L1 | RTBDN | TCN1 | ZDHHC24 |
| ABHD17B | CCDC122 | DUSP22 | HECTD1 | MED30 | PGM3 | RTCA | TCOF1 | ZDHHC3 |
| ABHD18 | CCDC124 | DUSP28 | HECTD2 | MED31 | PGP | RTEL1 | TCP1 | ZDHHC4 |
| ABHD2 | CCDC125 | DUSP3 | HECTD3 | MED4 | PGPEP1 | RTEL1-TNFRSF6B | TCP11L1 | ZDHHC5 |
| ABHD3 | CCDC127 | DUSP4 | HECTD4 | MED7 | PGPEP1L | RTF1 | TCP11L2 | ZDHHC6 |
| ABHD5 | CCDC134 | DUSP5 | HECW2 | MED9 | PGRMC2 | RTF2 | TCTA | ZDHHC7 |
| ABHD6 | CCDC14 | DUSP6 | HEG1 | MEF2A | PGS1 | RTKN | TCTE1 | ZDHHC9 |
| ABHD8 | CCDC142 | DUSP7 | HELB | MEF2B | PHACTR4 | RTL10 | TCTN1 | ZEB1 |
| ABI2 | CCDC146 | DUSP8 | HELLS | MEF2D | PHAX | RTL6 | TCTN3 | ZER1 |
| ABITRAM | CCDC150 | DUT | HELQ | MEFV | PHB1 | RTN2 | TDG | ZFAND1 |
| ABL1 | CCDC153 | DVL1 | HELZ | MEGF9 | PHB2 | RTN3 | TDP1 | ZFAND2A |
| ABLIM1 | CCDC157 | DVL2 | HELZ2 | MEIG1 | PHC2 | RTN4IP1 | TDP2 | ZFAND2B |
| ABLIM3 | CCDC158 | DXO | HEMK1 | MEIS1 | PHC3 | RTN4RL1 | TDRD3 | ZFAND3 |
| ABR | CCDC159 | DYM | HENMT1 | MEIS2 | PHETA1 | RTP2 | TDRD7 | ZFAND4 |
| ABRACL | CCDC166 | DYNC1H1 | HEPACAM2 | MEIS3 | PHF1 | RTRAF | TDRKH | ZFAND5 |
| ABRAXAS1 | CCDC167 | DYNC1I2 | HERC1 | MELK | PHF10 | RTTN | TEAD1 | ZFAND6 |
| ABRAXAS2 | CCDC168 | DYNC1LI1 | HERC3 | MELTF | PHF13 | RUBCN | TEAD2 | ZFAT |
| ABT1 | CCDC169 | DYNC2I1 | HERC5 | MEMO1 | PHF14 | RUFY2 | TEAD3 | ZFC3H1 |
| ABTB1 | CCDC169-SOHLH2 | DYNC2I2 | HERC6 | MEN1 | PHF19 | RUFY3 | TEAD4 | ZFHX2 |
| ABTB2 | CCDC171 | DYNLL1 | HERPUD1 | MEOX1 | PHF20 | RUNDC1 | TEC | ZFHX3 |
| ACAA1 | CCDC177 | DYNLL2 | HERPUD2 | MESD | PHF20L1 | RUNX1 | TECPR1 | ZFP1 |
| ACAA2 | CCDC18 | DYNLRB2 | HES1 | MESP1 | PHF21A | RUNX3 | TECPR2 | ZFP14 |
| ACACA | CCDC187 | DYNLT1 | HES2 | MEST | PHF23 | RUSC2 | TECR | ZFP2 |
| ACAD11 | CCDC188 | DYNLT2 | HES3 | MET | PHF3 | RUSF1 | TEDC1 | ZFP30 |
| ACAD8 | CCDC191 | DYNLT2B | HES4 | METAP1 | PHF5A | RUVBL1 | TEF | ZFP36L1 |
| ACAD9 | CCDC194 | DYNLT3 | HES5 | METAP1D | PHF6 | RUVBL2 | TEN1 | ZFP36L2 |
| ACADM | CCDC197 | DYNLT4 | HES6 | METAP2 | PHF8 | RWDD2A | TEN1-CDK3 | ZFP37 |
| ACADS | CCDC200 | DYRK1A | HES7 | METRN | PHGDH | RWDD2B | TENT2 | ZFP62 |
| ACADSB | CCDC201 | DYRK1B | HESX1 | METTL1 | PHIP | RWDD3 | TENT4A | ZFP64 |
| ACAP2 | CCDC24 | DYRK3 | HEXA | METTL13 | PHKA1 | RWDD4 | TENT4B | ZFP69B |
| ACAP3 | CCDC25 | DYSF | HEXD | METTL15 | PHKA2 | RXFP4 | TENT5A | ZFP82 |
| ACAT2 | CCDC28A | DZANK1 | HEXIM1 | METTL17 | PHKB | RXRB | TENT5C | ZFP91 |
| ACBD3 | CCDC30 | DZIP1L | HEXIM2 | METTL18 | PHKG1 | RXYLT1 | TEP1 | ZFP91-CNTF |
| ACBD5 | CCDC32 | DZIP3 | HEY1 | METTL21A | PHKG2 | RYBP | TEPSIN | ZFPL1 |
| ACBD6 | CCDC34 | E2F1 | HEY2 | METTL22 | PHLDA1 | RYK | TERB1 | ZFPM1 |
| ACBD7 | CCDC38 | E2F2 | HEYL | METTL23 | PHLDA2 | S100A1 | TERF1 | ZFR |
| ACCS | CCDC40 | E2F4 | HGH1 | METTL25 | PHLDA3 | S100A10 | TERF2 | ZFTA |
| ACD | CCDC43 | E2F5 | HGS | METTL26 | PHLDB3 | S100A11 | TERF2IP | ZFX |
| ACE2 | CCDC47 | E2F7 | HGSNAT | METTL27 | PHLPP1 | S100A12 | TES | ZFYVE1 |
| ACER1 | CCDC50 | E4F1 | HHEX | METTL2A | PHOSPHO1 | S100A13 | TESK1 | ZFYVE16 |
| ACER2 | CCDC51 | EAF1 | HHIPL2 | METTL3 | PHOSPHO2 | S100A14 | TESMIN | ZFYVE19 |
| ACER3 | CCDC57 | EAF2 | HIBADH | METTL4 | PHOX2A | S100A16 | TEX10 | ZFYVE21 |
| ACHE | CCDC59 | EAPP | HIBCH | METTL5 | PHOX2B | S100A2 | TEX13B | ZFYVE27 |
| ACIN1 | CCDC61 | EBAG9 | HID1 | METTL6 | PHPT1 | S100A3 | TEX2 | ZFYVE9 |
| ACLY | CCDC66 | EBLN1 | HIF1A | METTL8 | PHTF1 | S100A4 | TEX22 | ZGLP1 |
| ACO1 | CCDC68 | EBNA1BP2 | HIF1AN | METTL9 | PHTF2 | S100A5 | TEX261 | ZGPAT |
| ACO2 | CCDC69 | EBP | HIGD1A | MEX3A | PHYH | S100A6 | TEX264 | ZGRF1 |
| ACOT1 | CCDC7 | EBPL | HIGD2A | MEX3B | PHYHIP | S100A8 | TEX30 | ZHX1 |
| ACOT12 | CCDC71 | ECD | HIGD2B | MEX3C | PHYKPL | S100P | TEX33 | ZHX1-C8orf76 |
| ACOT13 | CCDC71L | ECE1 | HIKESHI | MFAP1 | PI3 | S100PBP | TEX36 | ZHX2 |
| ACOT2 | CCDC77 | ECEL1 | HILPDA | MFAP2 | PI4K2A | S1PR5 | TEX46 | ZHX3 |
| ACOT7 | CCDC78 | ECH1 | HINT1 | MFAP3L | PI4KA | SAAL1 | TEX48 | ZIC5 |
| ACOT8 | CCDC82 | ECHDC1 | HINT2 | MFAP4 | PI4KB | SAC3D1 | TEX52 | ZKSCAN2 |
| ACOT9 | CCDC83 | ECHDC3 | HINT3 | MFAP5 | PIANP | SACM1L | TEX53 | ZKSCAN3 |
| ACOX1 | CCDC85B | ECHS1 | HIP1 | MFHAS1 | PIAS1 | SAE1 | TEX54 | ZKSCAN4 |
| ACP2 | CCDC85C | ECI1 | HIP1R | MFN1 | PIAS2 | SAFB | TEX9 | ZKSCAN8 |
| ACP5 | CCDC86 | ECPAS | HIPK1 | MFN2 | PIAS3 | SAFB2 | TFAM | ZKSCAN8P1 |
| ACP6 | CCDC87 | ECSCR | HIPK3 | MFNG | PIAS4 | SAGSIN1 | TFAP2A | ZMAT1 |
| ACSBG1 | CCDC88A | ECSIT | HIPK4 | MFRP | PICALM | SALL4 | TFAP2C | ZMAT3 |
| ACSF3 | CCDC88C | ECT2 | HIRA | MFSD1 | PIDD1 | SAMD1 | TFAP4 | ZMAT5 |
| ACSL1 | CCDC9 | EDAR | HIRIP3 | MFSD10 | PIERCE1 | SAMD10 | TFB1M | ZMIZ2 |
| ACSL3 | CCDC90B | EDC3 | HIVEP2 | MFSD12 | PIERCE2 | SAMD13 | TFB2M | ZMPSTE24 |
| ACSL4 | CCDC91 | EDC4 | HIVEP3 | MFSD13A | PIEZO1 | SAMD14 | TFCP2 | ZMYM1 |
| ACSS2 | CCDC92 | EDEM1 | HJV | MFSD14A | PIF1 | SAMD4B | TFDP1 | ZMYM2 |
| ACTB | CCDC93 | EDEM3 | HK3 | MFSD14B | PIGA | SAMD8 | TFE3 | ZMYM3 |
| ACTBL2 | CCDC96 | EDF1 | HLA-A | MFSD3 | PIGB | SAMD9 | TFEB | ZMYM5 |
| ACTC1 | CCDC9B | EDIL3 | HLA-B | MFSD4B | PIGBOS1 | SAMHD1 | TFEC | ZMYM6 |
| ACTG1 | CCER2 | EDN2 | HLA-DMA | MFSD5 | PIGC | SAMM50 | TFF1 | ZMYND10 |
| ACTL10 | CCHCR1 | EDRF1 | HLA-DMB | MFSD8 | PIGF | SANBR | TFF2 | ZMYND12 |
| ACTL6A | CCL19 | EEA1 | HLA-DOA | MGARP | PIGG | SAP25 | TFF3 | ZMYND19 |
| ACTL6B | CCL2 | EED | HLA-DOB | MGAT1 | PIGH | SAP30 | TFG | ZMYND8 |
| ACTN3 | CCL21 | EEF1A1 | HLA-E | MGAT2 | PIGK | SAP30BP | TFPI | ZNF10 |
| ACTN4 | CCL24 | EEF1A2 | HLCS | MGAT4B | PIGL | SAP30L | TFPI2 | ZNF100 |
| ACTR10 | CCL28 | EEF1AKMT1 | HLTF | MGAT4D | PIGM | SAPCD2 | TFPT | ZNF101 |
| ACTR1A | CCL5 | EEF1AKMT2 | HM13 | MGAT5 | PIGN | SAR1A | TFRC | ZNF117 |
| ACTR1B | CCN1 | EEF1AKMT4 | HMBOX1 | MGAT5B | PIGO | SAR1B | TGDS | ZNF12 |
| ACTR2 | CCN2 | EEF1AKMT4-ECE2 | HMBS | MGLL | PIGP | SARAF | TGFA | ZNF131 |
| ACTR3B | CCN3 | EEF1B2 | HMCES | MGME1 | PIGQ | SARM1 | TGFB1I1 | ZNF132 |
| ACTR5 | CCN5 | EEF1D | HMCN1 | MGMT | PIGS | SARNP | TGFB2 | ZNF14 |
| ACTR6 | CCNA2 | EEF1E1 | HMG20B | MGRN1 | PIGT | SARS1 | TGFBR1 | ZNF140 |
| ACTRT3 | CCNB1IP1 | EEF1E1-BLOC1S5 | HMGA1 | MGST2 | PIGU | SARS2 | TGFBR2 | ZNF142 |
| ACVR2B | CCNB2 | EEF1G | HMGA2 | MGST3 | PIGW | SART1 | TGFBR3 | ZNF143 |
| ACY1 | CCNC | EEF2 | HMGB2 | MIB1 | PIGX | SART3 | TGFBRAP1 | ZNF146 |
| ACY3 | CCND1 | EEF2K | HMGB3 | MIB2 | PIGY | SASH1 | TGIF2 | ZNF148 |
| ACYP1 | CCNDBP1 | EEFSEC | HMGCL | MICA | PIGZ | SASS6 | TGIF2-RAB5IF | ZNF155 |
| ACYP2 | CCNE2 | EEPD1 | HMGCR | MICAL1 | PIH1D1 | SAT1 | TGM1 | ZNF16 |
| ADAL | CCNG2 | EFCAB11 | HMGN1 | MICAL2 | PIH1D2 | SATB2 | TGM2 | ZNF160 |
| ADAM10 | CCNH | EFCAB12 | HMGN2 | MICAL3 | PIK3C2B | SAV1 | TGM7 | ZNF169 |
| ADAM12 | CCNI | EFCAB13 | HMGN3 | MICALL2 | PIK3CA | SAXO1 | TGOLN2 | ZNF174 |
| ADAM15 | CCNJL | EFCAB14 | HMGN4 | MICB | PIK3CB | SAXO2 | TGS1 | ZNF18 |
| ADAM17 | CCNK | EFCAB5 | HMGXB3 | MICOS10 | PIK3R2 | SAYSD1 | TH | ZNF180 |
| ADAM21 | CCNL1 | EFCAB7 | HMGXB4 | MICOS10-NBL1 | PIK3R3 | SBF1 | THADA | ZNF181 |
| ADAM22 | CCNL2 | EFEMP2 | HMOX1 | MICOS13 | PIK3R4 | SBF2 | THAP1 | ZNF182 |
| ADAM32 | CCNO | EFHB | HNF4G | MICU1 | PIKFYVE | SBK2 | THAP10 | ZNF184 |
| ADAM8 | CCNQ | EFHC1 | HNRNPA0 | MICU2 | PIM1 | SBK3 | THAP12 | ZNF195 |
| ADAM9 | CCNT1 | EFHD2 | HNRNPA1 | MID1IP1 | PIM2 | SBNO1 | THAP4 | ZNF2 |
| ADAMTS1 | CCNT2 | EFL1 | HNRNPA2B1 | MIDEAS | PIM3 | SBNO2 | THAP5 | ZNF200 |
| ADAMTS15 | CCNYL1 | EFNB2 | HNRNPA3 | MIDN | PINK1 | SBSN | THAP6 | ZNF205 |
| ADAMTS4 | CCP110 | EFR3A | HNRNPAB | MIEF2 | PIP4K2A | SC5D | THAP7 | ZNF207 |
| ADAMTS5 | CCPG1 | EFR3B | HNRNPC | MIEN1 | PIP4K2B | SCAF11 | THAP8 | ZNF212 |
| ADAMTS6 | CCR10 | EFS | HNRNPD | MIER1 | PIP4K2C | SCAF4 | THAP9 | ZNF213 |
| ADAMTS8 | CCR3 | EFTUD2 | HNRNPDL | MIER2 | PIP4P1 | SCAF8 | THBS1 | ZNF217 |
| ADAMTSL4 | CCS | EGFR | HNRNPF | MIER3 | PIP5K1A | SCAI | THBS3 | ZNF219 |
| ADAMTSL5 | CCSAP | EGLN1 | HNRNPH1 | MIF | PIP5K1C | SCAMP1 | THEM4 | ZNF221 |
| ADAR | CCSER2 | EGLN2 | HNRNPH2 | MIF4GD | PIP5KL1 | SCAMP2 | THEM5 | ZNF23 |
| ADAT1 | CCT3 | EGLN3 | HNRNPH3 | MIGA1 | PIR | SCAMP3 | THEM6 | ZNF232 |
| ADAT2 | CCT4 | EGR3 | HNRNPK | MIGA2 | PISD | SCAMP4 | THG1L | ZNF233 |
| ADAT3 | CCT5 | EGR4 | HNRNPL | MINDY1 | PITPNA | SCAMP5 | THNSL1 | ZNF236 |
| ADCK1 | CCT6A | EHBP1L1 | HNRNPLL | MINDY2 | PITPNB | SCAND1 | THOC1 | ZNF239 |
| ADCK5 | CCT6B | EHD1 | HNRNPR | MINK1 | PITPNC1 | SCAPER | THOC5 | ZNF24 |
| ADCY10 | CCT7 | EHMT1 | HNRNPU | MINPP1 | PITPNM1 | SCARA5 | THOC6 | ZNF248 |
| ADCY3 | CCT8 | EHMT2 | HNRNPUL2 | MIOS | PITPNM3 | SCARB1 | THOC7 | ZNF25 |
| ADCY6 | CD109 | EID1 | HNRNPUL2-BSCL2 | MIOX | PITRM1 | SCARB2 | THOP1 | ZNF250 |
| ADCY9 | CD14 | EID2 | HOMER1 | MIP | PITX1 | SCARF1 | THRAP3 | ZNF251 |
| ADD1 | CD151 | EID2B | HOMER3 | MIPEP | PITX2 | SCCPDH | THRB | ZNF254 |
| ADGRA2 | CD164 | EIF1 | HOMEZ | MIPOL1 | PITX3 | SCD | THSD8 | ZNF260 |
| ADGRA3 | CD180 | EIF1AD | HOOK2 | MIS12 | PJA2 | SCFD1 | THTPA | ZNF263 |
| ADGRB2 | CD200R1 | EIF1AX | HOOK3 | MIS18A | PJVK | SCFD2 | THUMPD2 | ZNF266 |
| ADGRE2 | CD226 | EIF1B | HOXA1 | MIS18BP1 | PKD1 | SCG2 | THUMPD3 | ZNF268 |
| ADGRE3 | CD24 | EIF2A | HOXA10 | MISP | PKD1L1 | SCGB1D2 | THYN1 | ZNF274 |
| ADGRE5 | CD247 | EIF2AK1 | HOXA11 | MISP3 | PKD1L2 | SCGB1D4 | TIA1 | ZNF275 |
| ADGRF1 | CD248 | EIF2AK3 | HOXA2 | MIX23 | PKD1L3 | SCGB2B2 | TIAL1 | ZNF280B |
| ADGRF5 | CD274 | EIF2AK4 | HOXA4 | MKKS | PKD2L1 | SCIN | TICAM1 | ZNF280C |
| ADGRG3 | CD276 | EIF2B1 | HOXA5 | MKLN1 | PKD2L2 | SCLT1 | TICAM2 | ZNF280D |
| ADGRG6 | CD2AP | EIF2B2 | HOXA6 | MKNK1 | PKIG | SCLY | TICRR | ZNF281 |
| ADI1 | CD2BP2 | EIF2B3 | HOXA7 | MKNK2 | PKLR | SCML2 | TIFA | ZNF282 |
| ADIPOR1 | CD300LD | EIF2B4 | HOXA9 | MKRN1 | PKM | SCN11A | TIFAB | ZNF287 |
| ADIPOR2 | CD300LG | EIF2B5 | HOXB4 | MKRN2 | PKMYT1 | SCN2B | TIGAR | ZNF296 |
| ADK | CD320 | EIF2D | HOXB5 | MKRN2OS | PKN2 | SCN4B | TIGD1 | ZNF3 |
| ADM | CD3D | EIF2S1 | HOXB6 | MKS1 | PKN3 | SCNM1 | TIGD3 | ZNF300 |
| ADNP | CD44 | EIF2S2 | HOXB7 | MLEC | PKNOX1 | SCNN1A | TIGD4 | ZNF302 |
| ADPGK | CD46 | EIF3A | HOXB8 | MLF2 | PKP2 | SCO1 | TIGD5 | ZNF318 |
| ADPRHL1 | CD47 | EIF3B | HOXB9 | MLH1 | PKP3 | SCO2 | TIGD6 | ZNF319 |
| ADPRM | CD55 | EIF3D | HOXC10 | MLH3 | PLA2G12A | SCPEP1 | TIMELESS | ZNF320 |
| ADPRS | CD58 | EIF3G | HOXC13 | MLKL | PLA2G12B | SCRIB | TIMM10B | ZNF322 |
| ADRA2A | CD59 | EIF3H | HOXC9 | MLLT3 | PLA2G15 | SCRN2 | TIMM17A | ZNF324B |
| ADRA2B | CD63 | EIF3J | HOXD9 | MLLT6 | PLA2G3 | SCRT1 | TIMM17B | ZNF326 |
| ADRB2 | CD69 | EIF3L | HP1BP3 | MLN | PLA2G4E | SCYGR1 | TIMM21 | ZNF329 |
| ADRB3 | CD7 | EIF4A1 | HPCAL1 | MLX | PLA2G6 | SCYL3 | TIMM22 | ZNF331 |
| ADRM1 | CD70 | EIF4A2 | HPDL | MLXIP | PLA2G7 | SDAD1 | TIMM44 | ZNF333 |
| ADSL | CD72 | EIF4A3 | HPRT1 | MLXIPL | PLA2R1 | SDC1 | TIMM50 | ZNF335 |
| ADSS1 | CD79B | EIF4B | HPS1 | MLYCD | PLAA | SDC3 | TIMM8A | ZNF337 |
| ADSS2 | CD82 | EIF4E | HPS3 | MMAB | PLAAT2 | SDC4 | TIMM8B | ZNF33A |
| AFAP1 | CD83 | EIF4E2 | HPS4 | MMD | PLAAT3 | SDCBP | TIMM9 | ZNF33B |
| AFAP1L1 | CD99 | EIF4EBP1 | HPS5 | MMEL1 | PLAC1 | SDCCAG8 | TIMMDC1 | ZNF34 |
| AFAP1L2 | CD99L2 | EIF4EBP2 | HPS6 | MMGT1 | PLAC8L1 | SDE2 | TIMP1 | ZNF341 |
| AFDN | CDADC1 | EIF4ENIF1 | HPSE | MMP1 | PLAGL2 | SDF2L1 | TIMP2 | ZNF343 |
| AFF1 | CDC14B | EIF4G1 | HR | MMP10 | PLAT | SDF4 | TIMP3 | ZNF35 |
| AFF4 | CDC16 | EIF4G2 | HRAS | MMP13 | PLAUR | SDHA | TIMP4 | ZNF354A |
| AFG1L | CDC20 | EIF4G3 | HRC | MMP15 | PLCB2 | SDHAF1 | TINAGL1 | ZNF354B |
| AFG3L2 | CDC20B | EIF4H | HRH1 | MMP24OS | PLCB3 | SDHAF3 | TINCR | ZNF367 |
| AFTPH | CDC23 | EIF5 | HRH3 | MMP28 | PLCD1 | SDHAF4 | TINF2 | ZNF37A |
| AGA | CDC25A | EIF5A2 | HRK | MMP3 | PLCD3 | SDHB | TIPARP | ZNF383 |
| AGAP1 | CDC25C | EIF5B | HROB | MMRN2 | PLCD4 | SDHC | TIPIN | ZNF384 |
| AGAP2 | CDC26 | EIF6 | HRURF | MMS19 | PLCG1 | SDHD | TIPRL | ZNF385A |
| AGAP3 | CDC27 | EIPR1 | HS1BP3 | MMS22L | PLCG2 | SDR39U1 | TIRAP | ZNF385C |
| AGAP9 | CDC34 | ELAC1 | HS2ST1 | MMUT | PLCL2 | SDR9C7 | TJAP1 | ZNF394 |
| AGBL3 | CDC37 | ELAC2 | HS3ST1 | MN1 | PLCXD1 | SDS | TJP1 | ZNF395 |
| AGER | CDC40 | ELANE | HS3ST6 | MNAT1 | PLD1 | SDSL | TK1 | ZNF397 |
| AGFG1 | CDC42 | ELAPOR2 | HS6ST2 | MNT | PLD2 | SEBOX | TK2 | ZNF398 |
| AGGF1 | CDC42BPA | ELAVL1 | HSBP1 | MNX1 | PLD3 | SEC11A | TKFC | ZNF408 |
| AGK | CDC42BPB | ELAVL3 | HSBP1L1 | MOAP1 | PLD4 | SEC13 | TKT | ZNF41 |
| AGL | CDC42BPG | ELF2 | HSCB | MOB1A | PLD5P1 | SEC14L2 | TKTL2 | ZNF410 |
| AGMAT | CDC42EP1 | ELF3 | HSD17B10 | MOB1B | PLD6 | SEC14L3 | TLCD1 | ZNF414 |
| AGO2 | CDC42EP2 | ELF4 | HSD17B14 | MOB3B | PLEC | SEC14L4 | TLCD2 | ZNF417 |
| AGO3 | CDC42EP4 | ELFN2 | HSD17B4 | MOB3C | PLEK2 | SEC14L5 | TLCD3A | ZNF420 |
| AGO4 | CDC42EP5 | ELK1 | HSD17B6 | MOCOS | PLEKHA1 | SEC14L6 | TLCD3B | ZNF425 |
| AGPAT1 | CDC42SE1 | ELK4 | HSD17B8 | MOCS2 | PLEKHA2 | SEC16A | TLCD4 | ZNF426 |
| AGPAT2 | CDC42SE2 | ELL | HSD3B7 | MOGAT3 | PLEKHA3 | SEC22B | TLCD4-RWDD3 | ZNF428 |
| AGPAT3 | CDC45 | ELL2 | HSDL1 | MOGS | PLEKHA4 | SEC22C | TLCD5 | ZNF430 |
| AGPAT5 | CDC6 | ELL3 | HSDL2 | MON1A | PLEKHA5 | SEC23A | TLE2 | ZNF431 |
| AGPS | CDC7 | ELMO2 | HSF1 | MON1B | PLEKHA6 | SEC23IP | TLE3 | ZNF433 |
| AGRP | CDC73 | ELMOD2 | HSF2 | MON2 | PLEKHA8 | SEC24A | TLE4 | ZNF436 |
| AGTPBP1 | CDCA2 | ELMOD3 | HSF4 | MORC2 | PLEKHG2 | SEC24B | TLE5 | ZNF438 |
| AGTRAP | CDCA3 | ELOA | HSP90AA1 | MORC3 | PLEKHG3 | SEC31A | TLE6 | ZNF44 |
| AHCY | CDCA4 | ELOB | HSP90AB1 | MORC4 | PLEKHG5 | SEC31B | TLE7 | ZNF441 |
| AHCYL1 | CDCA5 | ELOVL1 | HSP90B1 | MORN1 | PLEKHH1 | SEC61A1 | TLN1 | ZNF443 |
| AHCYL2 | CDCA7 | ELOVL5 | HSPA12A | MORN2 | PLEKHH3 | SEC61A2 | TLN2 | ZNF444 |
| AHI1 | CDCA7L | ELOVL6 | HSPA13 | MORN3 | PLEKHJ1 | SEC61G | TLNRD1 | ZNF445 |
| AHNAK | CDCP1 | ELOVL7 | HSPA14 | MORN4 | PLEKHM1 | SEC62 | TLR6 | ZNF449 |
| AHNAK2 | CDCP2 | ELP1 | HSPA1A | MOSPD1 | PLEKHM3 | SEC63 | TLR9 | ZNF45 |
| AHRR | CDH16 | ELP2 | HSPA1B | MOSPD2 | PLEKHN1 | SECISBP2 | TLX3 | ZNF451 |
| AICDA | CDH22 | ELP4 | HSPA1L | MOV10 | PLEKHS1 | SECISBP2L | TM2D1 | ZNF460 |
| AIDA | CDH24 | ELP5 | HSPA4 | MPC1 | PLGRKT | SECTM1 | TM2D2 | ZNF461 |
| AIFM1 | CDHR2 | ELP6 | HSPA4L | MPC2 | PLIN1 | SEH1L | TM2D3 | ZNF469 |
| AIFM2 | CDHR4 | EMC1 | HSPA5 | MPHOSPH10 | PLIN2 | SEL1L | TM4SF1 | ZNF48 |
| AIMP2 | CDIP1 | EMC10 | HSPA6 | MPHOSPH6 | PLIN3 | SEL1L2 | TM4SF19 | ZNF484 |
| AIP | CDIPT | EMC4 | HSPA8 | MPHOSPH8 | PLIN5 | SEL1L3 | TM4SF19-DYNLT2B | ZNF487 |
| AIPL1 | CDK1 | EMC6 | HSPA9 | MPHOSPH9 | PLK1 | SELENBP1 | TM4SF20 | ZNF488 |
| AJM1 | CDK11A | EMC7 | HSPB11 | MPI | PLK2 | SELENOF | TM4SF4 | ZNF496 |
| AJUBA | CDK11B | EMC8 | HSPB6 | MPND | PLK3 | SELENOH | TM4SF5 | ZNF497 |
| AK1 | CDK12 | EMC9 | HSPB7 | MPP1 | PLK4 | SELENOM | TM7SF3 | ZNF500 |
| AK2 | CDK13 | EMG1 | HSPB8 | MPP3 | PLK5 | SELENOS | TM9SF1 | ZNF503 |
| AK3 | CDK16 | EMILIN3 | HSPBAP1 | MPP7 | PLLP | SELENOW | TM9SF3 | ZNF507 |
| AK4P3 | CDK17 | EML1 | HSPD1 | MPPE1 | PLOD3 | SELPLG | TMA16 | ZNF511 |
| AK6 | CDK18 | EML2 | HSPE1 | MPST | PLP2 | SEMA3A | TMBIM1 | ZNF511-PRAP1 |
| AK7 | CDK19 | EML3 | HSPE1-MOB4 | MPZ | PLPBP | SEMA3C | TMBIM4 | ZNF512 |
| AK8 | CDK2 | EML4 | HSPG2 | MPZL1 | PLPP1 | SEMA3E | TMBIM6 | ZNF512B |
| AK9 | CDK2AP1 | EML5 | HSPH1 | MPZL2 | PLPP2 | SEMA3F | TMC4 | ZNF513 |
| AKAP1 | CDK2AP2 | EML6 | HTATIP2 | MPZL3 | PLPP5 | SEMA3G | TMC5 | ZNF514 |
| AKAP10 | CDK3 | EMP2 | HTD2 | MRAS | PLPP6 | SEMA4B | TMC6 | ZNF517 |
| AKAP11 | CDK4 | EMP3 | HTR1B | MRE11 | PLPPR3 | SEMA4C | TMCC2 | ZNF518A |
| AKAP13 | CDK5 | EMSY | HTR1D | MREG | PLRG1 | SEMA4D | TMCO1 | ZNF524 |
| AKAP17A | CDK6 | EN1 | HTR3A | MRFAP1L1 | PLS1 | SEMA4F | TMCO3 | ZNF526 |
| AKAP7 | CDK8 | ENC1 | HTR5A | MRGBP | PLS3 | SEMA6B | TMCO4 | ZNF527 |
| AKAP8 | CDK9 | ENDOG | HTRA2 | MRGPRD | PLSCR1 | SEMA6C | TMCO6 | ZNF529 |
| AKAP8L | CDKL1 | ENDOU | HUS1 | MRGPRG | PLSCR3 | SEMA7A | TMED1 | ZNF541 |
| AKAP9 | CDKL3 | ENDOV | HVCN1 | MRI1 | PLSCR4 | SENP5 | TMED4 | ZNF544 |
| AKIP1 | CDKN1B | ENG | HYAL1 | MRM1 | PLTP | SENP6 | TMED5 | ZNF546 |
| AKIRIN1 | CDKN1C | ENGASE | HYAL2 | MRM2 | PLVAP | SENP7 | TMED7 | ZNF547 |
| AKIRIN2 | CDKN2A | ENKD1 | HYAL3 | MRM3 | PLXDC1 | SEPHS2 | TMED7-TICAM2 | ZNF548 |
| AKNAD1 | CDKN2AIP | ENO1 | HYI | MRNIP | PLXNA1 | SEPSECS | TMEFF2 | ZNF552 |
| AKR1A1 | CDKN2AIPNL | ENO3 | HYKK | MRO | PLXNA3 | SEPTIN1 | TMEM100 | ZNF554 |
| AKR1B1 | CDKN2B | ENOPH1 | HYOU1 | MROH1 | PLXNB1 | SEPTIN10 | TMEM106B | ZNF555 |
| AKR1C2 | CDKN2C | ENOX1 | IAH1 | MROH6 | PLXND1 | SEPTIN11 | TMEM107 | ZNF557 |
| AKR7A2 | CDKN2D | ENOX2 | IARS1 | MROH8 | PM20D2 | SEPTIN12 | TMEM11 | ZNF558 |
| AKR7A3 | CDKN3 | ENPP2 | IARS2 | MRPL1 | PMEL | SEPTIN2 | TMEM115 | ZNF564 |
| AKT1 | CDNF | ENPP3 | IBTK | MRPL10 | PMEPA1 | SEPTIN4 | TMEM116 | ZNF565 |
| AKT1S1 | CDO1 | ENTPD2 | ICA1 | MRPL12 | PMF1 | SEPTIN6 | TMEM117 | ZNF566 |
| AKT2 | CDPF1 | ENTPD4 | ICA1L | MRPL14 | PMF1-BGLAP | SEPTIN7 | TMEM119 | ZNF567 |
| AKT3 | CDR2 | ENTPD5 | ICAM1 | MRPL16 | PMPCA | SERBP1 | TMEM120A | ZNF57 |
| AKTIP | CDRT4 | ENTPD6 | ICAM3 | MRPL17 | PMPCB | SERF2 | TMEM123 | ZNF571 |
| ALAD | CDS2 | ENTPD7 | ICE1 | MRPL18 | PMS1 | SERGEF | TMEM126A | ZNF573 |
| ALAS1 | CDSN | ENTPD8 | ICE2 | MRPL2 | PMS2 | SERINC3 | TMEM126B | ZNF575 |
| ALCAM | CDV3 | ENTR1 | ICMT | MRPL20 | PMVK | SERINC4 | TMEM127 | ZNF576 |
| ALDH1A2 | CDYL | ENY2 | ICOSLG | MRPL21 | PNKP | SERINC5 | TMEM129 | ZNF579 |
| ALDH1A3 | CDYL2 | EOGT | ID2 | MRPL24 | PNMA1 | SERP1 | TMEM131L | ZNF580 |
| ALDH1B1 | CEACAM7 | EOLA2 | ID3 | MRPL27 | PNMA6E | SERPINA9 | TMEM132A | ZNF584 |
| ALDH1L1 | CEBPA | EP300 | IDH1 | MRPL28 | PNMA6F | SERPINB1 | TMEM134 | ZNF585B |
| ALDH1L2 | CEBPD | EP400 | IDH2 | MRPL3 | PNMT | SERPINB6 | TMEM135 | ZNF587B |
| ALDH3A1 | CEBPE | EPAS1 | IDH3B | MRPL33 | PNN | SERPINB8 | TMEM138 | ZNF589 |
| ALDH3A2 | CEBPG | EPB41L1 | IDH3G | MRPL34 | PNO1 | SERPINE1 | TMEM141 | ZNF592 |
| ALDH3B2 | CEBPZOS | EPB41L2 | IDI1 | MRPL35 | PNPLA4 | SERPINE2 | TMEM143 | ZNF593 |
| ALDH4A1 | CELA1 | EPB41L4A | IDI2 | MRPL36 | PNPLA5 | SERPINI1 | TMEM147 | ZNF593OS |
| ALDH5A1 | CELF1 | EPB41L4B | IDS | MRPL37 | PNPLA7 | SERTAD1 | TMEM14A | ZNF597 |
| ALDH6A1 | CELF3 | EPB41L5 | IDUA | MRPL38 | PNPLA8 | SERTAD2 | TMEM14B | ZNF598 |
| ALDH7A1 | CELF4 | EPC1 | IER2 | MRPL40 | PNPO | SERTAD3 | TMEM14C | ZNF605 |
| ALDH9A1 | CELSR1 | EPCAM | IER3 | MRPL42 | PNPT1 | SESN1 | TMEM150A | ZNF607 |
| ALDOA | CELSR3 | EPDR1 | IER3IP1 | MRPL43 | PNRC1 | SESN2 | TMEM150B | ZNF608 |
| ALDOB | CEMIP2 | EPG5 | IER5 | MRPL44 | POC1A | SESTD1 | TMEM156 | ZNF609 |
| ALDOC | CEMP1 | EPGN | IER5L | MRPL45 | POC1B | SET | TMEM158 | ZNF621 |
| ALG1 | CENATAC | EPHA2 | IFFO1 | MRPL48 | POC1B-GALNT4 | SETBP1 | TMEM160 | ZNF622 |
| ALG10 | CEND1 | EPHA6 | IFFO2 | MRPL49 | POC5 | SETD1A | TMEM161A | ZNF623 |
| ALG10B | CENPA | EPHB4 | IFI16 | MRPL51 | PODNL1 | SETD2 | TMEM164 | ZNF624 |
| ALG11 | CENPB | EPHX1 | IFI27L1 | MRPL52 | PODXL | SETD3 | TMEM165 | ZNF627 |
| ALG12 | CENPC | EPHX3 | IFI30 | MRPL53 | POFUT1 | SETD4 | TMEM167A | ZNF628 |
| ALG13 | CENPE | EPM2A | IFI35 | MRPL55 | POFUT2 | SETD5 | TMEM167B | ZNF629 |
| ALG2 | CENPF | EPM2AIP1 | IFI6 | MRPL57 | POGK | SETD7 | TMEM168 | ZNF638 |
| ALG3 | CENPH | EPN1 | IFIH1 | MRPL58 | POGLUT1 | SETD9 | TMEM170A | ZNF639 |
| ALG6 | CENPJ | EPN2 | IFITM1 | MRPL9 | POGLUT2 | SETDB1 | TMEM170B | ZNF641 |
| ALG8 | CENPL | EPN3 | IFITM10 | MRPS10 | POGLUT3 | SETDB2 | TMEM175 | ZNF644 |
| ALG9 | CENPM | EPOP | IFITM3 | MRPS12 | POGZ | SETMAR | TMEM179B | ZNF646 |
| ALK | CENPN | EPOR | IFITM5 | MRPS14 | POLA1 | SETSIP | TMEM18 | ZNF648 |
| ALKBH2 | CENPO | EPPIN | IFNA5 | MRPS15 | POLD1 | SETX | TMEM183A | ZNF652 |
| ALKBH4 | CENPQ | EPPK1 | IFNAR1 | MRPS16 | POLD2 | SEZ6L2 | TMEM184A | ZNF653 |
| ALKBH5 | CENPS | EPRS1 | IFNB1 | MRPS17 | POLD3 | SF1 | TMEM184B | ZNF654 |
| ALKBH6 | CENPS-CORT | EPS15 | IFNGR1 | MRPS18A | POLD4 | SF3A1 | TMEM185B | ZNF664 |
| ALKBH7 | CENPT | EPS15L1 | IFNL3 | MRPS18C | POLDIP2 | SF3A2 | TMEM187 | ZNF668 |
| ALMS1 | CENPU | EPS8 | IFNLR1 | MRPS2 | POLDIP3 | SF3A3 | TMEM19 | ZNF672 |
| ALOX12B | CENPV | EPS8L1 | IFNW1 | MRPS23 | POLE | SF3B1 | TMEM190 | ZNF674 |
| ALOXE3 | CENPX | EPS8L3 | IFRD1 | MRPS24 | POLE3 | SF3B2 | TMEM198 | ZNF675 |
| ALPK2 | CEP104 | EQTN | IFRD2 | MRPS26 | POLE4 | SF3B4 | TMEM200B | ZNF678 |
| ALPP | CEP112 | ERAL1 | IFT122 | MRPS30 | POLG | SF3B6 | TMEM201 | ZNF680 |
| ALS2 | CEP120 | ERAP1 | IFT140 | MRPS31 | POLG2 | SFI1 | TMEM203 | ZNF683 |
| ALS2CL | CEP128 | ERAP2 | IFT20 | MRPS34 | POLH | SFMBT1 | TMEM205 | ZNF687 |
| ALX1 | CEP131 | ERBB3 | IFT22 | MRPS35 | POLI | SFMBT2 | TMEM208 | ZNF688 |
| ALX3 | CEP135 | ERC1 | IFT27 | MRPS36 | POLK | SFN | TMEM209 | ZNF689 |
| ALX4 | CEP152 | ERCC1 | IFT52 | MRPS5 | POLM | SFR1 | TMEM210 | ZNF692 |
| ALYREF | CEP164 | ERCC6 | IFT74 | MRPS7 | POLR1B | SFRP4 | TMEM214 | ZNF695 |
| AMACR | CEP170 | ERF | IFT81 | MRPS9 | POLR1C | SFT2D2 | TMEM216 | ZNF696 |
| AMBP | CEP170B | ERFE | IFT88 | MRRF | POLR1D | SFT2D3 | TMEM218 | ZNF697 |
| AMBRA1 | CEP19 | ERG28 | IFTAP | MRS2 | POLR1E | SFTA2 | TMEM220 | ZNF7 |
| AMD1 | CEP192 | ERGIC1 | IGDCC3 | MRTFA | POLR1G | SFTPB | TMEM221 | ZNF70 |
| AMDHD1 | CEP20 | ERGIC2 | IGF1R | MRTFB | POLR1H | SFTPC | TMEM222 | ZNF701 |
| AMDHD2 | CEP250 | ERI1 | IGF2BP1 | MRTO4 | POLR2A | SFXN1 | TMEM223 | ZNF703 |
| AMFR | CEP290 | ERI2 | IGF2BP2 | MSANTD2 | POLR2C | SFXN2 | TMEM230 | ZNF706 |
| AMH | CEP295 | ERI3 | IGF2BP3 | MSANTD3 | POLR2D | SFXN3 | TMEM231 | ZNF707 |
| AMIGO1 | CEP295NL | ERICH1 | IGFBP4 | MSC | POLR2E | SFXN4 | TMEM232 | ZNF708 |
| AMIGO2 | CEP350 | ERICH6 | IGFBP6 | MSH3 | POLR2H | SFXN5 | TMEM234 | ZNF714 |
| AMIGO3 | CEP41 | ERLIN1 | IGFBP7 | MSH5 | POLR2I | SGCE | TMEM237 | ZNF721 |
| AMMECR1 | CEP43 | ERLIN2 | IGFL3 | MSH5-SAPCD1 | POLR2J | SGF29 | TMEM238 | ZNF738 |
| AMN1 | CEP55 | ERMAP | IGFL4 | MSI2 | POLR2K | SGK1 | TMEM240 | ZNF740 |
| AMOTL2 | CEP57 | ERMARD | IGFLR1 | MSL1 | POLR2L | SGK3 | TMEM241 | ZNF746 |
| AMPD1 | CEP57L1 | ERMP1 | IGHMBP2 | MSL2 | POLR2M | SGMS1 | TMEM242 | ZNF747 |
| AMT | CEP63 | ERN1 | IGSF10 | MSL3 | POLR3A | SGMS2 | TMEM243 | ZNF749 |
| AMZ2 | CEP68 | ERO1A | IGSF3 | MSLN | POLR3B | SGO1 | TMEM245 | ZNF750 |
| ANAPC1 | CEP70 | ERO1B | IKBIP | MSLNL | POLR3C | SGO2 | TMEM248 | ZNF75D |
| ANAPC10 | CEP76 | ERP27 | IKBKG | MSMO1 | POLR3D | SGPL1 | TMEM249 | ZNF76 |
| ANAPC11 | CEP78 | ERP29 | IKZF2 | MSMP | POLR3E | SGPP1 | TMEM250 | ZNF764 |
| ANAPC13 | CEP83 | ERP44 | IKZF5 | MSN | POLR3F | SGSH | TMEM251 | ZNF766 |
| ANAPC15 | CEP85 | ERRFI1 | IL11 | MSRA | POLR3G | SGSM3 | TMEM256 | ZNF768 |
| ANAPC16 | CEP85L | ERVH48-1 | IL12B | MSRB1 | POLR3H | SGTA | TMEM258 | ZNF77 |
| ANAPC2 | CEP89 | ERVK3-1 | IL12RB1 | MSRB2 | POLR3K | SGTB | TMEM259 | ZNF770 |
| ANAPC4 | CEP95 | ESAM | IL13RA1 | MSRB3 | POLRMT | SH2B1 | TMEM260 | ZNF771 |
| ANAPC5 | CEPT1 | ESCO1 | IL15 | MST1 | POM121 | SH2B3 | TMEM262 | ZNF774 |
| ANAPC7 | CERK | ESCO2 | IL15RA | MST1R | POM121C | SH2D3A | TMEM263 | ZNF775 |
| ANG | CERS1 | ESF1 | IL17B | MSTO1 | POMGNT1 | SH2D3C | TMEM267 | ZNF777 |
| ANGEL1 | CERS2 | ESPL1 | IL17D | MSX1 | POMP | SH2D4A | TMEM268 | ZNF778 |
| ANGPTL2 | CERS5 | ESRP2 | IL17RB | MT1G | POMT1 | SH2D7 | TMEM33 | ZNF780A |
| ANGPTL6 | CERT1 | ESRRA | IL17RD | MT1X | POMT2 | SH3BGRL3 | TMEM35B | ZNF782 |
| ANK1 | CES1 | ESS2 | IL17REL | MT2A | PON2 | SH3BP5L | TMEM38A | ZNF783 |
| ANK3 | CETN3 | ESYT1 | IL18 | MTA1 | POP1 | SH3D21 | TMEM38B | ZNF784 |
| ANKAR | CETP | ESYT2 | IL18R1 | MTA2 | POP5 | SH3GL1 | TMEM39A | ZNF785 |
| ANKFY1 | CFAP119 | ETF1 | IL1A | MTA3 | POP7 | SH3GL2 | TMEM39B | ZNF786 |
| ANKH | CFAP141 | ETFA | IL1RAP | MTAP | POPDC2 | SH3GLB1 | TMEM40 | ZNF787 |
| ANKHD1 | CFAP20 | ETFB | IL20 | MTARC1 | POPDC3 | SH3GLB2 | TMEM41A | ZNF79 |
| ANKHD1-EIF4EBP3 | CFAP206 | ETFBKMT | IL20RA | MTCH1 | POR | SH3PXD2A | TMEM41B | ZNF792 |
| ANKIB1 | CFAP210 | ETHE1 | IL22RA2 | MTCH2 | PORCN | SH3RF1 | TMEM42 | ZNF799 |
| ANKLE1 | CFAP276 | ETNK1 | IL27RA | MTCP1 | POT1 | SH3TC2 | TMEM43 | ZNF8 |
| ANKMY1 | CFAP298 | ETNPPL | IL2RA | MTDH | POU2F1 | SHARPIN | TMEM44 | ZNF800 |
| ANKMY2 | CFAP298-TCP10L | ETS2 | IL2RB | MTERF3 | POU2F2 | SHB | TMEM45A | ZNF805 |
| ANKRA2 | CFAP36 | ETV3 | IL2RG | MTERF4 | POU3F1 | SHBG | TMEM45B | ZNF808 |
| ANKRD1 | CFAP410 | ETV4 | IL5 | MTF1 | POU3F2 | SHC1 | TMEM47 | ZNF814 |
| ANKRD10 | CFAP418 | ETV5 | IL5RA | MTF2 | POU5F1 | SHC2 | TMEM50B | ZNF816 |
| ANKRD11 | CFAP43 | ETV6 | IL6R | MTFMT | POU6F1 | SHC4 | TMEM51 | ZNF816-ZNF321P |
| ANKRD12 | CFAP45 | EVA1B | IL6ST | MTFP1 | PP2D1 | SHCBP1 | TMEM52 | ZNF821 |
| ANKRD13A | CFAP52 | EVA1C | IL7 | MTFR1 | PPA1 | SHCBP1L | TMEM52B | ZNF823 |
| ANKRD13B | CFAP53 | EVI5 | IL9 | MTFR1L | PPA2 | SHE | TMEM59 | ZNF827 |
| ANKRD13C | CFAP54 | EVPL | ILF2 | MTFR2 | PPARA | SHF | TMEM60 | ZNF830 |
| ANKRD13D | CFAP57 | EVPLL | ILKAP | MTG1 | PPARD | SHFL | TMEM63A | ZNF836 |
| ANKRD16 | CFAP61 | EWSR1 | ILRUN | MTG2 | PPARG | SHH | TMEM63B | ZNF837 |
| ANKRD17 | CFAP65 | EXD1 | ILVBL | MTHFR | PPARGC1A | SHISA5 | TMEM64 | ZNF839 |
| ANKRD18A | CFAP69 | EXD2 | IMMP1L | MTHFS | PPARGC1B | SHISA7 | TMEM65 | ZNF84 |
| ANKRD18B | CFAP73 | EXD3 | IMMP2L | MTHFSD | PPAT | SHISA8 | TMEM67 | ZNF843 |
| ANKRD22 | CFAP92 | EXO5 | IMMT | MTIF2 | PPCDC | SHISAL1 | TMEM68 | ZNF852 |
| ANKRD23 | CFAP97 | EXOC2 | IMP3 | MTIF3 | PPCS | SHKBP1 | TMEM69 | ZNF860 |
| ANKRD24 | CFD | EXOC3 | IMPA1 | MTLN | PPDPF | SHLD2 | TMEM71 | ZNF862 |
| ANKRD26 | CFDP1 | EXOC3L1 | IMPACT | MTM1 | PPEF1 | SHMT1 | TMEM79 | ZNF865 |
| ANKRD27 | CFI | EXOC3L2 | IMPDH1 | MTMR10 | PPFIA1 | SHMT2 | TMEM82 | ZNF878 |
| ANKRD29 | CFL1 | EXOC4 | IMPDH2 | MTMR11 | PPFIBP1 | SHOC1 | TMEM87A | ZNF888 |
| ANKRD33B | CFL2 | EXOC5 | INAFM2 | MTMR12 | PPHLN1 | SHOC2 | TMEM87B | ZNF891 |
| ANKRD34A | CFP | EXOC6B | INCA1 | MTMR14 | PPIA | SHOX2 | TMEM89 | ZNF91 |
| ANKRD35 | CGAS | EXOC7 | ING1 | MTMR2 | PPIB | SHPK | TMEM8B | ZNFX1 |
| ANKRD36 | CGB7 | EXOG | ING2 | MTMR3 | PPIC | SHQ1 | TMEM9 | ZNHIT2 |
| ANKRD36B | CGGBP1 | EXOSC1 | ING4 | MTMR4 | PPIE | SHROOM1 | TMEM91 | ZNHIT6 |
| ANKRD37 | CGN | EXOSC10 | ING5 | MTMR6 | PPIG | SHROOM3 | TMEM9B | ZNRD2 |
| ANKRD39 | CGRRF1 | EXOSC2 | INHA | MTMR7 | PPIH | SHTN1 | TMF1 | ZNRF1 |
| ANKRD40 | CHAC1 | EXOSC3 | INHBA | MTMR9 | PPIL1 | SIAH1 | TMIGD1 | ZNRF3 |
| ANKRD40CL | CHAC2 | EXOSC5 | INIP | MTOR | PPIL2 | SIAH2 | TMIGD2 | ZRANB3 |
| ANKRD46 | CHAD | EXOSC7 | INKA1 | MTPAP | PPIL3 | SIDT2 | TMLHE | ZSCAN10 |
| ANKRD49 | CHADL | EXOSC9 | INKA2 | MTR | PPIL4 | SIGIRR | TMOD1 | ZSCAN12 |
| ANKRD50 | CHAF1A | EXPH5 | INO80 | MTRES1 | PPIL6 | SIGLEC1 | TMOD4 | ZSCAN2 |
| ANKRD52 | CHAMP1 | EXT1 | INO80C | MTRF1L | PPIP5K2 | SIGLEC10 | TMPO | ZSCAN20 |
| ANKRD54 | CHCHD10 | EXT2 | INO80E | MTRFR | PPL | SIGLEC11 | TMPPE | ZSCAN21 |
| ANKRD60 | CHCHD2 | EXTL2 | INPP1 | MTRR | PPM1B | SIGMAR1 | TMPRSS3 | ZSCAN22 |
| ANKRD63 | CHCHD3 | EXTL3 | INPP4B | MTSS2 | PPM1D | SIK1 | TMPRSS6 | ZSCAN25 |
| ANKRD9 | CHCHD4 | EYA3 | INPP5B | MTTP | PPM1F | SIK2 | TMSB10 | ZSCAN26 |
| ANKS3 | CHCHD6 | EYA4 | INPP5E | MTX1 | PPM1G | SIM1 | TMSB4X | ZSCAN29 |
| ANKS6 | CHD1 | EZH1 | INPP5F | MTX2 | PPM1H | SIN3A | TMTC1 | ZSCAN30 |
| ANKUB1 | CHD1L | EZH2 | INPP5J | MUC1 | PPM1J | SIN3B | TMTC2 | ZSCAN31 |
| ANLN | CHD4 | EZR | INPPL1 | MUC16 | PPM1K | SINHCAF | TMTC3 | ZSCAN32 |
| ANO6 | CHD5 | F11R | INS | MUC4 | PPM1L | SIPA1 | TMTC4 | ZSWIM3 |
| ANO8 | CHD7 | F12 | INSIG1 | MUC6 | PPM1M | SIPA1L1 | TMUB1 | ZSWIM4 |
| ANO9 | CHD8 | F13B | INS-IGF2 | MUL1 | PPME1 | SIPA1L3 | TMUB2 | ZSWIM5 |
| ANP32A | CHD9 | F3 | INSL3 | MUS81 | PPOX | SIRPB1 | TMX2 | ZSWIM6 |
| ANP32B | CHDH | FA2H | INSR | MUSTN1 | PPP1CA | SIRPD | TMX3 | ZSWIM7 |
| ANP32E | CHEK1 | FAAH2 | INSRR | MUTYH | PPP1CC | SIRT2 | TMX4 | ZSWIM8 |
| ANPEP | CHEK2 | FAAP100 | INTS1 | MVB12A | PPP1R10 | SIRT3 | TNC | ZSWIM9 |
| ANTKMT | CHERP | FAAP20 | INTS10 | MVD | PPP1R11 | SIRT5 | TNFAIP1 | ZUP1 |
| ANTXR1 | CHID1 | FAAP24 | INTS11 | MVK | PPP1R12B | SIRT6 | TNFAIP8 | ZWILCH |
| ANTXR2 | CHKA | FABP12 | INTS12 | MVP | PPP1R12C | SIRT7 | TNFRSF10A | ZXDA |
| ANXA11 | CHKB | FABP3 | INTS13 | MXD1 | PPP1R13B | SIT1 | TNFRSF10B | ZXDB |
| ANXA13 | CHKB-CPT1B | FABP6 | INTS14 | MXD3 | PPP1R13L | SIVA1 | TNFRSF10D | ZXDC |
| ANXA2 | CHML | FADD | INTS15 | MXD4 | PPP1R14A | SIX1 | TNFRSF11A | ZYG11A |
| ANXA2R | CHMP1A | FADS1 | INTS2 | MXI1 | PPP1R14B | SIX2 | TNFRSF11B | ZYG11B |
| ANXA3 | CHMP1B | FADS2 | INTS3 | MXRA7 | PPP1R14D | SIX4 | TNFRSF13C | ZYX |
| ANXA4 | CHMP2A | FADS3 | INTS4 | MXRA8 | PPP1R15A | SIX5 | TNFRSF18 | ZZEF1 |
| ANXA5 | CHMP3 | FADS6 | INTS5 | MYBL1 | PPP1R15B | SKA1 | TNFRSF1A | ZZZ3 |
| ANXA6 | CHMP4A | FAF1 | INTS6 | MYBPC3 | PPP1R16A | SKA2 | TNFRSF21 |  |
| ANXA7 | CHMP4B | FAH | INTS7 | MYBPHL | PPP1R18 | SKA3 | TNFRSF25 |  |
| ANXA8 | CHMP4C | FAHD1 | INTS9 | MYC | PPP1R2 | SKAP2 | TNFRSF4 |  |
| AOPEP | CHMP5 | FAHD2A | INVS | MYCBP | PPP1R26 | SKI | TNFRSF6B | |
| AP1AR | CHMP6 | FAHD2B | IP6K1 | MYCBP2 | PPP1R27 | SKIDA1 | TNFRSF9 |  |
| AP1B1 | CHN1 | FAIM | IP6K3 | MYCL | PPP1R35 | SKIL | TNFSF10 |  |
| AP1G1 | CHORDC1 | FAIM2 | IPMK | MYDGF | PPP1R36 | SKIV2L | TNFSF13 |  |
| AP1G2 | CHP1 | FAM102A | IPO11 | MYG1 | PPP1R37 | SKP1 | TNFSF14 |  |
| AP1M1 | CHPF | FAM102B | IPO13 | MYH10 | PPP1R3B | SKP2 | TNFSF18 |  |
| AP1M2 | CHPT1 | FAM104A | IPO4 | MYH7 | PPP1R3C | SLA | TNFSF8 |  |
| AP1S1 | CHRAC1 | FAM104B | IPO7 | MYH7B | PPP1R3D | SLAIN2 | TNFSF9 |  |
| AP1S2 | CHRDL2 | FAM107B | IPO8 | MYH9 | PPP1R3E | SLBP | TNIK |  |
| AP2A2 | CHRM1 | FAM110A | IPO9 | MYL12A | PPP1R7 | SLC10A3 | TNIP1 |  |
| AP2B1 | CHRM4 | FAM110C | IPP | MYL12B | PPP1R8 | SLC10A5 | TNIP2 |  |
| AP2M1 | CHRNA1 | FAM110D | IPPK | MYL3 | PPP1R9A | SLC10A7 | TNK1 |  |
| AP2S1 | CHRNA3 | FAM114A1 | IQANK1 | MYL5 | PPP1R9B | SLC11A2 | TNK2 |  |
| AP3B1 | CHRNB2 | FAM117A | IQCA1L | MYL6 | PPP2CA | SLC12A2 | TNKS |  |
| AP3B2 | CHST10 | FAM117B | IQCB1 | MYL6B | PPP2CB | SLC12A3 | TNKS1BP1 | |
| AP3D1 | CHST12 | FAM118A | IQCD | MYLIP | PPP2R1A | SLC12A4 | TNKS2 |  |
| AP3M1 | CHST14 | FAM120A | IQCE | MYLK4 | PPP2R1B | SLC12A6 | TNNC1 |  |
| AP3M2 | CHST15 | FAM120AOS | IQCF6 | MYO10 | PPP2R2D | SLC12A7 | TNNC2 |  |
| AP3S2 | CHST3 | FAM120C | IQCH | MYO18A | PPP2R3B | SLC12A8 | TNNI1 |  |
| AP4B1 | CHST5 | FAM126A | IQCN | MYO19 | PPP2R5A | SLC12A9 | TNNI3 |  |
| AP4E1 | CHST6 | FAM126B | IQGAP1 | MYO1A | PPP2R5B | SLC13A3 | TNNT1 |  |
| AP4M1 | CHSY1 | FAM131B | IQGAP3 | MYO1B | PPP2R5E | SLC15A1 | TNNT2 |  |
| AP4S1 | CHTF8 | FAM131C | IQUB | MYO1C | PPP3CA | SLC15A4 | TNPO1 |  |
| AP5B1 | CHTOP | FAM133B | IRAG1 | MYO1D | PPP3CB | SLC16A1 | TNPO2 |  |
| AP5M1 | CHUK | FAM136A | IRAK1BP1 | MYO1F | PPP3CC | SLC16A11 | TNPO3 |  |
| AP5S1 | CHURC1 | FAM13B | IRAK2 | MYO1G | PPP3R1 | SLC16A12 | TNRC6A |  |
| AP5Z1 | CHURC1-FNTB | FAM149B1 | IRAK4 | MYO5A | PPP4C | SLC16A13 | TNS1 |  |
| APAF1 | CIAO1 | FAM151B | IREB2 | MYO6 | PPP4R1 | SLC16A3 | TNS3 |  |
| APBA3 | CIAO2A | FAM161A | IRF1 | MYO9A | PPP4R2 | SLC16A4 | TNS4 |  |
| APBB1 | CIAO2B | FAM161B | IRF2BP1 | MYO9B | PPP4R3A | SLC16A5 | TNXB |  |
| APBB2 | CIAO3 | FAM162A | IRF2BP2 | MYOF | PPP4R3B | SLC16A6 | TOB1 |  |
| APBB3 | CIAPIN1 | FAM163B | IRF2BPL | MYOM1 | PPP5C | SLC16A7 | TOB2 |  |
| APC | CIB1 | FAM166A | IRF3 | MYORG | PPP6C | SLC16A8 | TOE1 |  |
| APEH | CIB2 | FAM167A | IRF6 | MYOZ1 | PPP6R1 | SLC17A3 | TOGARAM1 | |
| APEX1 | CIB3 | FAM168A | IRF7 | MYPN | PPP6R2 | SLC17A5 | TOLLIP |  |
| APEX2 | CIBAR2 | FAM168B | IRF9 | MYPOP | PPRC1 | SLC17A7 | TOM1L2 |  |
| APH1A | CIDEB | FAM171A2 | IRGM | MYSM1 | PPT1 | SLC18A1 | TOMM20 |  |
| API5 | CIDEC | FAM172A | IRGQ | MYT1 | PPT2 | SLC18B1 | TOMM20L | |
| APIP | CILK1 | FAM174B | IRX3 | MYZAP | PPT2-EGFL8 | SLC19A2 | TOMM22 |  |
| APLF | CINP | FAM174C | ISCA1 | MZB1 | PPTC7 | SLC1A1 | TOMM34 |  |
| APLN | CIP2A | FAM178B | ISCA2 | MZF1 | PRADC1 | SLC1A4 | TOMM40 |  |
| APOA1 | CIPC | FAM184B | ISCU | MZT1 | PRAF2 | SLC1A5 | TOMM5 |  |
| APOA2 | CIRBP | FAM185A | ISG15 | MZT2B | PRAG1 | SLC20A1 | TOMM6 |  |
| APOA4 | CISD1 | FAM186B | ISG20L2 | N4BP1 | PRAM1 | SLC22A17 | TOMM7 |  |
| APOBEC3B | CISD2 | FAM187A | ISL2 | N4BP2 | PRAME | SLC22A18 | TOMM70 |  |
| APOBEC4 | CISD3 | FAM187B | ISOC1 | N6AMT1 | PRC1 | SLC22A23 | TONSL |  |
| APOF | CISH | FAM189B | ISOC2 | NAA10 | PRCC | SLC22A31 | TOP1 |  |
| APOL2 | CIT | FAM193B | ISYNA1 | NAA15 | PRCD | SLC22A4 | TOP1MT |  |
| APOM | CITED1 | FAM199X | ITFG1 | NAA16 | PRCP | SLC22A5 | TOP2A |  |
| APOO | CITED2 | FAM200B | ITFG2 | NAA20 | PRDM10 | SLC22A6 | TOP2B |  |
| APOOL | CITED4 | FAM20A | ITGA1 | NAA25 | PRDM14 | SLC23A1 | TOP3A |  |
| APPBP2 | CIZ1 | FAM20B | ITGA10 | NAA30 | PRDM15 | SLC23A3 | TOP3B |  |
| APPL1 | CKAP2L | FAM20C | ITGA11 | NAA35 | PRDM4 | SLC24A1 | TOPORS |  |
| APPL2 | CKAP5 | FAM210A | ITGA2 | NAA38 | PRDM5 | SLC25A1 | TOR1A |  |
| APRT | CKB | FAM210B | ITGA3 | NAA40 | PRDM7 | SLC25A10 | TOR1AIP1 |  |
| APTX | CKLF | FAM214A | ITGA5 | NAA50 | PRDX1 | SLC25A11 | TOR1AIP2 |  |
| AQP11 | CKLF-CMTM1 | FAM214B | ITGA6 | NAA80 | PRDX2 | SLC25A12 | TOR1B |  |
| AQP3 | CKS1B | FAM216A | ITGA7 | NAALADL1 | PRDX3 | SLC25A13 | TOR2A |  |
| AQP6 | CKS2 | FAM219B | ITGAV | NAB1 | PRDX4 | SLC25A15 | TOR3A |  |
| AQP7 | CLASP2 | FAM220A | ITGB1 | NAB2 | PRDX5 | SLC25A16 | TOR4A |  |
| AQR | CLBA1 | FAM221B | ITGB1BP1 | NABP1 | PRDX6 | SLC25A17 | TOX |  |
| ARAF | CLCA2 | FAM222A | ITGB1BP2 | NABP2 | PREB | SLC25A19 | TOX2 |  |
| ARAP1 | CLCC1 | FAM227A | ITGB2 | NACA | PRELID1 | SLC25A2 | TOX4 |  |
| ARAP2 | CLCF1 | FAM227B | ITGB3 | NACAD | PRELID2 | SLC25A20 | TP53 |  |
| ARAP3 | CLCN2 | FAM229B | ITGB3BP | NACC1 | PRELID3A | SLC25A21 | TP53BP1 |  |
| AREG | CLCN3 | FAM234A | ITGB4 | NACC2 | PRELID3B | SLC25A22 | TP53BP2 |  |
| AREL1 | CLCN4 | FAM241A | ITGB5 | NADK | PREPL | SLC25A23 | TP53I11 |  |
| ARF1 | CLCN6 | FAM241B | ITGB6 | NADK2 | PREX1 | SLC25A24 | TP53I3 |  |
| ARF3 | CLCN7 | FAM24B | ITGB7 | NAE1 | PRG2 | SLC25A25 | TP53INP2 |  |
| ARF4 | CLDN1 | FAM25C | ITGB8 | NAGA | PRG3 | SLC25A28 | TP53RK |  |
| ARF5 | CLDN12 | FAM25G | ITIH4 | NAGK | PRICKLE3 | SLC25A29 | TPCN1 |  |
| ARF6 | CLDN15 | FAM3A | ITIH6 | NAGLU | PRICKLE4 | SLC25A3 | TPD52 |  |
| ARFGAP1 | CLDN19 | FAM3C | ITM2C | NAGPA | PRIM1 | SLC25A30 | TPD52L2 |  |
| ARFGAP2 | CLDN23 | FAM47E | ITPK1 | NAIF1 | PRIMPOL | SLC25A32 | TPGS1 |  |
| ARFGAP3 | CLDN3 | FAM50A | ITPKA | NALCN | PRKAA1 | SLC25A33 | TPGS2 |  |
| ARFGEF1 | CLDN4 | FAM50B | ITPKB | NANOS1 | PRKAA2 | SLC25A35 | TPK1 |  |
| ARFGEF3 | CLDN5 | FAM53A | ITPKC | NANOS2 | PRKAB1 | SLC25A36 | TPM2 |  |
| ARFIP1 | CLDN6 | FAM53B | ITPR1 | NAP1L1 | PRKAB2 | SLC25A37 | TPM3 |  |
| ARFIP2 | CLDN7 | FAM53C | ITPR2 | NAP1L4 | PRKACA | SLC25A38 | TPMT |  |
| ARFRP1 | CLDN9 | FAM76A | ITPR3 | NAPA | PRKACB | SLC25A39 | TPP1 |  |
| ARG2 | CLDND1 | FAM76B | ITPRID2 | NAPB | PRKACG | SLC25A4 | TPP2 |  |
| ARGLU1 | CLDND2 | FAM78A | ITPRIP | NAPG | PRKAG3 | SLC25A40 | TPR |  |
| ARHGAP1 | CLEC14A | FAM81A | ITPRIPL2 | NAPRT | PRKAR1A | SLC25A41 | TPRA1 |  |
| ARHGAP10 | CLEC16A | FAM83B | ITSN1 | NAPSA | PRKAR1B | SLC25A42 | TPRG1 |  |
| ARHGAP12 | CLEC1A | FAM83C | ITSN2 | NARF | PRKAR2A | SLC25A43 | TPRN |  |
| ARHGAP17 | CLEC20A | FAM83E | IVD | NARS1 | PRKCA | SLC25A45 | TPRX1 |  |
| ARHGAP18 | CLEC3B | FAM83G | IVNS1ABP | NARS2 | PRKCI | SLC25A5 | TPRX2 |  |
| ARHGAP19 | CLEC7A | FAM83H | IWS1 | NASP | PRKCSH | SLC25A51 | TPT1 |  |
| ARHGAP19-SLIT1 | CLGN | FAM8A1 | IZUMO1 | NAT10 | PRKD3 | SLC25A53 | TPX2 |  |
| ARHGAP21 | CLIC1 | FAM90A1 | IZUMO2 | NAT16 | PRKDC | SLC25A6 | TRA2A |  |
| ARHGAP22 | CLIC3 | FAM91A1 | JADE1 | NATD1 | PRKRA | SLC26A1 | TRA2B |  |
| ARHGAP26 | CLIC4 | FAM98B | JADE2 | NAXD | PRKRIP1 | SLC26A2 | TRABD2A |  |
| ARHGAP27 | CLIC5 | FAM98C | JAG1 | NAXE | PRKX | SLC26A5 | TRABD2B |  |
| ARHGAP28 | CLINT1 | FAM9C | JAG2 | NBDY | PRMT2 | SLC26A6 | TRADD |  |
| ARHGAP29 | CLIP1 | FAN1 | JAGN1 | NBEAL1 | PRMT3 | SLC26A8 | TRAF1 |  |
| ARHGAP30 | CLIP3 | FANCA | JAK1 | NBL1 | PRMT5 | SLC26A9 | TRAF2 |  |
| ARHGAP4 | CLK1 | FANCB | JAK2 | NBN | PRMT9 | SLC27A1 | TRAF3IP1 |  |
| ARHGAP42 | CLK2 | FANCC | JAK3 | NBPF1 | PRNP | SLC27A2 | TRAF3IP2 |  |
| ARHGAP45 | CLK4 | FANCD2 | JARID2 | NBPF11 | PROB1 | SLC27A4 | TRAF4 |  |
| ARHGAP5 | CLMP | FANCD2OS | JCHAIN | NBPF12 | PROCA1 | SLC27A5 | TRAF6 |  |
| ARHGAP9 | CLN3 | FANCE | JHY | NBPF20 | PROS1 | SLC28A3 | TRAIP |  |
| ARHGDIA | CLN6 | FANCF | JKAMP | NBPF9 | PROSER1 | SLC29A1 | TRAK1 |  |
| ARHGDIB | CLNS1A | FANCG | JMJD1C | NBR1 | PROSER3 | SLC29A2 | TRAK2 |  |
| ARHGEF1 | CLPB | FANCI | JMJD4 | NCAPD2 | PROX2 | SLC29A3 | TRAM1 |  |
| ARHGEF11 | CLPSL2 | FANK1 | JMJD6 | NCAPD3 | PRPF18 | SLC2A1 | TRAM2 |  |
| ARHGEF17 | CLPX | FAR1 | JMJD7 | NCAPG | PRPF19 | SLC2A12 | TRAP1 |  |
| ARHGEF19 | CLRN3 | FARP2 | JMJD7-PLA2G4B | NCAPG2 | PRPF3 | SLC2A3 | TRAPPC1 |  |
| ARHGEF2 | CLSPN | FARS2 | JMJD8 | NCAPH2 | PRPF38B | SLC2A4RG | TRAPPC10 | |
| ARHGEF28 | CLSTN1 | FARSA | JMY | NCBP1 | PRPF39 | SLC2A5 | TRAPPC11 | |
| ARHGEF3 | CLTA | FARSB | JOSD1 | NCBP2 | PRPF4 | SLC2A6 | TRAPPC12 | |
| ARHGEF37 | CLTB | FASN | JOSD2 | NCBP2AS2 | PRPF40A | SLC2A8 | TRAPPC14 | |
| ARHGEF39 | CLTC | FASTK | JPH1 | NCBP2L | PRPF6 | SLC30A1 | TRAPPC2 |  |
| ARHGEF40 | CLTCL1 | FASTKD1 | JPH2 | NCBP3 | PRPF8 | SLC30A2 | TRAPPC2B | |
| ARID2 | CLU | FASTKD2 | JPT1 | NCCRP1 | PRPH2 | SLC30A3 | TRAPPC3 |  |
| ARID3A | CLUAP1 | FASTKD3 | JPT2 | NCDN | PRPS2 | SLC30A4 | TRAPPC4 |  |
| ARID3C | CLUH | FASTKD5 | JRK | NCEH1 | PRPSAP1 | SLC30A5 | TRAPPC5 |  |
| ARID4A | CLYBL | FAT1 | JRKL | NCK1 | PRR11 | SLC30A6 | TRAPPC6B | |
| ARID4B | CMAS | FAU | JSRP1 | NCKAP5L | PRR12 | SLC30A7 | TRAPPC8 |  |
| ARIH1 | CMBL | FAXC | JTB | NCKIPSD | PRR13 | SLC30A9 | TRAPPC9 |  |
| ARIH2 | CMC1 | FAXDC2 | JUN | NCLN | PRR14 | SLC33A1 | TRDMT1 |  |
| ARL13B | CMC2 | FBF1 | JUNB | NCOA1 | PRR14L | SLC35A1 | TREH |  |
| ARL14EP | CMC4 | FBH1 | JUND | NCOA2 | PRR15 | SLC35A2 | TRERF1 |  |
| ARL15 | CMIP | FBL | JUP | NCOA3 | PRR15L | SLC35A3 | TREX1 |  |
| ARL16 | CMKLR2 | FBLN1 | KANK1 | NCOA4 | PRR22 | SLC35A4 | TRIAP1 |  |
| ARL2 | CMPK1 | FBN1 | KANK2 | NCOA5 | PRR3 | SLC35A5 | TRIB1 |  |
| ARL2BP | CMSS1 | FBN2 | KANK3 | NCOA6 | PRR30 | SLC35B1 | TRIB3 |  |
| ARL2-SNX15 | CMTM4 | FBP1 | KANK4 | NCOR1 | PRRC1 | SLC35B2 | TRIM11 |  |
| ARL3 | CMTM6 | FBRS | KANSL1 | NCOR2 | PRRC2B | SLC35B3 | TRIM13 |  |
| ARL4A | CMTR2 | FBXL12 | KANSL2 | NCR3 | PRRC2C | SLC35B4 | TRIM14 |  |
| ARL5B | CNBP | FBXL14 | KANSL3 | NCR3LG1 | PRRG2 | SLC35C1 | TRIM16 |  |
| ARL5C | CNDP2 | FBXL16 | KANTR | NCSTN | PRRG4 | SLC35C2 | TRIM17 |  |
| ARL6 | CNEP1R1 | FBXL18 | KARS1 | NDC1 | PRRT1 | SLC35D1 | TRIM25 |  |
| ARL6IP1 | CNFN | FBXL19 | KAT14 | NDC80 | PRRT2 | SLC35D2 | TRIM26 |  |
| ARL6IP4 | CNGA4 | FBXL2 | KAT2A | NDE1 | PRRT3 | SLC35E1 | TRIM27 |  |
| ARL6IP6 | CNGB1 | FBXL3 | KAT2B | NDEL1 | PRRT4 | SLC35E2B | TRIM28 |  |
| ARL8A | CNIH1 | FBXL4 | KAT5 | NDFIP1 | PRSS12 | SLC35E3 | TRIM3 |  |
| ARL8B | CNIH4 | FBXL5 | KAT6A | NDFIP2 | PRSS21 | SLC35E4 | TRIM31 |  |
| ARMC1 | CNKSR1 | FBXL6 | KAT7 | NDRG1 | PRSS22 | SLC35F2 | TRIM32 |  |
| ARMC10 | CNKSR3 | FBXL8 | KATNA1 | NDRG2 | PRSS23 | SLC35F3 | TRIM33 |  |
| ARMC12 | CNN2 | FBXO10 | KATNAL1 | NDRG3 | PRSS33 | SLC35F6 | TRIM35 |  |
| ARMC2 | CNN3 | FBXO15 | KATNAL2 | NDST1 | PRSS36 | SLC35G2 | TRIM37 |  |
| ARMC6 | CNNM2 | FBXO16 | KATNBL1 | NDST2 | PRSS53 | SLC36A4 | TRIM39 |  |
| ARMC8 | CNNM3 | FBXO2 | KATNIP | NDUFA10 | PRSS54 | SLC37A4 | TRIM41 |  |
| ARMCX3 | CNOT1 | FBXO22 | KAZALD1 | NDUFA11 | PRSS57 | SLC38A1 | TRIM44 |  |
| ARMCX6 | CNOT10 | FBXO24 | KBTBD2 | NDUFA12 | PRSS8 | SLC38A10 | TRIM45 |  |
| ARMH3 | CNOT11 | FBXO25 | KBTBD4 | NDUFA13 | PRTFDC1 | SLC38A11 | TRIM46 |  |
| ARMT1 | CNOT2 | FBXO27 | KBTBD6 | NDUFA2 | PRTG | SLC38A2 | TRIM47 |  |
| ARNT | CNOT3 | FBXO30 | KCMF1 | NDUFA4 | PRUNE1 | SLC38A5 | TRIM50 |  |
| ARNTL | CNOT4 | FBXO31 | KCNA7 | NDUFA4L2 | PRX | SLC38A6 | TRIM52 |  |
| ARNTL2 | CNOT6 | FBXO32 | KCNAB2 | NDUFA5 | PRXL2C | SLC38A9 | TRIM54 |  |
| ARPC1A | CNOT6L | FBXO33 | KCNAB3 | NDUFA6 | PSAP | SLC39A1 | TRIM56 |  |
| ARPC2 | CNOT7 | FBXO34 | KCNC4 | NDUFA7 | PSAPL1 | SLC39A10 | TRIM59 |  |
| ARPC3 | CNOT8 | FBXO36 | KCND1 | NDUFAB1 | PSAT1 | SLC39A11 | TRIM63 |  |
| ARPC4 | CNP | FBXO38 | KCNE3 | NDUFAF1 | PSCA | SLC39A13 | TRIM65 |  |
| ARPC4-TTLL3 | CNPPD1 | FBXO4 | KCNG3 | NDUFAF3 | PSD | SLC39A14 | TRIM66 |  |
| ARPC5 | CNPY2 | FBXO41 | KCNH2 | NDUFAF4 | PSD3 | SLC39A3 | TRIM68 |  |
| ARPIN | CNPY3 | FBXO42 | KCNH5 | NDUFAF5 | PSEN2 | SLC39A4 | TRIM69 |  |
| ARPIN-AP3S2 | CNST | FBXO43 | KCNJ10 | NDUFAF6 | PSENEN | SLC39A6 | TRIM7 |  |
| ARRB2 | CNTFR | FBXO44 | KCNJ12 | NDUFB1 | PSG9 | SLC39A7 | TRIM8 |  |
| ARRDC1 | CNTLN | FBXO45 | KCNK1 | NDUFB10 | PSIP1 | SLC39A8 | TRIML2 |  |
| ARRDC2 | CNTRL | FBXO46 | KCNK15 | NDUFB3 | PSMA1 | SLC3A2 | TRIO |  |
| ARRDC3 | CNTROB | FBXO48 | KCNK5 | NDUFB6 | PSMA3 | SLC41A1 | TRIP12 |  |
| ARRDC5 | COA4 | FBXO5 | KCNK7 | NDUFB7 | PSMA5 | SLC41A2 | TRIP13 |  |
| ARSA | COA5 | FBXO7 | KCNK9 | NDUFB8 | PSMA7 | SLC43A2 | TRIP4 |  |
| ARSB | COA6 | FBXO9 | KCNMB3 | NDUFB9 | PSMB1 | SLC44A1 | TRIP6 |  |
| ARSD | COA7 | FBXW11 | KCNMB4 | NDUFC1 | PSMB10 | SLC44A4 | TRIQK |  |
| ARSI | COA8 | FBXW2 | KCNN4 | NDUFS1 | PSMB2 | SLC45A2 | TRIR |  |
| ARSK | COBL | FBXW4 | KCNQ2 | NDUFS2 | PSMB5 | SLC45A3 | TRIT1 |  |
| ART1 | COBLL1 | FBXW5 | KCP | NDUFS3 | PSMB7 | SLC45A4 | TRMO |  |
| ART5 | COG1 | FBXW8 | KCTD1 | NDUFS4 | PSMB8 | SLC46A1 | TRMT1 |  |
| ARV1 | COG5 | FBXW9 | KCTD10 | NDUFS6 | PSMB9 | SLC46A2 | TRMT10A |  |
| ARVCF | COG6 | FCF1 | KCTD11 | NDUFS7 | PSMC1 | SLC47A2 | TRMT10C |  |
| ASAH2 | COG7 | FCGBP | KCTD13 | NDUFV2 | PSMC3 | SLC48A1 | TRMT11 |  |
| ASAP1 | COG8 | FCGR3A | KCTD14 | NDUFV3 | PSMC3IP | SLC49A3 | TRMT112 |  |
| ASAP2 | COIL | FCGR3B | KCTD15 | NECAB3 | PSMC4 | SLC49A4 | TRMT12 |  |
| ASAP3 | COL11A2 | FCHO1 | KCTD16 | NECAP2 | PSMC6 | SLC4A1 | TRMT13 |  |
| ASB1 | COL12A1 | FCHO2 | KCTD18 | NECTIN3 | PSMD10 | SLC4A1AP | TRMT1L |  |
| ASB11 | COL13A1 | FCHSD1 | KCTD20 | NECTIN4 | PSMD11 | SLC4A7 | TRMT2A |  |
| ASB13 | COL1A1 | FCHSD2 | KCTD3 | NEDD1 | PSMD12 | SLC50A1 | TRMT5 |  |
| ASB2 | COL22A1 | FCMR | KCTD5 | NEDD4 | PSMD13 | SLC51B | TRMT6 |  |
| ASB5 | COL23A1 | FCN1 | KCTD6 | NEDD8 | PSMD3 | SLC5A5 | TRMT61B |  |
| ASB6 | COL25A1 | FCN3 | KCTD7 | NEDD8-MDP1 | PSMD4 | SLC5A6 | TRMU |  |
| ASB7 | COL28A1 | FCSK | KCTD9 | NEDD9 | PSMD5 | SLC66A1 | TRNT1 |  |
| ASB8 | COL2A1 | FDPS | KDELR1 | NEIL1 | PSMD8 | SLC66A2 | TROAP |  |
| ASB9 | COL4A1 | FDX1 | KDM1A | NEIL2 | PSME1 | SLC66A3 | TRPC1 |  |
| ASCC1 | COL4A3 | FDX2 | KDM1B | NEIL3 | PSME2 | SLC6A15 | TRPC3 |  |
| ASCC3 | COL4A4 | FDXACB1 | KDM2A | NEK1 | PSME3IP1 | SLC6A16 | TRPC4AP |  |
| ASCL3 | COL5A1 | FDXR | KDM2B | NEK11 | PSME4 | SLC6A20 | TRPM4 |  |
| ASDURF | COL5A3 | FECH | KDM3A | NEK2 | PSMG1 | SLC6A6 | TRPM7 |  |
| ASF1A | COL6A3 | FEM1A | KDM3B | NEK3 | PSMG2 | SLC6A9 | TRPT1 |  |
| ASF1B | COL7A1 | FEM1B | KDM4A | NEK4 | PSMG3 | SLC7A1 | TRPV4 |  |
| ASGR1 | COLGALT1 | FEM1C | KDM4B | NEK7 | PSORS1C1 | SLC7A10 | TRPV5 |  |
| ASH1L | COLGALT2 | FEN1 | KDM4D | NEK8 | PSPH | SLC7A2 | TRUB1 |  |
| ASH2L | COMMD1 | FER | KDM5A | NEK9 | PSPN | SLC7A4 | TRUB2 |  |
| ASL | COMMD2 | FERMT1 | KDM5B | NELFB | PSRC1 | SLC7A5 | TSACC |  |
| ASMTL | COMMD3 | FERMT2 | KDM5C | NELFCD | PSTPIP2 | SLC7A6 | TSC1 |  |
| ASNS | COMMD3-BMI1 | FEV | KDM6A | NELFE | PTAFR | SLC7A6OS | TSC2 |  |
| ASNSD1 | COMMD4 | FEZ2 | KDM6B | NEMF | PTBP1 | SLC7A7 | TSC22D2 |  |
| ASPDH | COMMD5 | FGD4 | KDM7A | NEMP1 | PTBP2 | SLC8B1 | TSC22D3 |  |
| ASPH | COMMD6 | FGD6 | KDM8 | NEMP2 | PTBP3 | SLC9A1 | TSC22D4 |  |
| ASPHD1 | COMMD7 | FGF1 | KDSR | NENF | PTCD1 | SLC9A2 | TSEN15 |  |
| ASPM | COMMD8 | FGF11 | KEAP1 | NEO1 | PTCD2 | SLC9A6 | TSFM |  |
| ASPN | COMTD1 | FGF18 | KHDC1 | NEPRO | PTCH1 | SLC9A8 | TSG101 |  |
| ASPRV1 | COP1 | FGF19 | KHDC4 | NES | PTDSS1 | SLC9B1 | TSGA10 |  |
| ASS1 | COPA | FGF3 | KHDRBS1 | NETO2 | PTDSS2 | SLC9C2 | TSHR |  |
| ASTE1 | COPB1 | FGFBP3 | KHK | NEU1 | PTEN | SLCO2A1 | TSKS |  |
| ASTL | COPB2 | FGFR1OP2 | KHNYN | NEURL1B | PTER | SLCO3A1 | TSKU |  |
| ASTN1 | COPE | FGFR4 | KHSRP | NEURL2 | PTGER1 | SLCO4A1 | TSN |  |
| ASXL1 | COPG2 | FGFRL1 | KIAA0040 | NEURL3 | PTGER4 | SLCO4C1 | TSNARE1 |  |
| ASXL2 | COPRS | FGGY | KIAA0100 | NEURL4 | PTGES | SLF1 | TSNAX |  |
| ATAD1 | COPS2 | FGL1 | KIAA0319L | NEUROD2 | PTGES2 | SLFN12 | TSNAX-DISC1 | |
| ATAD2 | COPS3 | FGR | KIAA0513 | NF1 | PTGES3 | SLFN13 | TSNAXIP1 |  |
| ATAD2B | COPS4 | FH | KIAA0586 | NF2 | PTGES3L | SLFN14 | TSPAN10 |  |
| ATAD3A | COPS5 | FHDC1 | KIAA0753 | NFAM1 | PTGES3L-AARSD1 | SLFNL1 | TSPAN12 |  |
| ATAD3B | COPS7A | FHIP1A | KIAA0825 | NFAT5 | PTGFR | SLIT1 | TSPAN13 |  |
| ATAD5 | COPS7B | FHIP1B | KIAA0895 | NFATC4 | PTGIR | SLITRK3 | TSPAN14 |  |
| ATE1 | COPS8 | FHIP2A | KIAA0895L | NFE2 | PTGIS | SLITRK5 | TSPAN17 |  |
| ATF2 | COPZ1 | FHIP2B | KIAA0930 | NFE2L1 | PTGR1 | SLK | TSPAN3 |  |
| ATF4 | COPZ2 | FHL2 | KIAA1109 | NFE2L2 | PTGR2 | SLMAP | TSPAN6 |  |
| ATF5 | COQ10A | FHL3 | KIAA1143 | NFKB1 | PTGS2 | SLPI | TSPAN8 |  |
| ATF6 | COQ10B | FHOD1 | KIAA1191 | NFKB2 | PTH1R | SLTM | TSPAN9 |  |
| ATF6B | COQ2 | FIBCD1 | KIAA1328 | NFKBIA | PTH2 | SLURP1 | TSPO |  |
| ATF7 | COQ3 | FIBP | KIAA1549 | NFKBIB | PTK2 | SLX4 | TSPOAP1 |  |
| ATF7IP | COQ4 | FICD | KIAA1671 | NFKBID | PTK6 | SLX4IP | TSPYL2 |  |
| ATF7-NPFF | COQ5 | FIG4 | KIAA1958 | NFKBIE | PTMA | SMAD2 | TSR1 |  |
| ATG10 | COQ6 | FIGN | KIAA2026 | NFS1 | PTMS | SMAD3 | TSR3 |  |
| ATG101 | COQ7 | FIGNL1 | KIDINS220 | NFU1 | PTP4A2 | SMAD4 | TSSC4 |  |
| ATG12 | COQ8A | FIGNL2 | KIF11 | NFX1 | PTP4A3 | SMAD5 | TSSK6 |  |
| ATG13 | COQ8B | FIP1L1 | KIF12 | NFYA | PTPA | SMAD6 | TST |  |
| ATG16L1 | COQ9 | FIS1 | KIF13A | NFYC | PTPDC1 | SMAD7 | TSTD1 |  |
| ATG16L2 | CORO1B | FITM2 | KIF13B | NGEF | PTPN1 | SMAGP | TSTD2 |  |
| ATG2A | CORO1C | FIZ1 | KIF14 | NGF | PTPN11 | SMAP1 | TSTD3 |  |
| ATG2B | CORO2A | FJX1 | KIF15 | NGLY1 | PTPN12 | SMARCA2 | TTBK2 |  |
| ATG3 | CORO6 | FKBP10 | KIF17 | NGRN | PTPN13 | SMARCA4 | TTC13 |  |
| ATG4A | CORO7 | FKBP14 | KIF18A | NHEJ1 | PTPN14 | SMARCAL1 | TTC14 |  |
| ATG4B | CORO7-PAM16 | FKBP1A | KIF18B | NHLRC2 | PTPN18 | SMARCB1 | TTC16 |  |
| ATG4C | COTL1 | FKBP2 | KIF1B | NHLRC3 | PTPN2 | SMARCC1 | TTC17 |  |
| ATG5 | COX10 | FKBP3 | KIF1C | NHLRC4 | PTPN21 | SMARCC2 | TTC19 |  |
| ATG7 | COX11 | FKBP4 | KIF20A | NHP2 | PTPN23 | SMARCD2 | TTC21B |  |
| ATG9A | COX14 | FKBP5 | KIF20B | NHS | PTPN3 | SMARCE1 | TTC22 |  |
| ATG9B | COX15 | FKBP7 | KIF21A | NIBAN1 | PTPN4 | SMC1A | TTC26 |  |
| ATIC | COX17 | FKBP8 | KIF22 | NIBAN2 | PTPN6 | SMC1B | TTC3 |  |
| ATL1 | COX18 | FKBP9 | KIF23 | NICN1 | PTPN7 | SMC2 | TTC30B |  |
| ATL2 | COX19 | FKBPL | KIF24 | NID2 | PTPN9 | SMC3 | TTC31 |  |
| ATL3 | COX20 | FLACC1 | KIF2A | NIFK | PTPRA | SMC5 | TTC32 |  |
| ATM | COX5A | FLAD1 | KIF2C | NINJ1 | PTPRB | SMC6 | TTC33 |  |
| ATN1 | COX5B | FLII | KIF3A | NIP7 | PTPRCAP | SMCHD1 | TTC37 |  |
| ATOH7 | COX6A1 | FLNA | KIF3C | NIPA2 | PTPRE | SMCO1 | TTC39A |  |
| ATOX1 | COX6B2 | FLOT1 | KIF4A | NIPAL1 | PTPRF | SMCO3 | TTC39B |  |
| ATP10D | COX7A1 | FLOT2 | KIF5B | NIPAL3 | PTPRH | SMCR8 | TTC4 |  |
| ATP11A | COX7A2L | FLVCR1 | KIF7 | NIPBL | PTPRJ | SMDT1 | TTC5 |  |
| ATP11B | COX7B2 | FLYWCH1 | KIFAP3 | NIPSNAP1 | PTPRK | SMG1 | TTC6 |  |
| ATP11C | COX7C | FLYWCH2 | KIFBP | NIPSNAP2 | PTPRM | SMG5 | TTC7A |  |
| ATP13A1 | COX8A | FMC1 | KIFC1 | NISCH | PTPRN2 | SMG6 | TTC8 |  |
| ATP13A3 | CP | FMC1-LUC7L2 | KIFC3 | NKAIN1 | PTRH1 | SMG7 | TTC9C |  |
| ATP13A5 | CPA4 | FMN1 | KIN | NKAIN3 | PTRH2 | SMG9 | TTF1 |  |
| ATP1A1 | CPD | FMNL1 | KIRREL1 | NKAIN4 | PTRHD1 | SMIM10 | TTF2 |  |
| ATP1B3 | CPEB1 | FMNL2 | KISS1 | NKAP | PTS | SMIM10L1 | TTLL1 |  |
| ATP23 | CPEB2 | FMNL3 | KITLG | NKG7 | PTTG1IP | SMIM10L2B | TTLL12 |  |
| ATP2A2 | CPLANE1 | FMO4 | KLC1 | NKIRAS1 | PUDP | SMIM12 | TTLL3 |  |
| ATP2A3 | CPLANE2 | FMO5 | KLC2 | NKIRAS2 | PUF60 | SMIM13 | TTLL4 |  |
| ATP2B1 | CPLX1 | FMR1 | KLC4 | NKTR | PUM1 | SMIM14 | TTLL5 |  |
| ATP2C1 | CPM | FN1 | KLF1 | NKX3-2 | PUM2 | SMIM15 | TTLL6 |  |
| ATP5F1A | CPN2 | FN3KRP | KLF10 | NKX6-3 | PUM3 | SMIM19 | TTLL7 |  |
| ATP5F1B | CPNE1 | FNBP1 | KLF11 | NLE1 | PURA | SMIM2 | TTPAL |  |
| ATP5F1C | CPNE2 | FNBP1L | KLF12 | NLK | PURB | SMIM20 | TTYH2 |  |
| ATP5F1E | CPNE3 | FNBP4 | KLF13 | NLN | PURG | SMIM24 | TUBA1B |  |
| ATP5IF1 | CPNE7 | FNDC10 | KLF16 | NLRC3 | PUS1 | SMIM26 | TUBA4A |  |
| ATP5MC1 | CPNE8 | FNDC11 | KLF18 | NLRP1 | PUS10 | SMIM29 | TUBB |  |
| ATP5MC2 | CPOX | FNDC3A | KLF2 | NLRP10 | PUS7 | SMIM40 | TUBB2A |  |
| ATP5MC3 | CPSF1 | FNDC3B | KLF3 | NLRP12 | PUS7L | SMIM42 | TUBB3 |  |
| ATP5ME | CPSF2 | FNDC5 | KLF4 | NLRX1 | PUSL1 | SMIM43 | TUBB4A |  |
| ATP5MF | CPSF3 | FOS | KLF5 | NMB | PVR | SMIM44 | TUBB4B |  |
| ATP5MF-PTCD1 | CPSF4 | FOSB | KLF6 | NMD3 | PWP1 | SMIM6 | TUBB6 |  |
| ATP5MG | CPSF4L | FOSL1 | KLF9 | NME1 | PWWP2A | SMIM7 | TUBD1 |  |
| ATP5MGL | CPSF6 | FOSL2 | KLHDC2 | NME1-NME2 | PWWP2B | SMOC1 | TUBE1 |  |
| ATP5MJ | CPT1A | FOXA1 | KLHDC3 | NME2 | PWWP3A | SMPD1 | TUBG1 |  |
| ATP5PB | CPT1B | FOXD2 | KLHDC9 | NME3 | PXK | SMPD4 | TUBGCP2 |  |
| ATP5PD | CPT2 | FOXF2 | KLHL11 | NME6 | PXMP2 | SMPDL3A | TUBGCP3 |  |
| ATP5PF | CPTP | FOXH1 | KLHL12 | NME7 | PXMP4 | SMPX | TUBGCP4 |  |
| ATP5PO | CPVL | FOXJ3 | KLHL15 | NME9 | PXN | SMS | TUBGCP5 |  |
| ATP6AP2 | CPXM1 | FOXK2 | KLHL17 | NMI | PXT1 | SMU1 | TUBGCP6 |  |
| ATP6V0B | CRABP2 | FOXL2 | KLHL2 | NMNAT1 | PXYLP1 | SMUG1 | TUFM |  |
| ATP6V0D1 | CRACD | FOXL2NB | KLHL20 | NMNAT2 | PYCARD | SMURF1 | TUG1 |  |
| ATP6V0E1 | CRACR2B | FOXM1 | KLHL21 | NMRAL1 | PYCR1 | SMURF2 | TULP2 |  |
| ATP6V1A | CRADD | FOXN2 | KLHL22 | NMRK1 | PYCR2 | SMYD2 | TULP3 |  |
| ATP6V1B2 | CRAMP1 | FOXN4 | KLHL23 | NMT2 | PYCR3 | SMYD4 | TUSC1 |  |
| ATP6V1D | CRAT | FOXO1 | KLHL24 | NNT | PYDC1 | SMYD5 | TUSC2 |  |
| ATP6V1E1 | CREB1 | FOXO3 | KLHL25 | NOC2L | PYDC5 | SNAI1 | TUSC3 |  |
| ATP6V1G1 | CREB3 | FOXO6 | KLHL26 | NOC3L | PYGB | SNAI2 | TUT1 |  |
| ATP6V1G2 | CREB3L2 | FOXP2 | KLHL28 | NOC4L | PYGL | SNAI3 | TUT4 |  |
| ATP6V1G2-DDX39B | CREBBP | FOXQ1 | KLHL32 | NOCT | PYGM | SNAP25 | TUT7 |  |
| ATP7A | CREBL2 | FOXRED1 | KLHL33 | NOD1 | PYGO2 | SNAP29 | TVP23A |  |
| ATP7B | CREBRF | FOXRED2 | KLHL35 | NOG | PYM1 | SNAPC3 | TWF1 |  |
| ATP8B2 | CREBZF | FOXS1 | KLHL38 | NOL10 | PYROXD2 | SNAPC4 | TWF2 |  |
| ATP8B3 | CREG1 | FPGS | KLHL42 | NOL11 | PYURF | SNAPC5 | TWSG1 |  |
| ATP8B4 | CREG2 | FPGT | KLHL5 | NOL12 | QARS1 | SNAPIN | TXLNA |  |
| ATP9A | CRELD1 | FPGT-TNNI3K | KLHL7 | NOL7 | QDPR | SNCB | TXLNG |  |
| ATP9B | CRELD2 | FRAS1 | KLHL8 | NOL9 | QPCTL | SND1 | TXN |  |
| ATPSCKMT | CREM | FRAT1 | KLHL9 | NOLC1 | QRFP | SNF8 | TXN2 |  |
| ATR | CRH | FRAT2 | KLK14 | NOM1 | QRICH1 | SNRK | TXNDC11 |  |
| ATRAID | CRIP2 | FRG1 | KLK5 | NOP10 | QRSL1 | SNRNP200 | TXNDC12 |  |
| ATRIP | CRIPT | FRK | KLLN | NOP14 | QSER1 | SNRNP25 | TXNDC15 |  |
| ATXN10 | CRISPLD2 | FRRS1 | KLRC2 | NOP16 | QSOX2 | SNRNP35 | TXNDC16 |  |
| ATXN1L | CRK | FRRS1L | KLRC3 | NOP2 | QTRT2 | SNRNP40 | TXNDC17 |  |
| ATXN2 | CRKL | FRS2 | KLRC4 | NOP56 | R3HCC1 | SNRNP70 | TXNDC5 |  |
| ATXN2L | CRLF1 | FRS3 | KLRC4-KLRK1 | NOP9 | R3HCC1L | SNRPA1 | TXNDC8 |  |
| ATXN3 | CRLF2 | FRYL | KLRK1 | NOPCHAP1 | R3HDM1 | SNRPB | TXNDC9 |  |
| ATXN7 | CRLF3 | FSCN1 | KMT2A | NOSIP | R3HDM2 | SNRPB2 | TXNIP |  |
| ATXN7L1 | CRLS1 | FSCN2 | KMT2B | NOTCH1 | R3HDM4 | SNRPC | TXNL1 |  |
| ATXN7L3 | CRNKL1 | FSD1 | KMT2C | NOTCH3 | RAB10 | SNRPD1 | TXNL4A |  |
| AUNIP | CRTAP | FSD2 | KMT2D | NOTUM | RAB11A | SNRPD2 | TXNL4B |  |
| AUP1 | CRTC2 | FSIP1 | KMT2E | NOVA2 | RAB11FIP1 | SNRPE | TXNRD2 |  |
| AURKA | CRTC3 | FSTL3 | KMT5B | NOXA1 | RAB11FIP2 | SNRPG | TXNRD3 |  |
| AURKAIP1 | CRY1 | FSTL4 | KMT5C | NOXO1 | RAB11FIP3 | SNTA1 | TYK2 |  |
| AURKB | CRY2 | FTCDNL1 | KNDC1 | NPAS2 | RAB11FIP5 | SNTB2 | TYMP |  |
| AVEN | CRYAB | FTH1 | KNL1 | NPAS4 | RAB13 | SNUPN | TYMS |  |
| AVIL | CRYBA2 | FTL | KNSTRN | NPAT | RAB14 | SNW1 | TYROBP |  |
| AVL9 | CRYBG1 | FTSJ1 | KNTC1 | NPBWR2 | RAB17 | SNX12 | TYSND1 |  |
| AVPI1 | CRYBG2 | FTSJ3 | KPNA1 | NPC1 | RAB1A | SNX13 | TYW3 |  |
| AXDND1 | CRYBG3 | FUBP1 | KPNA2 | NPC2 | RAB20 | SNX14 | TYW5 |  |
| AXIN1 | CRYGN | FUBP3 | KPNA4 | NPDC1 | RAB21 | SNX15 | U2AF1L4 |  |
| AXIN2 | CRYGS | FUCA1 | KPNA5 | NPFF | RAB22A | SNX17 | U2AF2 |  |
| AZI2 | CRYL1 | FUCA2 | KPNA6 | NPFFR1 | RAB24 | SNX19 | U2SURP |  |
| AZIN1 | CRYM | FUNDC1 | KPNB1 | NPHP1 | RAB26 | SNX20 | UACA |  |
| B2M | CRYZ | FUNDC2 | KRAS | NPHP3 | RAB27A | SNX24 | UAP1 |  |
| B3GALNT2 | CRYZL1 | FURIN | KRBA2 | NPHP3-ACAD11 | RAB27B | SNX25 | UAP1L1 |  |
| B3GALT6 | CS | FUS | KRBOX4 | NPHP4 | RAB28 | SNX3 | UBA1 |  |
| B3GALT9 | CSAD | FUT1 | KREMEN2 | NPHS1 | RAB29 | SNX31 | UBA2 |  |
| B3GAT3 | CSDE1 | FUT10 | KRI1 | NPIPB7 | RAB2A | SNX33 | UBA3 |  |
| B3GNT2 | CSE1L | FUT11 | KRIT1 | NPL | RAB2B | SNX4 | UBA52 |  |
| B3GNT5 | CSF1R | FUT4 | KRR1 | NPLOC4 | RAB30 | SNX5 | UBA6 |  |
| B3GNT9 | CSF3R | FUT7 | KRT1 | NPPA | RAB31 | SNX6 | UBAC1 |  |
| B3GNTL1 | CSGALNACT1 | FUT8 | KRT10 | NPPB | RAB32 | SNX7 | UBAC2 |  |
| B4GALNT1 | CSGALNACT2 | FXN | KRT13 | NPR1 | RAB33B | SNX8 | UBALD1 |  |
| B4GALT1 | CSK | FXR1 | KRT14 | NPR3 | RAB34 | SNX9 | UBAP1 |  |
| B4GALT2 | CSKMT | FXR2 | KRT15 | NPRL2 | RAB39A | SOAT1 | UBAP1L |  |
| B4GALT3 | CSNK1A1 | FXYD2 | KRT16 | NPRL3 | RAB3A | SOCS1 | UBAP2 |  |
| B4GALT5 | CSNK1A1L | FXYD3 | KRT17 | NPTN | RAB3D | SOCS2 | UBAP2L |  |
| B4GALT6 | CSNK1D | FXYD5 | KRT18 | NPTX1 | RAB3GAP2 | SOCS3 | UBB |  |
| B4GAT1 | CSNK1E | FYB1 | KRT19 | NPTXR | RAB3IL1 | SOCS4 | UBC |  |
| B9D1 | CSNK1G1 | FYB2 | KRT2 | NQO1 | RAB3IP | SOCS5 | UBE2B |  |
| BABAM2 | CSNK1G2 | FYCO1 | KRT20 | NQO2 | RAB40B | SOCS6 | UBE2C |  |
| BACH1 | CSNK1G3 | FYTTD1 | KRT24 | NR0B1 | RAB4B | SOCS7 | UBE2D1 |  |
| BAD | CSNK2A1 | FZD10 | KRT25 | NR0B2 | RAB4B-EGLN2 | SOD1 | UBE2D3 |  |
| BAG1 | CSNK2A2 | FZD2 | KRT26 | NR1D1 | RAB5A | SOD2 | UBE2D4 |  |
| BAG2 | CSNK2B | FZD3 | KRT27 | NR1D2 | RAB5B | SOGA1 | UBE2E3 |  |
| BAG3 | CSPG4 | FZD4 | KRT28 | NR1H3 | RAB5C | SON | UBE2F |  |
| BAG4 | CSRNP1 | FZD5 | KRT3 | NR2C1 | RAB5IF | SORBS1 | UBE2F-SCLY | |
| BAG5 | CSRNP2 | FZD6 | KRT31 | NR2C2 | RAB6A | SORBS3 | UBE2G1 |  |
| BAG6 | CSRP1 | FZD7 | KRT32 | NR2C2AP | RAB7B | SORD | UBE2G2 |  |
| BAHD1 | CSRP2 | FZD9 | KRT34 | NR2F6 | RAB8A | SORT1 | UBE2H |  |
| BAIAP2L1 | CST3 | FZR1 | KRT35 | NR4A2 | RAB8B | SOS1 | UBE2I |  |
| BAIAP2L2 | CST6 | G3BP1 | KRT36 | NR4A3 | RABAC1 | SOS2 | UBE2J1 |  |
| BAK1 | CST9 | G3BP2 | KRT37 | NR6A1 | RABEP1 | SOST | UBE2J2 |  |
| BAMBI | CSTB | G6PC3 | KRT38 | NRAP | RABEP2 | SOWAHC | UBE2L5 |  |
| BANF1 | CSTF1 | G6PD | KRT5 | NRARP | RABEPK | SOX10 | UBE2M |  |
| BANP | CSTF2 | GAA | KRT6A | NRAS | RABGAP1L | SOX12 | UBE2N |  |
| BARD1 | CSTF2T | GAB1 | KRT76 | NRBF2 | RABGEF1 | SOX15 | UBE2O |  |
| BARX1 | CSTF3 | GAB2 | KRT78 | NRBP1 | RABGGTA | SOX17 | UBE2Q1 |  |
| BASP1 | CTAGE1 | GAB3 | KRT8 | NRBP2 | RABGGTB | SOX18 | UBE2Q2 |  |
| BATF2 | CTBP1 | GABARAP | KRT80 | NRDC | RABIF | SOX7 | UBE2R2 |  |
| BATF3 | CTBP2 | GABARAPL1 | KRT83 | NRDE2 | RABL6 | SP1 | UBE2S |  |
| BAX | CTBS | GABARAPL2 | KRT9 | NREP | RAC1 | SP110 | UBE2T |  |
| BAZ1B | CTC1 | GABBR1 | KRTCAP2 | NRF1 | RACK1 | SP2 | UBE2V1 |  |
| BAZ2A | CTCF | GABPA | KRTDAP | NRIP2 | RAD1 | SP3 | UBE2V2 |  |
| BAZ2B | CTDNEP1 | GABPB1 | KSR1 | NRL | RAD17 | SP4 | UBE2W |  |
| BBC3 | CTDP1 | GABPB2 | KTI12 | NRM | RAD18 | SP6 | UBE3B |  |
| BBIP1 | CTDSP1 | GABRE | KTN1 | NRN1L | RAD21 | SP7 | UBE3C |  |
| BBLN | CTDSP2 | GADD45A | KXD1 | NRP1 | RAD23A | SP8 | UBE3D |  |
| BBOF1 | CTDSPL2 | GADD45B | KYAT1 | NRTN | RAD23B | SPACA5 | UBE4A |  |
| BBS1 | CTH | GADD45GIP1 | KYAT3 | NRXN2 | RAD50 | SPACA9 | UBE4B |  |
| BBS10 | CTIF | GAK | L1CAM | NSA2 | RAD51 | SPAG1 | UBIAD1 |  |
| BBS12 | CTNNA1 | GAL | L2HGDH | NSD1 | RAD51D | SPAG16 | UBL4A |  |
| BBS2 | CTNNAL1 | GAL3ST1 | L3HYPDH | NSD2 | RAD52 | SPAG4 | UBL5 |  |
| BBS4 | CTNNBL1 | GAL3ST3 | L3MBTL1 | NSD3 | RAD54B | SPAG5 | UBL7 |  |
| BBS7 | CTPS2 | GAL3ST4 | L3MBTL2 | NSFL1C | RAD54L2 | SPAG7 | UBN1 |  |
| BBX | CTR9 | GALE | LACC1 | NSL1 | RAD9A | SPAG8 | UBOX5 |  |
| BCAP29 | CTRL | GALK1 | LACTB | NSMAF | RAD9B | SPAG9 | UBP1 |  |
| BCAP31 | CTSA | GALK2 | LACTB2 | NSMCE1 | RAE1 | SPARC | UBQLN1 |  |
| BCAR1 | CTSB | GALM | LACTBL1 | NSMCE2 | RAET1G | SPART | UBQLN4 |  |
| BCAR3 | CTSC | GALNT1 | LAGE3 | NSMCE3 | RAET1L | SPAST | UBR1 |  |
| BCAS3 | CTSD | GALNT10 | LAMA1 | NSMCE4A | RAF1 | SPATA1 | UBR2 |  |
| BCAS4 | CTSE | GALNT11 | LAMA3 | NSMF | RAG1 | SPATA12 | UBR3 |  |
| BCAT2 | CTSF | GALNT2 | LAMA5 | NSUN2 | RAI1 | SPATA17 | UBR5 |  |
| BCCIP | CTSG | GALNT3 | LAMB1 | NSUN3 | RALA | SPATA2 | UBR7 |  |
| BCDIN3D | CTSV | GALNT4 | LAMB2 | NSUN4 | RALB | SPATA20 | UBTD1 |  |
| BCKDHA | CTSZ | GALNT6 | LAMB3 | NSUN5 | RALBP1 | SPATA21 | UBTD2 |  |
| BCKDHB | CTTN | GALNT7 | LAMB4 | NSUN6 | RALGAPA1 | SPATA24 | UBTF |  |
| BCKDK | CTTNBP2NL | GALNT9 | LAMC1 | NT5C | RALGAPA2 | SPATA25 | UBXN1 |  |
| BCL10 | CTU1 | GALT | LAMP1 | NT5C1A | RALGAPB | SPATA2L | UBXN11 |  |
| BCL11A | CTU2 | GAMT | LAMP2 | NT5C2 | RALGDS | SPATA32 | UBXN2A |  |
| BCL11B | CTXN1 | GAN | LAMTOR1 | NT5C3A | RALGPS1 | SPATA33 | UBXN2B |  |
| BCL2L1 | CUEDC2 | GANAB | LAMTOR2 | NT5C3B | RALY | SPATA45 | UBXN4 |  |
| BCL2L10 | CUL1 | GANC | LAMTOR5 | NT5DC1 | RAN | SPATA46 | UBXN6 |  |
| BCL2L12 | CUL4A | GAPDH | LANCL2 | NT5DC3 | RANBP1 | SPATA5 | UBXN7 |  |
| BCL2L15 | CUL7 | GAPVD1 | LAP3 | NT5E | RANBP10 | SPATA5L1 | UBXN8 |  |
| BCL2L2 | CUTA | GAR1 | LAPTM4A | NT5M | RANBP2 | SPATA6L | UCHL3 |  |
| BCL3 | CUTC | GAREM1 | LAPTM4B | NTAN1 | RANBP3 | SPATA9 | UCHL5 |  |
| BCL7B | CUZD1 | GARIN3 | LAPTM5 | NTAQ1 | RANBP6 | SPATC1L | UCK1 |  |
| BCL7C | CWC15 | GARIN5A | LARP1 | NTF4 | RANBP9 | SPATS2 | UCK2 |  |
| BCL9L | CWC25 | GARIN5B | LARP1B | NTHL1 | RANGAP1 | SPC24 | UCKL1 |  |
| BCLAF1 | CWC27 | GARRE1 | LARP4 | NTN4 | RANGRF | SPCS1 | UCN |  |
| BCLAF3 | CWF19L1 | GART | LARP6 | NTN5 | RAP1B | SPCS2 | UCN2 |  |
| BCOR | CXADR | GAS1 | LARP7 | NTPCR | RAP1GAP | SPCS3 | UCP2 |  |
| BCS1L | CXCL10 | GAS2L1 | LARS1 | NTRK1 | RAP1GDS1 | SPDEF | UEVLD |  |
| BDH1 | CXCL11 | GAS2L2 | LARS2 | NTSR2 | RAP2A | SPDL1 | UFD1 |  |
| BDKRB1 | CXCL14 | GAS2L3 | LAS1L | NUAK1 | RAP2B | SPDYC | UFL1 |  |
| BDKRB2 | CXCL16 | GAS6 | LASP1 | NUAK2 | RAPGEF1 | SPDYE16 | UFSP1 |  |
| BECN1 | CXCL2 | GATA2 | LATS1 | NUBP2 | RAPGEF3 | SPDYE6 | UGCG |  |
| BEGAIN | CXCL8 | GATA4 | LATS2 | NUBPL | RAPH1 | SPECC1 | UGDH |  |
| BEND3 | CXCR1 | GATA5 | LBHD1 | NUCB2 | RAPSN | SPECC1L | UGP2 |  |
| BEND6 | CXCR4 | GATA6 | LBR | NUCKS1 | RARA | SPECC1L-ADORA2A | UGT1A6 |  |
| BEST4 | CXorf38 | GATAD1 | LBX2 | NUDCD1 | RARG | SPEF1 | UHMK1 |  |
| BET1L | CXorf58 | GATAD2A | LCA5 | NUDCD3 | RARRES1 | SPEF2 | UHRF1BP1 | |
| BFAR | CXorf65 | GATAD2B | LCAT | NUDT12 | RARS2 | SPG11 | UIMC1 |  |
| BFSP1 | CXXC1 | GATC | LCLAT1 | NUDT13 | RASA1 | SPG7 | ULBP3 |  |
| BHLHE40 | CYB561 | GATM | LCMT2 | NUDT14 | RASA2 | SPHK2 | ULK2 |  |
| BICD1 | CYB561A3 | GBA | LCN10 | NUDT15 | RASA3 | SPI1 | ULK3 |  |
| BICD2 | CYB561D1 | GBA2 | LCN15 | NUDT16L1 | RASAL1 | SPICE1 | ULK4 |  |
| BICDL1 | CYB561D2 | GBE1 | LCN2 | NUDT18 | RASAL2 | SPIDR | UMAD1 |  |
| BICDL2 | CYB5D1 | GBGT1 | LCN6 | NUDT19 | RASD1 | SPIN1 | UMPS |  |
| BICRA | CYB5R1 | GBX2 | LCN8 | NUDT2 | RASEF | SPIN3 | UNC119 |  |
| BICRAL | CYB5R2 | GCA | LCNL1 | NUDT21 | RASGEF1C | SPINDOC | UNC119B |  |
| BIN1 | CYB5R3 | GCC1 | LCOR | NUDT22 | RASGRP2 | SPINT3 | UNC13D |  |
| BIN2 | CYB5R4 | GCC2 | LCORL | NUDT3 | RASIP1 | SPIRE1 | UNC45A |  |
| BIN3 | CYB5RL | GCFC2 | LDAF1 | NUDT6 | RASL10A | SPIRE2 | UNC50 |  |
| BIRC2 | CYBA | GCH1 | LDAH | NUDT7 | RASL11A | SPNS1 | UNC5B |  |
| BIRC3 | CYBC1 | GCHFR | LDB1 | NUDT8 | RASL12 | SPOCK2 | UNC93B1 |  |
| BIRC5 | CYC1 | GCLM | LDHA | NUDT9 | RASSF1 | SPON2 | UNG |  |
| BIRC6 | CYCS | GCM1 | LDHB | NUF2 | RASSF8 | SPOP | UNKL |  |
| BIRC7 | CYFIP1 | GCN1 | LDHD | NUFIP2 | RAVER1 | SPOPL | UPF1 |  |
| BIVM | CYGB | GCNT4 | LDLR | NUGGC | RAVER2 | SPOUT1 | UPF3A |  |
| BLACAT1 | CYHR1 | GCNT7 | LDLRAD3 | NUMA1 | RAX2 | SPPL2A | UPF3B |  |
| BLM | CYLD | GCOM1 | LEFTY1 | NUMB | RB1 | SPPL3 | UPK3B |  |
| BLMH | CYP11A1 | GCSAM | LEKR1 | NUMBL | RB1CC1 | SPR | UPP1 |  |
| BLOC1S1 | CYP11B2 | GCSH | LEMD2 | NUP107 | RBAK | SPRED1 | UPRT |  |
| BLOC1S2 | CYP1A1 | GDAP1 | LEMD3 | NUP133 | RBBP4 | SPRED2 | UQCR10 |  |
| BLOC1S4 | CYP1B1 | GDAP2 | LENG8 | NUP153 | RBBP5 | SPRED3 | UQCR11 |  |
| BLOC1S5 | CYP20A1 | GDE1 | LENG9 | NUP155 | RBBP6 | SPRING1 | UQCRB |  |
| BLOC1S5-TXNDC5 | CYP27B1 | GDF1 | LEO1 | NUP160 | RBBP7 | SPRR2E | UQCRC1 |  |
| BLOC1S6 | CYP27C1 | GDF11 | LEPR | NUP188 | RBBP8 | SPRR2F | UQCRC2 |  |
| BLVRA | CYP2D7 | GDF15 | LEPROT | NUP210 | RBBP8NL | SPRY2 | UQCRH |  |
| BLVRB | CYP2R1 | GDF5 | LEPROTL1 | NUP210L | RBCK1 | SPRY3 | UQCRHL |  |
| BLZF1 | CYP2S1 | GDF9 | LETM1 | NUP214 | RBIS | SPRYD3 | UQCRQ |  |
| BMF | CYP3A43 | GDPD1 | LETMD1 | NUP37 | RBKS | SPRYD4 | URB1 |  |
| BMP1 | CYP3A7 | GDPD3 | LGALS1 | NUP42 | RBL1 | SPRYD7 | URB2 |  |
| BMP2 | CYP3A7-CYP3A51P | GDPGP1 | LGALS2 | NUP43 | RBL2 | SPSB2 | URGCP |  |
| BMP2K | CYP46A1 | GEM | LGALS3BP | NUP54 | RBM12 | SPSB3 | URGCP-MRPS24 | |
| BMP8B | CYP4F11 | GEMIN2 | LGALS4 | NUP58 | RBM12B | SPTAN1 | URM1 |  |
| BMPR1A | CYP4F3 | GEMIN4 | LGALSL | NUP62 | RBM14 | SPTB | UROC1 |  |
| BMPR2 | CYP4V2 | GEMIN5 | LGI3 | NUP85 | RBM14-RBM4 | SPTBN2 | UROS |  |
| BMS1 | CYP51A1 | GEMIN6 | LGI4 | NUP88 | RBM15 | SPTBN4 | USB1 |  |
| BMT2 | CYP7A1 | GEMIN7 | LGMN | NUP98 | RBM15B | SPTLC2 | USE1 |  |
| BNC1 | CYP8B1 | GEMIN8 | LGR4 | NUPR1 | RBM17 | SPTSSA | USF3 |  |
| BNIP2 | CYREN | GEN1 | LGR6 | NUTF2 | RBM18 | SPTY2D1 | USH1C |  |
| BNIP3 | CYRIB | GET4 | LHFPL2 | NUTM1 | RBM19 | SQLE | USH1G |  |
| BNIP3L | CYS1 | GFAP | LHFPL7 | NUTM2E | RBM22 | SQSTM1 | USHBP1 |  |
| BNIP5 | CYSRT1 | GFER | LHX2 | NVL | RBM25 | SRA1 | USP1 |  |
| BOD1 | CYSTM1 | GFI1B | LHX3 | NWD1 | RBM26 | SRCAP | USP10 |  |
| BOD1L1 | CYTH2 | GFM1 | LHX4 | NXF1 | RBM27 | SRCIN1 | USP11 |  |
| BOLA1 | CYTH3 | GFM2 | LIAS | NXN | RBM28 | SRD5A1 | USP12 |  |
| BOLA2 | CZIB | GFOD1 | LIF | NXNL1 | RBM34 | SREBF1 | USP13 |  |
| BOLA2-SMG1P6 | D2HGDH | GFPT1 | LIFR | NXNL2 | RBM38 | SREBF2 | USP14 |  |
| BOLA3 | DAAM1 | GFUS | LIG1 | NXPH4 | RBM39 | SREK1 | USP15 |  |
| BOP1 | DAB2 | GGA1 | LIG3 | NXT1 | RBM4 | SREK1IP1 | USP18 |  |
| BORA | DACT3 | GGA3 | LIG4 | OARD1 | RBM42 | SRF | USP19 |  |
| BORCS5 | DAD1 | GGCT | LIM2 | OAS3 | RBM43 | SRGAP1 | USP2 |  |
| BORCS6 | DAG1 | GGCX | LIMA1 | OASL | RBM45 | SRI | USP20 |  |
| BORCS8 | DAGLA | GGH | LIMCH1 | OAT | RBM47 | SRL | USP21 |  |
| BORCS8-MEF2B | DAGLB | GGN | LIMD1 | OAZ3 | RBM4B | SRM | USP22 |  |
| BPGM | DALRD3 | GGNBP2 | LIMK2 | OBI1 | RBM5 | SRMS | USP24 |  |
| BPHL | DAND5 | GGT5 | LIMS1 | OBP2B | RBM6 | SRP14 | USP25 |  |
| BPIFC | DAO | GGT7 | LIMS2 | OBSL1 | RBM7 | SRP54 | USP28 |  |
| BPNT1 | DAOA | GGTA1 | LIN52 | OCEL1 | RBM8A | SRP72 | USP3 |  |
| BPTF | DAP | GH1 | LIN54 | OCIAD1 | RBMS1 | SRP9 | USP31 |  |
| BRAF | DAP3 | GHITM | LIN7B | OCIAD2 | RBMXL1 | SRPK1 | USP32 |  |
| BRAT1 | DAPK2 | GHRH | LIN9 | ODAD1 | RBPJ | SRPK2 | USP33 |  |
| BRCA1 | DAPK3 | GHRL | LINGO4 | ODAD3 | RBPMS2 | SRPRA | USP36 |  |
| BRCA2 | DAPP1 | GID8 | LINS1 | ODC1 | RBSN | SRPX | USP38 |  |
| BRCC3 | DARS2 | GIGYF1 | LIPT1 | ODF2 | RC3H1 | SRR | USP4 |  |
| BRD1 | DAXX | GIN1 | LIPT2 | ODF2L | RC3H2 | SRRD | USP40 |  |
| BRD2 | DAZAP1 | GINS1 | LITAF | ODF3B | RCAN1 | SRRM1 | USP42 |  |
| BRD3 | DAZAP2 | GINS4 | LIX1L | ODF3L2 | RCAN3 | SRRM2 | USP43 |  |
| BRD7 | DBF4 | GIP | LKAAEAR1 | ODR4 | RCBTB1 | SRRM3 | USP45 |  |
| BRD8 | DBF4B | GIPC1 | LLGL2 | OFD1 | RCBTB2 | SRRM5 | USP47 |  |
| BRD9 | DBI | GIT1 | LMAN1 | OGA | RCC1L | SRRT | USP48 |  |
| BRF2 | DBN1 | GIT2 | LMAN2 | OGDH | RCC2 | SRSF1 | USP49 |  |
| BRI3BP | DBNDD1 | GJA3 | LMAN2L | OGFOD1 | RCCD1 | SRSF10 | USP53 |  |
| BRICD5 | DBNL | GJA9 | LMBR1 | OGFOD3 | RCE1 | SRSF11 | USP54 |  |
| BRIP1 | DBP | GJB3 | LMBR1L | OGFRL1 | RCHY1 | SRSF12 | USP7 |  |
| BRIX1 | DBR1 | GJB5 | LMBRD1 | OGG1 | RCL1 | SRSF2 | USP8 |  |
| BRK1 | DBT | GJC2 | LMBRD2 | OGT | RCN1 | SRSF3 | USP9X |  |
| BRME1 | DCAF1 | GJD3 | LMF1 | OIP5 | RCN3 | SRSF4 | USPL1 |  |
| BRMS1 | DCAF10 | GK | LMF2 | OLA1 | RCOR2 | SRSF5 | UST |  |
| BRMS1L | DCAF11 | GK5 | LMLN2 | OLFM1 | RCOR3 | SRSF7 | UTP11 |  |
| BROX | DCAF13 | GKAP1 | LMNB1 | OLR1 | RCVRN | SRSF8 | UTP15 |  |
| BRWD1 | DCAF15 | GLA | LMNB2 | OMA1 | RD3 | SRSF9 | UTP18 |  |
| BSCL2 | DCAF16 | GLB1 | LMNTD2 | OOEP | RDH11 | SRXN1 | UTP23 |  |
| BSDC1 | DCAF17 | GLB1L | LMOD1 | OOSP3 | RDH13 | SS18 | UTP3 |  |
| BSG | DCAF4 | GLCCI1 | LMOD3 | OPA1 | RDH14 | SS18L1 | UTP4 |  |
| BSN | DCAF5 | GLE1 | LMTK3 | OPA3 | RDH16 | SS18L2 | UTP6 |  |
| BST2 | DCAF6 | GLG1 | LMX1A | OPALIN | RDH5 | SSB | UTRN |  |
| BTAF1 | DCAF7 | GLIPR1L2 | LNP1 | OPHN1 | RDX | SSBP1 | UTS2B |  |
| BTBD1 | DCAKD | GLIPR2 | LNPEP | OPLAH | RECQL4 | SSBP2 | UVRAG |  |
| BTBD10 | DCANP1 | GLIS1 | LNPK | OPN3 | RECQL5 | SSBP3 | UXS1 |  |
| BTBD17 | DCBLD1 | GLIS2 | LNX2 | OPTN | REEP1 | SSBP4 | UXT |  |
| BTBD18 | DCBLD2 | GLMN | LONP1 | OR10H1 | REEP3 | SSC4D | VAC14 |  |
| BTBD19 | DCHS1 | GLMP | LONP2 | OR10V1 | REEP4 | SSC5D | VAMP1 |  |
| BTBD2 | DCK | GLO1 | LONRF1 | OR13J1 | REEP5 | SSH1 | VAMP2 |  |
| BTBD3 | DCLK1 | GLOD4 | LONRF2 | OR1J1 | REL | SSH2 | VANGL1 |  |
| BTBD8 | DCLK2 | GLOD5 | LOXL2 | OR2AE1 | RELA | SSH3 | VAPA |  |
| BTC | DCLK3 | GLRA1 | LOXL4 | OR2B2 | RELB | SSMEM1 | VAPB |  |
| BTD | DCLRE1A | GLRX | LPAR2 | OR2V1 | RELCH | SSNA1 | VARS1 |  |
| BTF3 | DCLRE1B | GLRX2 | LPCAT1 | OR52L1 | RELL1 | SSR1 | VARS2 |  |
| BTF3L4 | DCLRE1C | GLS | LPCAT2 | OR56A5 | RELL2 | SSR2 | VASP |  |
| BTG1 | DCN | GLT8D1 | LPCAT4 | OR6B2 | RELT | SSR3 | VAT1 |  |
| BTG3 | DCP1A | GLT8D2 | LPGAT1 | OR6B3 | RENBP | SSR4 | VAV2 |  |
| BTK | DCP1B | GLTP | LPIN1 | OR6C70 | REPS1 | SSRP1 | VAX1 |  |
| BTN1A1 | DCPS | GLUD1 | LPIN2 | OR6J1 | REPS2 | SSU72 | VCL |  |
| BTN2A1 | DCST2 | GLYR1 | LPIN3 | ORAI1 | RER1 | SSUH2 | VCP |  |
| BTN3A1 | DCTD | GMCL1 | LPO | ORAI3 | RERE | SSX2IP | VCPIP1 |  |
| BTRC | DCTN1 | GMCL2 | LPP | ORC2 | RESF1 | ST13 | VCPKMT |  |
| BUB1B | DCTN3 | GMEB2 | LPXN | ORC3 | REST | ST20-MTHFS | VCX2 |  |
| BYSL | DCTN5 | GMFB | LRATD2 | ORC4 | RETREG1 | ST3GAL1 | VDAC1 |  |
| BZW1 | DCTPP1 | GMFG | LRBA | ORC5 | RETREG2 | ST3GAL2 | VDAC2 |  |
| BZW2 | DCUN1D2 | GMIP | LRCH3 | ORC6 | RETREG3 | ST3GAL3 | VDAC3 |  |
| C10orf105 | DCUN1D3 | GMNN | LRCH4 | ORMDL1 | RETSAT | ST3GAL5 | VEGFA |  |
| C10orf143 | DCUN1D4 | GMPPA | LRFN1 | ORMDL2 | REV1 | ST6GALNAC1 | VEGFB |  |
| C10orf67 | DCUN1D5 | GMPPB | LRFN4 | ORMDL3 | REV3L | ST6GALNAC2 | VEZF1 |  |
| C10orf88 | DCXR | GMPR2 | LRG1 | OSBP | REX1BD | ST6GALNAC4 | VEZT |  |
| C10orf95 | DDAH1 | GMPS | LRGUK | OSBPL10 | REXO1 | ST6GALNAC6 | VGF |  |
| C11orf1 | DDAH2 | GNA12 | LRIF1 | OSBPL11 | REXO4 | ST7 | VGLL3 |  |
| C11orf24 | DDB1 | GNA13 | LRIG2 | OSBPL1A | REXO5 | ST7L | VHL |  |
| C11orf54 | DDB2 | GNAI3 | LRIG3 | OSBPL2 | RFC1 | ST8SIA6 | VHLL |  |
| C11orf58 | DDHD1 | GNAT2 | LRMDA | OSBPL3 | RFC2 | STAB1 | VIM |  |
| C11orf68 | DDHD2 | GNAZ | LRP10 | OSBPL5 | RFC4 | STAC | VIPR1 |  |
| C11orf71 | DDI2 | GNB1 | LRP11 | OSBPL6 | RFESD | STAC2 | VIRMA |  |
| C11orf80 | DDIAS | GNB1L | LRP12 | OSBPL7 | RFFL | STAC3 | VKORC1 |  |
| C11orf86 | DDIT4 | GNB2 | LRP1B | OSBPL8 | RFK | STAG1 | VMA21 |  |
| C11orf91 | DDRGK1 | GNB4 | LRP2BP | OSCP1 | RFTN1 | STAG2 | VMAC |  |
| C11orf94 | DDT | GNB5 | LRP3 | OSER1 | RFWD3 | STAM | VMO1 |  |
| C11orf98 | DDX1 | GNE | LRP5 | OSGEP | RFX1 | STAM2 | VMP1 |  |
| C12orf29 | DDX10 | GNG11 | LRP6 | OSGEPL1 | RFX3 | STAMBPL1 | VOPP1 |  |
| C12orf50 | DDX11 | GNG12 | LRPPRC | OSGIN2 | RFX5 | STAP2 | VPREB3 |  |
| C12orf56 | DDX17 | GNG13 | LRR1 | OSM | RFX7 | STAR | VPS11 |  |
| C12orf57 | DDX18 | GNG5 | LRRC1 | OSMR | RFXAP | STARD10 | VPS13A |  |
| C12orf71 | DDX19A | GNG7 | LRRC20 | OSR2 | RGL1 | STARD13 | VPS13B |  |
| C12orf73 | DDX19B | GNG8 | LRRC23 | OST4 | RGL2 | STARD3NL | VPS13C |  |
| C12orf75 | DDX20 | GNGT2 | LRRC24 | OSTC | RGL3 | STARD4 | VPS13D |  |
| C12orf76 | DDX21 | GNL1 | LRRC25 | OSTF1 | RGP1 | STARD5 | VPS16 |  |
| C13orf46 | DDX23 | GNL2 | LRRC26 | OSTM1 | RGS10 | STARD6 | VPS18 |  |
| C14orf119 | DDX24 | GNL3 | LRRC27 | OTOF | RGS16 | STARD7 | VPS25 |  |
| C14orf28 | DDX27 | GNPAT | LRRC37A3 | OTOG | RGS19 | STARD9 | VPS26A |  |
| C14orf93 | DDX28 | GNPDA1 | LRRC37B | OTUB1 | RGS2 | STAT1 | VPS26B |  |
| C15orf40 | DDX31 | GNPDA2 | LRRC4 | OTUB2 | RGS9 | STAT2 | VPS26C |  |
| C15orf61 | DDX39A | GNPNAT1 | LRRC40 | OTUD1 | RGS9BP | STAT3 | VPS28 |  |
| C15orf62 | DDX39B | GNPTAB | LRRC41 | OTUD3 | RHBDD2 | STAT6 | VPS29 |  |
| C16orf46 | DDX3X | GNS | LRRC42 | OTUD5 | RHBDD3 | STAU1 | VPS33A |  |
| C16orf54 | DDX41 | GOLGA1 | LRRC46 | OTUD7B | RHBDF1 | STAU2 | VPS33B |  |
| C16orf72 | DDX42 | GOLGA2 | LRRC4B | OTULIN | RHBDF2 | STC1 | VPS35 |  |
| C16orf74 | DDX46 | GOLGA4 | LRRC51 | OTULINL | RHBDL1 | STC2 | VPS35L |  |
| C16orf86 | DDX47 | GOLGA5 | LRRC56 | OTX1 | RHCE | STEAP3 | VPS36 |  |
| C16orf87 | DDX49 | GOLGA8A | LRRC57 | OTX2 | RHEBL1 | STEAP4 | VPS37A |  |
| C16orf89 | DDX5 | GOLGA8B | LRRC58 | OVCA2 | RHNO1 | STEEP1 | VPS37B |  |
| C16orf90 | DDX50 | GOLGB1 | LRRC59 | OVGP1 | RHOA | STIL | VPS37D |  |
| C16orf91 | DDX51 | GOLIM4 | LRRC61 | OXA1L | RHOB | STIM1 | VPS39 |  |
| C16orf95 | DDX52 | GOLM1 | LRRC73 | OXCT1 | RHOBTB1 | STIM2 | VPS45 |  |
| C17orf100 | DDX54 | GOLM2 | LRRC74B | OXCT2 | RHOBTB2 | STING1 | VPS4A |  |
| C17orf113 | DDX55 | GOLPH3 | LRRC75A | OXLD1 | RHOBTB3 | STIP1 | VPS4B |  |
| C17orf114 | DDX56 | GOLPH3L | LRRC75B | OXNAD1 | RHOC | STK10 | VPS50 |  |
| C17orf49 | DDX58 | GON4L | LRRC8A | OXR1 | RHOF | STK11IP | VPS51 |  |
| C17orf58 | DDX59 | GON7 | LRRC8B | OXSM | RHOG | STK16 | VPS52 |  |
| C17orf67 | DDX6 | GOPC | LRRC8C | OXSR1 | RHOT2 | STK17A | VPS54 |  |
| C17orf75 | DDX60 | GORAB | LRRFIP2 | P2RX1 | RHOU | STK19 | VPS72 |  |
| C17orf80 | DDX60L | GORASP2 | LRRIQ3 | P2RX4 | RHOV | STK24 | VRK1 |  |
| C17orf97 | DEAF1 | GOSR1 | LRRK1 | P2RX5 | RHPN1 | STK25 | VSIG10 |  |
| C17orf98 | DEDD | GOT1 | LRRN2 | P2RX5-TAX1BP3 | RHPN2 | STK26 | VSIG10L |  |
| C17orf99 | DEDD2 | GOT2 | LRRN4CL | P2RX6 | RIBC1 | STK3 | VSIG2 |  |
| C18orf21 | DEFB1 | GPA33 | LRSAM1 | P2RY14 | RIC1 | STK32C | VSIG8 |  |
| C18orf25 | DEFB110 | GPAM | LRTOMT | P2RY2 | RIC8A | STK33 | VSIR |  |
| C18orf32 | DEGS1 | GPANK1 | LRWD1 | P2RY4 | RIC8B | STK35 | VTI1A |  |
| C18orf54 | DENND1A | GPAT3 | LSG1 | P2RY8 | RICTOR | STK36 | VTI1B |  |
| C19orf25 | DENND1B | GPAT4 | LSM1 | P3H1 | RIDA | STK38L | VTN |  |
| C19orf33 | DENND1C | GPATCH2 | LSM10 | P3H4 | RIF1 | STK4 | VWA5A |  |
| C19orf44 | DENND2A | GPATCH2L | LSM12 | P3R3URF | RILP | STK40 | VWA7 |  |
| C19orf47 | DENND2B | GPATCH3 | LSM14A | P3R3URF-PIK3R3 | RILPL1 | STKLD1 | VWA8 |  |
| C19orf53 | DENND2C | GPATCH4 | LSM14B | P4HA1 | RILPL2 | STMN1 | VWDE |  |
| C19orf54 | DENND2D | GPATCH8 | LSM2 | P4HA2 | RIMBP3C | STMN3 | WAC |  |
| C19orf67 | DENND3 | GPBP1 | LSM3 | P4HB | RIMKLA | STMP1 | WAPL |  |
| C19orf71 | DENND4A | GPBP1L1 | LSM4 | P4HTM | RIMKLB | STN1 | WARS1 |  |
| C19orf73 | DENND4B | GPC1 | LSM5 | PA2G4 | RIMOC1 | STOM | WARS2 |  |
| C19orf84 | DENND4C | GPC5 | LSM8 | PAAF1 | RIMS3 | STOML1 | WAS |  |
| C19orf85 | DENND5A | GPCPD1 | LSMEM1 | PABIR2 | RIN1 | STOML2 | WASF1 |  |
| C1GALT1 | DENND5B | GPD1L | LSR | PABIR3 | RINL | STON2 | WASF2 |  |
| C1GALT1C1 | DENND6A | GPD2 | LSS | PABPC1 | RINT1 | STOX1 | WASHC2A | |
| C1orf109 | DENND6B | GPHN | LTA4H | PABPC4 | RIOK1 | STOX2 | WASHC2C | |
| C1orf112 | DENR | GPIHBP1 | LTB | PABPC4L | RIOK3 | STPG1 | WASHC3 |  |
| C1orf116 | DEPDC1B | GPLD1 | LTBP2 | PACC1 | RIOX1 | STPG2 | WASHC4 |  |
| C1orf131 | DEPDC4 | GPN2 | LTBP3 | PACS1 | RIOX2 | STPG4 | WASHC5 |  |
| C1orf159 | DEPDC5 | GPN3 | LTBR | PACS2 | RIPK1 | STRA6 | WASL |  |
| C1orf174 | DEPDC7 | GPR107 | LTN1 | PACSIN2 | RIPK2 | STRADA | WBP2 |  |
| C1orf210 | DEPP1 | GPR108 | LTO1 | PACSIN3 | RIPK3 | STRADB | WBP4 |  |
| C1orf216 | DEPTOR | GPR132 | LTV1 | PAF1 | RIPK4 | STRAP | WDFY1 |  |
| C1orf232 | DERA | GPR135 | LUC7L | PAFAH1B1 | RIPOR1 | STRBP | WDFY2 |  |
| C1orf35 | DERL1 | GPR137 | LUC7L2 | PAFAH1B3 | RIPOR3 | STRC | WDHD1 |  |
| C1orf43 | DERL2 | GPR137C | LUC7L3 | PAFAH2 | RIPPLY1 | STRIP2 | WDPCP |  |
| C1orf50 | DERL3 | GPR152 | LUM | PAGR1 | RIT1 | STRN | WDR1 |  |
| C1orf52 | DERPC | GPR153 | LURAP1L | PAICS | RITA1 | STRN3 | WDR11 |  |
| C1orf53 | DESI1 | GPR155 | LUZP1 | PAIP1 | RLBP1 | STS | WDR12 |  |
| C1orf54 | DESI2 | GPR157 | LXN | PAIP2 | RLF | STT3A | WDR13 |  |
| C1orf56 | DEXI | GPR160 | LY6E | PAK1IP1 | RLIM | STT3B | WDR19 |  |
| C1orf74 | DFFB | GPR161 | LY6G5C | PAK2 | RMC1 | STUB1 | WDR20 |  |
| C1QBP | DGAT1 | GPR171 | LY6G6C | PALB2 | RMDN1 | STX10 | WDR24 |  |
| C1QL1 | DGCR2 | GPR176 | LY6K | PALM3 | RMDN2 | STX11 | WDR25 |  |
| C1QL2 | DGCR6L | GPR179 | LYG2 | PALS1 | RMDN3 | STX12 | WDR26 |  |
| C1QL4 | DGCR8 | GPR180 | LYL1 | PALS2 | RMI1 | STX16 | WDR27 |  |
| C1QTNF12 | DGKD | GPR19 | LYN | PAM | RMND1 | STX16-NPEPL1 | WDR3 |  |
| C1QTNF2 | DGKE | GPR20 | LYPD1 | PAM16 | RMND5A | STX17 | WDR31 |  |
| C1QTNF5 | DGKH | GPR33 | LYPD3 | PAN2 | RNASE11 | STX1A | WDR35 |  |
| C1QTNF6 | DGKQ | GPR35 | LYPD4 | PAN3 | RNASE12 | STX1B | WDR37 |  |
| C1QTNF8 | DGUOK | GPR37 | LYPLA2 | PANK1 | RNASE13 | STX2 | WDR38 |  |
| C1QTNF9B | DHCR24 | GPR37L1 | LYRM1 | PANK2 | RNASE4 | STX4 | WDR4 |  |
| C1R | DHCR7 | GPR39 | LYRM2 | PANK3 | RNASEH1 | STX5 | WDR41 |  |
| C1RL | DHDDS | GPR4 | LYRM4 | PANK4 | RNASEH2A | STX6 | WDR43 |  |
| C2 | DHDH | GPR62 | LYRM9 | PANX1 | RNASEH2B | STX7 | WDR44 |  |
| C20orf141 | DHFR | GPR68 | LYSMD1 | PAPOLA | RNASEH2C | STX8 | WDR45 |  |
| C20orf203 | DHFR2 | GPR78 | LYSMD2 | PAQR4 | RNASEL | STXBP1 | WDR45B |  |
| C20orf27 | DHH | GPR84 | LYSMD3 | PAQR5 | RNASET2 | STXBP2 | WDR46 |  |
| C20orf96 | DHPS | GPR89A | LYSMD4 | PAQR6 | RNF10 | STXBP3 | WDR47 |  |
| C21orf58 | DHRS1 | GPR89B | LYST | PAQR7 | RNF103 | STXBP4 | WDR48 |  |
| C21orf62 | DHRS11 | GPRC5A | LZIC | PAQR8 | RNF103-CHMP3 | STYK1 | WDR53 |  |
| C21orf91 | DHRS12 | GPRC5B | LZTR1 | PAQR9 | RNF11 | STYX | WDR54 |  |
| C22orf31 | DHRS13 | GPRC5C | LZTS2 | PARD3 | RNF113A | STYXL1 | WDR55 |  |
| C22orf39 | DHRS3 | GPS2 | LZTS3 | PARD3B | RNF114 | SUCLA2 | WDR59 |  |
| C2CD2 | DHRS4 | GPSM1 | M1AP | PARD6A | RNF115 | SUCLG1 | WDR6 |  |
| C2CD2L | DHRS7 | GPSM3 | M6PR | PARD6B | RNF121 | SUCLG2 | WDR61 |  |
| C2CD3 | DHRS7C | GPT | MAB21L3 | PARK7 | RNF122 | SUCO | WDR62 |  |
| C2CD4D | DHTKD1 | GPT2 | MAB21L4 | PARL | RNF123 | SUDS3 | WDR7 |  |
| C2CD5 | DHX16 | GPX1 | MACIR | PARN | RNF125 | SUFU | WDR70 |  |
| C2CD6 | DHX30 | GPX4 | MACO1 | PARP1 | RNF126 | SUGP1 | WDR73 |  |
| C2orf15 | DHX33 | GRAMD1C | MACROD1 | PARP10 | RNF13 | SUGP2 | WDR74 |  |
| C2orf42 | DHX34 | GRAMD2A | MACROD2 | PARP12 | RNF130 | SUGT1 | WDR75 |  |
| C2orf49 | DHX35 | GRB10 | MACROH2A1 | PARP14 | RNF135 | SULF2 | WDR76 |  |
| C2orf50 | DHX36 | GRB2 | MAD1L1 | PARP16 | RNF138 | SULT1A1 | WDR77 |  |
| C2orf68 | DHX37 | GRB7 | MAD2L1 | PARP2 | RNF139 | SULT1A2 | WDR81 |  |
| C2orf74 | DHX38 | GREB1 | MAD2L1BP | PARP3 | RNF141 | SULT1E1 | WDR83 |  |
| C2orf76 | DHX40 | GREB1L | MAD2L2 | PARP4 | RNF144B | SULT6B1 | WDR83OS | |
| C2orf81 | DHX57 | GRHL1 | MADCAM1 | PARP6 | RNF145 | SUMF1 | WDR86 |  |
| C3 | DHX58 | GRHPR | MADD | PARP9 | RNF146 | SUMF2 | WDR87 |  |
| C3AR1 | DHX8 | GRID1 | MAEA | PARPBP | RNF149 | SUMO1 | WDR89 |  |
| C3orf18 | DHX9 | GRID2IP | MAF1 | PARS2 | RNF157 | SUMO2 | WDR90 |  |
| C3orf22 | DIABLO | GRIK5 | MAFA | PARVA | RNF166 | SUMO3 | WDR91 |  |
| C3orf33 | DIAPH1 | GRIN2C | MAFB | PASK | RNF167 | SUN1 | WDSUB1 |  |
| C3orf38 | DIAPH2 | GRIPAP1 | MAFG | PATJ | RNF168 | SUN2 | WDTC1 |  |
| C3orf52 | DIAPH3 | GRK4 | MAFK | PATL1 | RNF169 | SUPT16H | WEE1 |  |
| C4BPB | DICER1 | GRK6 | MAGEF1 | PATZ1 | RNF170 | SUPT3H | WFDC12 |  |
| C4orf3 | DIDO1 | GRN | MAGI1 | PAWR | RNF185 | SUPT4H1 | WFDC3 |  |
| C4orf33 | DIMT1 | GRPEL1 | MAGI3 | PAX8 | RNF187 | SUPT7L | WFDC5 |  |
| C4orf36 | DIP2A | GRSF1 | MAGOH | PAXBP1 | RNF19A | SURF1 | WFDC6 |  |
| C4orf45 | DIP2B | GRTP1 | MAGOHB | PBK | RNF19B | SURF2 | WFDC8 |  |
| C5 | DIPK1A | GRWD1 | MAGT1 | PBRM1 | RNF2 | SURF4 | WFIKKN1 |  |
| C5AR1 | DIPK2A | GSC2 | MAIP1 | PBX2 | RNF20 | SURF6 | WFS1 |  |
| C5orf15 | DIRAS1 | GSDMA | MAJIN | PBX3 | RNF207 | SUSD1 | WHAMM |  |
| C5orf22 | DIS3 | GSDMB | MALT1 | PBX4 | RNF208 | SUSD2 | WHRN |  |
| C5orf34 | DIS3L | GSDMD | MAML1 | PBXIP1 | RNF213 | SUSD6 | WIPF2 |  |
| C5orf49 | DIS3L2 | GSDME | MAMSTR | PC | RNF214 | SUV39H1 | WIPI1 |  |
| C6orf120 | DISP1 | GSE1 | MAN1A2 | PCBD1 | RNF215 | SUV39H2 | WIZ |  |
| C6orf132 | DISP2 | GSG1L2 | MAN1B1 | PCBD2 | RNF216 | SV2A | WNK1 |  |
| C6orf136 | DISP3 | GSK3A | MAN1C1 | PCBP2 | RNF217 | SVBP | WNT1 |  |
| C6orf15 | DKC1 | GSK3B | MAN2A1 | PCBP4 | RNF222 | SVIL | WNT10B |  |
| C6orf226 | DKK4 | GSKIP | MAN2A2 | PCCA | RNF223 | SVIP | WNT11 |  |
| C6orf47 | DKKL1 | GSR | MAN2B1 | PCCB | RNF224 | SWAP70 | WNT2B |  |
| C6orf52 | DLAT | GSS | MAN2C1 | PCDH1 | RNF225 | SWI5 | WNT4 |  |
| C6orf62 | DLG1 | GSTA1 | MANBA | PCDH12 | RNF227 | SWSAP1 | WNT5A |  |
| C7orf25 | DLG4 | GSTA3 | MANBAL | PCED1A | RNF24 | SWT1 | WNT7B |  |
| C7orf50 | DLG5 | GSTA5 | MANEA | PCED1B | RNF25 | SYAP1 | WNT9A |  |
| C7orf61 | DLGAP5 | GSTCD | MANSC1 | PCF11 | RNF31 | SYBU | WRAP53 |  |
| C8G | DLK2 | GSTK1 | MANSC4 | PCGF1 | RNF32 | SYCE2 | WRAP73 |  |
| C8orf44-SGK3 | DLL1 | GSTM3 | MAOA | PCGF2 | RNF34 | SYCE3 | WRN |  |
| C8orf76 | DLL4 | GSTO2 | MAOB | PCGF3 | RNF38 | SYCN | WRNIP1 |  |
| C8orf82 | DLST | GSTP1 | MAP1A | PCGF5 | RNF39 | SYCP2 | WSB1 |  |
| C9orf131 | DLX1 | GSTT2B | MAP1LC3C | PCGF6 | RNF4 | SYDE2 | WWC2 |  |
| C9orf24 | DLX2 | GSTT4 | MAP1S | PCID2 | RNF40 | SYF2 | WWC3 |  |
| C9orf40 | DLX3 | GSTZ1 | MAP2K1 | PCIF1 | RNF41 | SYMPK | WWOX |  |
| C9orf43 | DLX4 | GTF2A1 | MAP2K2 | PCK1 | RNF43 | SYNC | WWP1 |  |
| C9orf50 | DMAC2L | GTF2A2 | MAP2K5 | PCK2 | RNF44 | SYNCRIP | WWTR1 |  |
| C9orf64 | DMD | GTF2B | MAP3K1 | PCLAF | RNF7 | SYNDIG1L | XAB2 |  |
| C9orf72 | DMKN | GTF2E2 | MAP3K10 | PCM1 | RNF8 | SYNE2 | XBP1 |  |
| C9orf78 | DMPK | GTF2F1 | MAP3K11 | PCMT1 | RNFT1 | SYNE3 | XCL2 |  |
| C9orf85 | DMRTA1 | GTF2H1 | MAP3K12 | PCMTD1 | RNFT2 | SYNE4 | XCR1 |  |
| CA11 | DMRTA2 | GTF2H2C | MAP3K14 | PCMTD2 | RNH1 | SYNGR4 | XDH |  |
| CA12 | DMTF1 | GTF2H3 | MAP3K15 | PCNA | RNLS | SYNJ1 | XIAP |  |
| CA2 | DMWD | GTF2H4 | MAP3K2 | PCNP | RNMT | SYNJ2 | XK |  |
| CA5A | DMXL1 | GTF2IRD2B | MAP3K20 | PCNT | RNPC3 | SYNJ2BP | XKR5 |  |
| CA5B | DMXL2 | GTF3C1 | MAP3K21 | PCNX1 | RNPEP | SYNJ2BP-COX16 | XKR7 |  |
| CA9 | DNA2 | GTF3C2 | MAP3K3 | PCNX2 | RNPS1 | SYNPO2L | XKR8 |  |
| CAAP1 | DNAAF1 | GTF3C4 | MAP3K5 | PCNX3 | RO60 | SYNRG | XKR9 |  |
| CAB39 | DNAAF10 | GTPBP2 | MAP3K6 | PCOLCE2 | ROCK1 | SYP | XKRX |  |
| CABIN1 | DNAAF2 | GTPBP3 | MAP3K8 | PCP2 | ROGDI | SYPL1 | XNDC1N |  |
| CABLES1 | DNAAF3 | GTPBP6 | MAP3K9 | PCSK1 | ROM1 | SYS1-DBNDD2 | XNDC1N-ZNF705EP-ALG1L9P | |
| CABLES2 | DNAAF8 | GTPBP8 | MAP4K1 | PCSK4 | ROMO1 | SYT11 | XPA |  |
| CABP2 | DNAH12 | GTSE1 | MAP4K2 | PCSK6 | ROPN1L | SYT17 | XPC |  |
| CABP5 | DNAH17 | GTSF1L | MAP4K4 | PCSK7 | RORC | SYT2 | XPNPEP1 |  |
| CACFD1 | DNAH3 | GUCA1B | MAP4K5 | PCSK9 | ROS1 | SYT5 | XPNPEP3 |  |
| CACNA1F | DNAH5 | GUCA2A | MAP6D1 | PCTP | RP1L1 | SYT7 | XPO4 |  |
| CACNA1S | DNAI4 | GUF1 | MAP7D1 | PCYOX1 | RP2 | SYT8 | XPO5 |  |
| CACNA2D1 | DNAJA2 | GUSB | MAP7D2 | PCYT1A | RPA1 | SYTL4 | XPO6 |  |
| CACNB1 | DNAJA3 | GVQW3 | MAP7D3 | PCYT2 | RPA2 | SYVN1 | XPOT |  |
| CACNB3 | DNAJA4 | GXYLT1 | MAPK1 | PDAP1 | RPAIN | SZRD1 | XPR1 |  |
| CACTIN | DNAJB1 | GYG1 | MAPK10 | PDCD10 | RPAP1 | TAB2 | XRCC1 |  |
| CACUL1 | DNAJB11 | GYS1 | MAPK11 | PDCD2 | RPAP2 | TAB3 | XRCC2 |  |
| CACYBP | DNAJB12 | GZF1 | MAPK12 | PDCD2L | RPAP3 | TACC3 | XRCC3 |  |
| CAD | DNAJB14 | H1-1 | MAPK14 | PDCD4 | RPE | TACO1 | XRCC4 |  |
| CADM4 | DNAJB2 | H1-10 | MAPK1IP1L | PDCD5 | RPE65 | TACR2 | XRCC6 |  |
| CADPS2 | DNAJB4 | H1-2 | MAPK3 | PDCD6 | RPF1 | TACSTD2 | XRN1 |  |
| CAGE1 | DNAJB5 | H1-3 | MAPK6 | PDCD6-AHRR | RPF2 | TADA1 | XRN2 |  |
| CALCA | DNAJB7 | H1-4 | MAPK8 | PDCD6IP | RPGR | TADA2A | XRRA1 |  |
| CALCOCO2 | DNAJB8 | H1-5 | MAPK8IP3 | PDCD7 | RPGRIP1L | TADA2B | XXYLT1 |  |
| CALCR | DNAJB9 | H1-6 | MAPK9 | PDCL | RPIA | TADA3 | XYLB |  |
| CALHM1 | DNAJC1 | H2AC14 | MAPKAP1 | PDCL3 | RPL10 | TAF1 | XYLT2 |  |
| CALHM2 | DNAJC10 | H2AC15 | MAPKAPK3 | PDE11A | RPL10A | TAF10 | YAE1 |  |
| CALHM3 | DNAJC11 | H2AC17 | MAPKAPK5 | PDE12 | RPL11 | TAF11 | YAF2 |  |
| CALM1 | DNAJC13 | H2AC20 | MAPKBP1 | PDE2A | RPL12 | TAF11L10 | YAP1 |  |
| CALM2 | DNAJC16 | H2AC21 | MAPRE1 | PDE3A | RPL13 | TAF11L3 | YARS1 |  |
| CALM3 | DNAJC17 | H2AC4 | MAPT | PDE3B | RPL15 | TAF11L8 | YARS2 |  |
| CALML4 | DNAJC18 | H2AC7 | MARCHF2 | PDE4A | RPL17 | TAF11L9 | YBEY |  |
| CALR | DNAJC19 | H2AC8 | MARCHF3 | PDE4C | RPL17-C18orf32 | TAF12 | YBX1 |  |
| CALR3 | DNAJC21 | H2AJ | MARCHF5 | PDE5A | RPL18 | TAF13 | YBX2 |  |
| CAMK1 | DNAJC22 | H2AW | MARCHF6 | PDE6A | RPL22 | TAF15 | YBX3 |  |
| CAMK2A | DNAJC25 | H2AX | MARCHF7 | PDE6D | RPL22L1 | TAF1B | YDJC |  |
| CAMK2G | DNAJC25-GNG10 | H2AZ1 | MARCKSL1 | PDE6G | RPL23 | TAF1C | YEATS2 |  |
| CAMK2N1 | DNAJC28 | H2AZ2 | MARF1 | PDE7A | RPL24 | TAF1D | YIF1A |  |
| CAMKK1 | DNAJC3 | H2BC10 | MARK1 | PDE7B | RPL26 | TAF2 | YIF1B |  |
| CAMKK2 | DNAJC30 | H2BC11 | MARK3 | PDE8A | RPL27 | TAF3 | YIPF1 |  |
| CAMKMT | DNAJC5 | H2BC12 | MARK4 | PDE9A | RPL29 | TAF4 | YIPF2 |  |
| CAMKV | DNAJC7 | H2BC13 | MARS1 | PDF | RPL3 | TAF4B | YIPF3 |  |
| CAMLG | DNAJC8 | H2BC17 | MARS2 | PDGFA | RPL30 | TAF5 | YIPF5 |  |
| CAMP | DNAJC9 | H2BC21 | MARVELD1 | PDGFB | RPL35 | TAF5L | YIPF6 |  |
| CAMSAP1 | DNAL1 | H2BC3 | MARVELD2 | PDGFRB | RPL36A | TAF6 | YIPF7 |  |
| CAMSAP2 | DNAL4 | H2BC6 | MASP1 | PDHA1 | RPL36A-HNRNPH2 | TAF6L | YLPM1 |  |
| CAMTA1 | DNASE1L1 | H2BC8 | MASP2 | PDHB | RPL36AL | TAF7 | YOD1 |  |
| CAMTA2 | DNASE1L3 | H2BK1 | MAST1 | PDHX | RPL37 | TAF9 | YPEL2 |  |
| CAND1 | DNASE2 | H3-3A | MAST2 | PDIA4 | RPL39 | TAF9B | YPEL3 |  |
| CANT1 | DND1 | H3-3B | MAT2A | PDIA5 | RPL39L | TAFA2 | YPEL4 |  |
| CANX | DNER | H3-4 | MATN3 | PDIA6 | RPL3L | TAFA3 | YTHDC1 |  |
| CAP1 | DNLZ | H3C1 | MATN4 | PDIK1L | RPL4 | TAFAZZIN | YTHDF1 |  |
| CAP2 | DNM1 | H3C10 | MATR3 | PDK1 | RPL5 | TAGLN2 | YTHDF2 |  |
| CAPG | DNM1L | H3C11 | MAU2 | PDK2 | RPL6 | TALDO1 | YTHDF3 |  |
| CAPN1 | DNM2 | H3C12 | MAVS | PDK3 | RPL7 | TAMM41 | YWHAB |  |
| CAPN10 | DNMT1 | H3C2 | MAX | PDK4 | RPL7A | TANC1 | YWHAE |  |
| CAPN15 | DNMT3A | H3C3 | MAZ | PDLIM1 | RPL8 | TANC2 | YWHAG |  |
| CAPN2 | DNMT3B | H3C4 | MB | PDLIM5 | RPL9 | TAOK1 | YWHAH |  |
| CAPN5 | DNMT3L | H3C7 | MBD1 | PDLIM7 | RPLP0 | TAOK3 | YWHAQ |  |
| CAPN8 | DNPH1 | H3C8 | MBD2 | PDP1 | RPN1 | TAP1 | YWHAZ |  |
| CAPNS1 | DNTTIP1 | H3Y2 | MBD3 | PDP2 | RPN2 | TAP2 | YY1 |  |
| CAPRIN1 | DNTTIP2 | H4-16 | MBD3L1 | PDPR | RPP14 | TAPBP | YY1AP1 |  |
| CAPRIN2 | DOCK5 | H4C1 | MBD3L3 | PDRG1 | RPP21 | TARBP1 | ZAR1L |  |
| CAPZA1 | DOCK6 | H4C12 | MBD3L4 | PDS5A | RPP25 | TARBP2 | ZBED2 |  |
| CAPZA2 | DOCK7 | H4C13 | MBD4 | PDS5B | RPP25L | TARDBP | ZBED3 |  |
| CAPZB | DOCK9 | H4C15 | MBD5 | PDSS1 | RPP38 | TARS1 | ZBED4 |  |
| CARD10 | DOHH | H4C2 | MBLAC2 | PDSS2 | RPP40 | TARS3 | ZBED5 |  |
| CARD16 | DOK1 | H4C3 | MBNL1 | PDXDC1 | RPRD1A | TAS1R1 | ZBED8 |  |
| CARD19 | DOK2 | H4C4 | MBOAT1 | PDZD11 | RPRD2 | TAS1R3 | ZBTB1 |  |
| CARD8 | DOK3 | H4C7 | MBOAT4 | PDZD2 | RPRM | TAS2R50 | ZBTB11 |  |
| CARD9 | DOK4 | H4C8 | MBOAT7 | PDZD7 | RPS10 | TASOR | ZBTB12 |  |
| CARF | DOLK | H4C9 | MBTD1 | PDZD8 | RPS10-NUDT3 | TASOR2 | ZBTB14 |  |
| CARHSP1 | DONSON | H6PD | MBTPS1 | PDZD9 | RPS11 | TASP1 | ZBTB17 |  |
| CARM1 | DOP1A | HAAO | MCAM | PDZK1 | RPS14 | TATDN1 | ZBTB18 |  |
| CARMIL1 | DOP1B | HABP4 | MCAT | PEAK3 | RPS15 | TATDN2 | ZBTB2 |  |
| CARNMT1 | DOT1L | HACD1 | MCCC1 | PEBP1 | RPS15A | TATDN3 | ZBTB20 |  |
| CARS1 | DPAGT1 | HACD2 | MCCC2 | PECR | RPS16 | TAX1BP3 | ZBTB21 |  |
| CARS2 | DPEP2 | HACD3 | MCEE | PEDS1 | RPS17 | TBC1D10A | ZBTB22 |  |
| CASC3 | DPEP2NB | HACE1 | MCF2L2 | PEDS1-UBE2V1 | RPS18 | TBC1D10B | ZBTB24 |  |
| CASD1 | DPEP3 | HACL1 | MCFD2 | PEF1 | RPS19 | TBC1D12 | ZBTB25 |  |
| CASKIN1 | DPF1 | HADH | MCIDAS | PEG10 | RPS19BP1 | TBC1D13 | ZBTB26 |  |
| CASKIN2 | DPF2 | HADHA | MCL1 | PELI1 | RPS2 | TBC1D14 | ZBTB3 |  |
| CASP1 | DPH1 | HADHB | MCM2 | PELO | RPS20 | TBC1D15 | ZBTB33 |  |
| CASP10 | DPH3 | HAGH | MCM3 | PELP1 | RPS21 | TBC1D16 | ZBTB34 |  |

Table S13. Candidate SNPs and their SNaPshot primer sequences

| **Variant** | **Position** | **Allele** | **MAF** | **Upstream Primer** | **Downstream Primer** | **PCR Product Length** | **Extension Primer** | **Extension Direction** |
| --- | --- | --- | --- | --- | --- | --- | --- | --- |
| rs1363630 | 148784795 | GCA[G/C]GAA | 0.16 | AGACCTCACAGCTAGGAAGTG | GCTGAATCAGACACTGTCCCTG | 175bp | TTTTTTTTTTTTCAGCTAGGAAGTGGCCAAGGCA | Forward |
| rs726953 | 148784809 | GGC[C/T]GGT | 0.06 | AGACCTCACAGCTAGGAAGTG | GCTGAATCAGACACTGTCCCTG | 175bp | AATTCTGTTGGTGTTGAAAGGC | Forward |
| rs11168098 | 148785100 | GGA[A/C]CTG | 0.33 | CCCACTCAGGATGTCACAAGGC | GGTCCTGCCTTCCACTGCG | 221bp | TTTTTTTTTTTTTTTTATCCAGGCTGGGGGTCTGAGGA | Forward |
| rs17796636 | 148785449 | AAG[C/T]GGG | 0.3 | CACACTCTCATGAAAGAGCTA | TTGCACTTGCATTGCTCGCTG | 232bp | CACATGCGTGGCGAGTCCGAAG | Forward |
| rs74592187 | 148785511 | AGG[A/G]GAC | 0.3 | CACACTCTCATGAAAGAGCTA | TTGCACTTGCATTGCTCGCTG | 232bp | TTTCAGGGAATGGCTCTTCCAGAGG | Forward |
| rs12517403 | 148785851 | GTC[T/C]GGG | 0.31 | TGGGTCAGGATCTTCCCTGTC | GACTGCAGTGGGAGTTCTGAGA | 189bp | TTTTTTTTTTATGGGAGTAGCTTCCCACGGTC | Forward |
| rs76481424 | 148785898 | ACC[C/T]AGA | 0.31 | TGGGTCAGGATCTTCCCTGTC | GACTGCAGTGGGAGTTCTGAGA | 189bp | TTTTTTTTTTTTTCCAGTCCTGGCTCCTGGAATCT | Reverse |
| rs12658655 | 148787210 | AGA[C/T]GGG | 0.31 | TGTAAGCTCAGGCTCTGGCAG | TGTCCAATGGCCTTGCTATAC | 216bp | TTTTTTTTTTTTTTTTTTAAGGGATGCTGGTGAGAGGAGA | Forward |
| rs368510 | 148787469 | TGT[G/A]GGA | 0.13 | AGGAGGTCCCTGAGCCGAGTC | CATCCCTTACCTACTTCCATG | 253bp | TTTGTCCAAGCAGATGAAGTCCTGT | Forward |
| rs12652986 | 148787662 | AGA[T/C]GGC | 0.32 | CTGATCTCTGGATTCAACCAC | AGAGGAGCCCGACTCCATGCT | 211bp | AGTAGGTAAGGGATGGAAAAGA | Forward |
| rs13177623 | 148787707 | TTA[G/A]AGG | 0.24 | TAAGGGATGGAAAAGATGGCAG | CAAGAAGCGGGAAGGTGGAG | 253bp | ATCACAAGCCTCCTGCATTCCT | Reverse |
| rs17723799 | 148807077 | CTA[C/T]ACC | 0.34 | GTCCTCAGGAAGTGGAGATCTAG | CCAGCCCCTCCTTTACTTCT | 321bp | TTTTTTACCTGGGCTTATGTGGCATCTA | Forward |
| rs353293 | 148807226 | CTC[G/A]CCC | 0.11 | GTCCTCAGGAAGTGGAGATCTAG | CCAGCCCCTCCTTTACTTCT | 321bp | TTTTTTTTTGCACCCCCTGCTTCATGTTCTC | Forward |
| rs353292 | 148807808 | TTG[C/T]CCT | 0.11 | CTCCAACCTATCTTTCTTCTGC | GGAGAATGGAAGGACATCTATG | 236bp | TTTATCCCAAAAGGGCTCCCCATTG | Forward |
| rs4705342 | 148807971 | CCA[T/C]GAA | 0.33 | GGAGGAGTGGCAGAAGAAAGAT | GCTCCTTATCCTTAGACAACCC | 245bp | TCCAGCCTAACAGCATCATTC | Reverse |
| rs4705343 | 148808081 | ATA[T/C]AGA | 0.34 | GGAGGAGTGGCAGAAGAAAGAT | GCTCCTTATCCTTAGACAACCC | 245bp | TTTTTTTTCTATCTCTATAGATCTGTCT | Reverse |
| rs41291957 | 148808390 | CCG[G/A]CCA | 0.33 | TTGTACTCACTAAATGTCCTCCT | CAGGAGAAGGGGTGTTAGAG | 214bp | TGAGGAATTACAACAGCCTCCCG | Forward |
| rs59810862 | 148808778 | TCC[-/A]ATC | 0 | ACAGGAAGGACAGAGTGTTTC | TACTGATTAGCTGTGTCTGCC | 231bp | TTTTTTTTTCTGTGTACCCCAGTGTGTCCA | Forward |
| rs55945735 | 148809158 | TCC[A/G]CGC | 0.01 | GTAGCTGAGATTACAGGTGTG | AGGGCTCATTGTTGAGGATTC | 231bp | GGGATTACAGGCATGAGCCTCC | Forward |
| rs73798217 | 148809918 | CCA[G/A]GCT | 0 | ATACTACAGGCTTCCTTCCTC | TCATGTGTATTCCCCAGTCTC | 223bp | TTTTTTTTTTAACCAACCATTCAGTTCCTAGC | Reverse |

Table S14. Association of SNPs in CARMN with risk of CC in the first stage

| SNPs | Genotype | Cases (n = 571) | |  | Controls (n = 657) | | *P* ^a^ | Adjusted OR | |
| --- | --- | --- | --- | --- | --- | --- | --- | --- | --- |
|  |  | n | % |  | n | % |  | (95% CI)^b^ | |
| rs1363630 | GG | 411 | 73.4 |  | 461 | 70.8 | 0.504 | 1.00 (Ref) | |
|  | GC | 137 | 24.5 |  | 171 | 26.3 |  | 0.96 (0.72-1.29) | |
|  | CC | 12 | 2.1 |  | 19 | 2.9 |  | 0.76 (0.33-1.74) | |
|  |  |  |  |  |  |  |  |  | |
| rs726953 | CC | 457 | 81.6 |  | 569 | 87.4 | **0.015** | 1.00 (Ref) | |
|  | CT | 100 | 17.9 |  | 81 | 12.4 |  | 1.68 (1.18-2.40) | |
|  | TT | 3 | 0.5 |  | 1 | 0.2 |  | 3.28 (0.31-34.72) | |
|  |  |  |  |  |  |  |  |  | |
|  | CC | 457 | 81.6 |  | 569 | 87.4 | 0.005 | 1.00 (Ref) | |
|  | CT/TT | 103 | 18.4 |  | 82 | 12.6 |  | 1.71 (1.20-2.43) | |
|  |  |  |  |  |  |  |  |  | |
| rs11168098 | AA | 221 | 39.4 |  | 293 | 45 | 0.149 | 1.00 (Ref) | |
|  | AC | 273 | 48.8 |  | 290 | 44.6 |  | 1.24 (0.95-1.61) | |
|  | CC | 66 | 11.8 |  | 68 | 10.4 |  | 1.38 (0.91-2.10) | |
|  |  |  |  |  |  |  |  |  | |
| rs12517403 | TT | 193 | 35 |  | 289 | 45.1 | **0.0003** | 1.00 (Ref) | |
|  | TC | 279 | 50.5 |  | 293 | 45.7 |  | 1.41 (1.09-1.83) | |
|  | CC | 80 | 14.5 |  | 59 | 9.2 |  | 1.75 (1.17-2.62) | |
|  |  |  |  |  |  |  |  |  | |
|  | TT | 193 | 35 |  | 289 | 45.1 | **0.0004** | 1.00 (Ref) | |
|  | TC/CC | 359 | 65 |  | 352 | 54.9 |  | 1.48 (1.15-1.89) | |
|  |  |  |  |  |  |  |  |  | |
|  |  |  |  |  |  |  |  |  | |
| rs12652986 | TT | 241 | 42.3 |  | 303 | 46.3 | 0.362 | 1.00 (Ref) | |
|  | TC | 269 | 47.2 |  | 288 | 44.1 |  | 1.17 (0.90-1.52) | |
|  | CC | 60 | 10.5 |  | 63 | 9.6 |  | 1.20 (0.79-1.85) | |
|  |  |  |  |  |  |  |  |  | |
| rs13177623 | GG | 279 | 49.8 |  | 376 | 57.8 | 0.022 | 1.00 (Ref) | |
|  | GA | 238 | 42.5 |  | 234 | 35.9 |  | 1.37 (1.06-1.79) | |
|  | AA | 43 | 7.7 |  | 41 | 6.3 |  | 1.44 (0.87-2.37) | |
|  |  |  |  |  |  |  |  |  | |
|  | GG | 279 | 49.8 |  | 376 | 57.8 | 0.006 | 1.00 (Ref) | |
|  | GA/AA | 281 | 50.2 |  | 275 | 42.2 |  | 1.38 (1.08-1.78) | |
|  |  |  |  |  |  |  |  |  | |
| rs17723799 | CC | 241 | 43 |  | 277 | 42.5 | 0.156 | 1.00 (Ref) | |
|  | CT | 246 | 43.9 |  | 312 | 47.7 |  | 0.94 (0.72-1.22) | |
|  | TT | 73 | 13.1 |  | 64 | 9.8 |  | 1.36 (0.90-2.06) | |
|  |  |  |  |  |  |  |  |  | |
| rs353293 | GG | 433 | 77.3 |  | 520 | 79.8 | 0.316 | 1.00 (Ref) | |
|  | GA | 117 | 20.9 |  | 126 | 19.3 |  | 1.06 (0.77-1.45) | |
|  | AA | 10 | 1.8 |  | 6 | 0.9 |  | 2.07 (0.61-7.07) | |
|  |  |  |  |  |  |  |  |  | |
| rs353292 | CC | 433 | 77.3 |  | 520 | 79.8 | 0.316 | 1.00 (Ref) | |
|  | CT | 117 | 20.9 |  | 126 | 19.3 |  | 1.06 (0.77-1.45) | |
|  | TT | 10 | 1.8 |  | 6 | 0.9 |  | 2.07 (0.61-7.07) | |
|  |  |  |  |  |  |  |  |  | |
| rs4705342 | TT | 254 | 45.4 |  | 276 | 42.3 | 0.085 | 1.00 (Ref) | |
|  | TC | 242 | 43.2 |  | 319 | 48.9 |  | 0.86 (0.66-1.11) | |
|  | CC | 64 | 11.4 |  | 57 | 8.8 |  | 1.32 (0.86-2.04) | |
|  |  |  |  |  |  |  |  |  | |
| rs4705343 | TT | 253 | 45.2 |  | 273 | 41.9 | 0.15 | 1.00 (Ref) | |
|  | TC | 246 | 43.9 |  | 321 | 49.2 |  | 0.86 (0.66-1.12) | |
|  | CC | 61 | 10.9 |  | 58 | 8.9 |  | 1.19 (0.77-1.84) | |
|  |  |  |  |  |  |  |  |  | |
| rs41291957 | GG | 262 | 46.8 |  | 287 | 44 | 0.163 | 1.00 (Ref) | |
|  | GA | 235 | 42 |  | 306 | 46.9 |  | 0.89 (0.69-1.16) | |
|  | AA | 63 | 11.2 |  | 59 | 9.1 |  | 1.21 (0.79-1.86) | |
| ^a^ χ^2^ test for either genotype distributions or allele frequencies between the cases and controls. | | | | | | | | |  |
| ^b^ Adjusted for age, parity and menopausal status in logistic regression model. | | | | | | | | |  |
| Bold numbers indicate statistically significant differences between groups, with a p-value < 0.05. | | | | | | | | |  |

Table S15. Prediction of TFs binding to the promoter region of CARMN by Alibaba 2.0

| **TFs** | **Predicted binding numbers** |
| --- | --- |
| SP1 | 42 |
| NF-1 | 12 |
| C/EBP-α | 8 |
| OCT-1 | 8 |
| NF-kappaB | 7 |
| GR | 6 |
| AP-2α | 3 |
| AP-1 | 2 |
| c-Fos | 2 |
| Egr-1 | 2 |
| ER | 2 |
| GATA-1 | 2 |
| MIG1 | 2 |
| Pit-1 | 2 |
| RAP1 | 2 |
| RAR-α | 2 |
| RXR-β | 2 |
| USF | 2 |
| ACE2 | 1 |
| Adf-1 | 1 |
| ADR1 | 1 |
| C/EBP-β | 1 |
| c-Ets-1 | 1 |
| c-Jun | 1 |
| CP1 | 1 |
| E1 | 1 |
| EFI | 1 |
| HSTF | 1 |
| IRF-1 | 1 |
| MEB-1 | 1 |
| MyoD | 1 |
| T3R-α | 1 |
| T3R-β | 1 |
| TBP | 1 |
| TEC1 | 1 |
| WT1 | 1 |

Table S16. CARMN sense and antisense strand-bound proteins

| **Position** | **Acc** | **unused** | **Coverage (%)** | **Mass** | **Symbol** |
| --- | --- | --- | --- | --- | --- |
| sense | sp\|P63261\|ACTG | 63.81 | 89.07 | 41792.5 | ACTG1 ACTG |
| sense | sp\|P08238\|HS90B | 36.83 | 37.43 | 83263.5 | HSP90AB1 HSP90B HSPC2 HSPC3 HSPCB |
| sense | sp\|P06733\|ENOA | 35.44 | 58.76 | 47168.6 | ENO1 ENO1L1 MBPB1 MPB1 |
| sense | sp\|P04406\|G3P | 34.19 | 83.28 | 36053 | GAPDH GAPD CDABP0047 OK/SW-cl.12 |
| sense | sp\|P10809\|CH60 | 28.09 | 44.85 | 61054.2 | HSPD1 HSP60 |
| sense | sp\|P07437\|TBB5 | 28.03 | 52.93 | 49670.5 | TUBB TUBB5 OK/SW-cl.56 |
| sense | sp\|P68104\|EF1A1 | 23.34 | 45.24 | 50140.6 | EEF1A1 EEF1A EF1A LENG7 |
| sense | sp\|P14618\|KPYM | 22 | 34.09 | 57936.4 | PKM OIP3 PK2 PK3 PKM2 |
| sense | sp\|P11021\|GRP78 | 22 | 30.73 | 72332.4 | HSPA5 GRP78 |
| sense | sp\|P68363\|TBA1B | 16 | 39.69 | 50151.2 | TUBA1B |
| sense | sp\|P62937\|PPIA | 16 | 67.27 | 18012.4 | PPIA CYPA |
| sense | sp\|P00338\|LDHA | 15.94 | 35.24 | 36688.5 | LDHA PIG19 |
| sense | sp\|P78371\|TCPB | 15.3 | 25.42 | 57487.6 | CCT2 99D8.1 CCTB |
| sense | sp\|P13639\|EF2 | 14.75 | 14.92 | 95337.4 | EEF2 EF2 |
| sense | sp\|Q9BTM1\|H2AJ;sp\|Q99878\|H2A1J;sp\|Q96KK5\|H2A1H;sp\|P20671\|H2A1D;sp\|P0C0S8\|H2A1 | 14.04 | 58.14 | 13936.2 | H2AJ H2AFJ H2AC12 H2AC7 H2AC11; H2AC13; H2AC15; H2AC16; H2AC17 |
| sense | sp\|P00558\|PGK1 | 14.03 | 33.33 | 44614.4 | PGK1 PGKA MIG10 OK/SW-cl.110 |
| sense | sp\|P07900\|HS90A | 14 | 34.02 | 84659 | HSP90AA1 HSP90A HSPC1 HSPCA |
| sense | sp\|P38646\|GRP75 | 14 | 25.48 | 73680 | HSPA9 GRP75 HSPA9B mt-HSP70 |
| sense | sp\|P53396\|ACLY | 12.11 | 12.08 | 120838.3 | ACLY |
| sense | sp\|P11142\|HSP7C | 12 | 19.04 | 70897.6 | HSPA8 HSC70 HSP73 HSPA10 |
| sense | sp\|P62805\|H4 | 10.17 | 42.72 | 11367.3 | H4C1 H4/A H4FA HIST1H4A; H4C2 H4/I H4FI HIST1H4B; H4C3 H4/G H4FG HIST1H4C; H4C4 H4/B H4FB HIST1H4D; H4C5 H4/J H4FJ HIST1H4E; H4C6 H4/C H4FC HIST1H4F; H4C8 H4/H H4FH HIST1H4H; H4C9 H4/M H4FM HIST1H4I; H4C11 H4/E H4FE HIST1H4J; H4C12 H4/D H4FD HIST1H4K; H4C13 H4/K H4FK HIST1H4L; H4C14 H4/N H4F2 H4FN HIST2H4 HIST2H4A; H4C15 H4/O H4FO HIST2H4B; H4C16 H4-16 HIST4H4 |
| sense | sp\|P07814\|SYEP | 10 | 6.08 | 170589.7 | EPRS1 EPRS GLNS PARS QARS QPRS PIG32 |
| sense | sp\|P07355\|ANXA2 | 10 | 17.70 | 38603.6 | ANXA2 ANX2 ANX2L4 CAL1H LPC2D |
| sense | sp\|P14625\|ENPL | 9.91 | 18.31 | 92468.1 | HSP90B1 GRP94 HSPC4 TRA1 |
| sense | sp\|P11498\|PYC | 9.69 | 4.41 | 129632.6 | PC |
| sense | sp\|Q9UHB6\|LIMA1 | 9.41 | 7.38 | 85224.9 | LIMA1 EPLIN SREBP3 PP624 |
| sense | sp\|P07195\|LDHB | 8.08 | 25.15 | 36638.2 | LDHB |
| sense | sp\|P06576\|ATPB | 8 | 19.09 | 56559.4 | ATP5F1B ATP5B ATPMB ATPSB |
| sense | sp\|P68431\|H31 | 8 | 29.41 | 15403.9 | H3C1 H3FA HIST1H3A; H3C2 H3FL HIST1H3B; H3C3 H3FC HIST1H3C; H3C4 H3FB HIST1H3D; H3C6 H3FD HIST1H3E; H3C7 H3FI HIST1H3F; H3C8 H3FH HIST1H3G; H3C10 H3FK HIST1H3H; H3C11 H3FF HIST1H3I; H3C12 H3FJ HIST1H3J |
| sense | sp\|P60174\|TPIS | 8 | 26.92 | 30790.8 | TPI1 TPI |
| sense | sp\|P31943\|HNRH1 | 6.95 | 12.47 | 49229.3 | HNRNPH1 HNRPH HNRPH1 |
| sense | sp\|P19338\|NUCL | 6.87 | 8.45 | 76613.9 | NCL |
| sense | sp\|Q16643\|DREB | 6.82 | 6.63 | 71428.6 | DBN1 D0S117E |
| sense | sp\|P06748\|NPM | 6.41 | 19.05 | 32574.8 | NPM1 NPM |
| sense | sp\|P04075\|ALDOA | 6.1 | 31.32 | 39419.7 | ALDOA ALDA |
| sense | sp\|Q32P51\|RA1L2;sp\|P09651\|ROA1 | 6.03 | 18.44 | 34225.3 | HNRNPA1L2 HNRNPA1L |
| sense | sp\|P63104\|1433Z | 6.02 | 16.33 | 27744.8 | YWHAZ |
| sense | sp\|Q15365\|PCBP1 | 6.01 | 17.13 | 37497.5 | PCBP1 |
| sense | sp\|P61978\|HNRPK | 6 | 9.29 | 50975.8 | HNRNPK HNRPK |
| sense | sp\|P40227\|TCPZ | 6 | 7.53 | 58023.6 | CCT6A CCT6 CCTZ |
| sense | sp\|Q06830\|PRDX1 | 6 | 20.60 | 22110.2 | PRDX1 PAGA PAGB TDPX2 |
| sense | sp\|Q00610\|CLH1 | 6 | 1.91 | 191613 | CLTC CLH17 CLTCL2 KIAA0034 |
| sense | sp\|P60842\|IF4A1 | 6 | 10.10 | 46153.5 | EIF4A1 DDX2A EIF4A |
| sense | sp\|P11586\|C1TC | 6 | 10.27 | 101558.4 | MTHFD1 MTHFC MTHFD |
| sense | sp\|P08670\|VIME | 6 | 7.72 | 53651.2 | VIM |
| sense | sp\|P07737\|PROF1 | 6 | 26.43 | 15054.1 | PFN1 |
| sense | sp\|P05783\|K1C18 | 5.28 | 12.79 | 48057.4 | KRT18 CYK18 PIG46 |
| sense | sp\|Q9UQ80\|PA2G4 | 5.11 | 18.02 | 43786.6 | PA2G4 EBP1 |
| sense | sp\|P17987\|TCPA | 5.04 | 8.27 | 60342.9 | TCP1 CCT1 CCTA |
| sense | sp\|P15880\|RS2 | 4.75 | 10.24 | 31324.2 | RPS2 RPS4 |
| sense | sp\|P35232\|PHB | 4.66 | 16.18 | 29803.8 | PHB1 PHB |
| sense | sp\|P49327\|FAS | 4.21 | 2.15 | 273424.1 | FASN FAS |
| sense | sp\|P26641\|EF1G | 4.16 | 16.93 | 50118.4 | EEF1G EF1G PRO1608 |
| sense | sp\|Q99880\|H2B1L;sp\|Q99877\|H2B1N;sp\|Q93079\|H2B1H;sp\|P62807\|H2B1C;sp\|P58876\|H2B1D;sp\|P57053\|H2BFS;sp\|O60814\|H2B1K | 4.05 | 19.05 | 13890 | H2BC13 H2BFC HIST1H2BL |
| sense | sp\|P29401\|TKT | 4.02 | 8.51 | 67876.9 | TKT |
| sense | sp\|Q7KZF4\|SND1 | 4.01 | 5.49 | 101996.1 | SND1 TDRD11 |
| sense | sp\|Q71UI9\|H2AV;sp\|P0C0S5\|H2AZ | 4 | 42.97 | 13552.6 | H2AZ2 H2AFV H2AV |
| sense | sp\|Q71DI3\|H32 | 4 | 29.41 | 15387.9 | H3C15 HIST2H3A; H3C14 H3F2 H3FM HIST2H3C; H3C13 HIST2H3D |
| sense | sp\|P58107\|EPIPL | 4 | 4.93 | 555615.9 | EPPK1 EPIPL |
| sense | sp\|Q00839\|HNRPU | 4 | 2.55 | 90584.1 | HNRNPU C1orf199 HNRPU SAFA U21.1 |
| sense | sp\|P52272\|HNRPM | 4 | 6.71 | 77515.3 | HNRNPM HNRPM NAGR1 |
| sense | sp\|O75369\|FLNB | 4 | 1.69 | 278162.4 | FLNB FLN1L FLN3 TABP TAP |
| sense | sp\|Q13501\|SQSTM | 4 | 15.45 | 47686.7 | SQSTM1 ORCA OSIL |
| sense | sp\|P62081\|RS7 | 4 | 11.86 | 22126.7 | RPS7 |
| sense | sp\|P30050\|RL12 | 4 | 14.55 | 17818.4 | RPL12 |
| sense | sp\|P09923\|PPBI | 4 | 9.28 | 56811.7 | ALPI |
| sense | sp\|O00571\|DDX3X | 4 | 5.44 | 73242.8 | DDX3X DBX DDX3 |
| sense | sp\|O00231\|PSD11 | 4 | 14.69 | 47463.2 | PSMD11 |
| sense | sp\|Q9HCC0\|MCCB | 4 | 1.95 | 61332.7 | MCCC2 MCCB |
| sense | sp\|Q99714\|HCD2 | 4 | 13.03 | 26922.9 | HSD17B10 ERAB HADH2 MRPP2 SCHAD SDR5C1 XH98G2 |
| sense | sp\|Q96EP5\|DAZP1 | 4 | 10.32 | 43383.3 | DAZAP1 |
| sense | sp\|Q16658\|FSCN1 | 4 | 9.13 | 54529.5 | FSCN1 FAN1 HSN SNL |
| sense | sp\|Q13011\|ECH1 | 4 | 14.63 | 35815.8 | ECH1 |
| sense | sp\|Q08211\|DHX9 | 4 | 2.83 | 140957.5 | DHX9 DDX9 LKP NDH2 |
| sense | sp\|P63244\|RACK1 | 4 | 17.03 | 35076.5 | RACK1 GNB2L1 HLC7 PIG21 |
| sense | sp\|P63241\|IF5A1 | 4 | 42.21 | 16832.1 | EIF5A |
| sense | sp\|P62826\|RAN | 4 | 11.11 | 24423 | RAN ARA24 OK/SW-cl.81 |
| sense | sp\|P62753\|RS6 | 4 | 6.83 | 28680.4 | RPS6 OK/SW-cl.2 |
| sense | sp\|P61247\|RS3A | 4 | 7.20 | 29944.8 | RPS3A FTE1 MFTL |
| sense | sp\|P54578\|UBP14 | 4 | 6.88 | 56068.7 | USP14 TGT |
| sense | sp\|P48643\|TCPE | 4 | 8.69 | 59670.5 | CCT5 CCTE KIAA0098 |
| sense | sp\|P39748\|FEN1 | 4 | 4.74 | 42592.6 | FEN1 RAD2 |
| sense | sp\|P21796\|VDAC1 | 4 | 14.49 | 30772.4 | VDAC1 VDAC |
| sense | sp\|P12277\|KCRB | 4 | 7.61 | 42643.9 | CKB CKBB |
| sense | sp\|P10599\|THIO | 4 | 45.71 | 11737.4 | TXN TRDX TRX TRX1 |
| sense | sp\|P08865\|RSSA | 4 | 7.12 | 32853.8 | RPSA LAMBR LAMR1 |
| sense | sp\|P04792\|HSPB1 | 4 | 12.68 | 22782.3 | HSPB1 HSP27 HSP28 |
| sense | sp\|P12004\|PCNA | 3.96 | 23.75 | 28768.5 | PCNA |
| sense | sp\|P63010\|AP2B1 | 3.49 | 2.24 | 104551.6 | AP2B1 ADTB2 CLAPB1 |
| sense | sp\|P25705\|ATPA | 3.21 | 10.49 | 59750.1 | ATP5F1A ATP5A ATP5A1 ATP5AL2 ATPM |
| sense | sp\|P46782\|RS5 | 2.46 | 11.76 | 22876.2 | RPS5 |
| sense | sp\|Q05682\|CALD1 | 2.39 | 2.27 | 93230.5 | CALD1 CAD CDM |
| sense | sp\|P01876\|IGHA1 | 2.22 | 5.95 | 37654.3 | IGHA1 |
| sense | sp\|P61981\|1433G | 2.13 | 12.55 | 28302.3 | YWHAG |
| sense | sp\|P12956\|XRCC6 | 2.06 | 4.27 | 69842.4 | XRCC6 G22P1 |
| sense | sp\|O60506\|HNRPQ | 2.03 | 5.62 | 69602.3 | SYNCRIP HNRPQ NSAP1 |
| sense | sp\|P08195\|4F2 | 2.02 | 2.06 | 67993.3 | SLC3A2 MDU1 |
| sense | sp\|Q15149\|PLEC | 2.01 | 0.47 | 531785.9 | PLEC PLEC1 |
| sense | sp\|Q99832\|TCPH | 2.01 | 4.05 | 59366.1 | CCT7 CCTH NIP7-1 |
| sense | sp\|P62258\|1433E | 2.01 | 4.31 | 29173.6 | YWHAE |
| sense | sp\|P14649\|MYL6B | 2.01 | 6.25 | 22763.9 | MYL6B MLC1SA |
| sense | sp\|P0DOX5\|IGG1;sp\|P01857\|IGHG1 | 2.01 | 2.67 | 36105.7 | IGG1 IGHG1 |
| sense | sp\|P60709\|ACTB | 2 | 89.33 | 41736.4 | ACTB |
| sense | sp\|Q6FI13\|H2A2A;sp\|Q16777\|H2A2C | 2 | 57.69 | 13988.3 | H2AC18 H2AFO HIST2H2AA HIST2H2AA3; H2AC19 HIST2H2AA4 |
| sense | sp\|Q9BQE3\|TBA1C | 2 | 34.52 | 49894.9 | TUBA1C TUBA6 |
| sense | sp\|P0DMV9\|HS71B;sp\|P0DMV8\|HS71A | 2 | 8.11 | 70051.6 | HSPA1B HSP72 |
| sense | sp\|P22626\|ROA2 | 2 | 12.46 | 37429.7 | HNRNPA2B1 HNRPA2B1 |
| sense | sp\|P84243\|H33 | 2 | 28.68 | 15327.7 | H3-3A H3.3A H3F3 H3F3A PP781; H3-3B H3.3B H3F3B |
| sense | sp\|P15311\|EZRI | 2 | 2.73 | 69412.3 | EZR VIL2 |
| sense | sp\|Q92616\|GCN1 | 2 | 0.79 | 292755 | GCN1 GCN1L1 KIAA0219 |
| sense | sp\|P06753\|TPM3 | 2 | 4.56 | 32949.6 | TPM3 |
| sense | sp\|Q99623\|PHB2 | 2 | 6.69 | 33296.1 | PHB2 BAP REA |
| sense | sp\|Q14204\|DYHC1 | 2 | 0.39 | 532403.4 | DYNC1H1 DHC1 DNCH1 DNCL DNECL DYHC KIAA0325 |
| sense | sp\|P43243\|MATR3 | 2 | 2.95 | 94622.4 | MATR3 KIAA0723 |
| sense | sp\|P40925\|MDHC | 2 | 7.49 | 36425.8 | MDH1 MDHA |
| sense | sp\|P35579\|MYH9 | 2 | 0.56 | 226530.5 | MYH9 |
| sense | sp\|P28066\|PSA5 | 2 | 8.30 | 26410.8 | PSMA5 |
| sense | sp\|P22234\|PUR6 | 2 | 3.53 | 47078.8 | PAICS ADE2 AIRC PAIS |
| sense | sp\|O95782\|AP2A1 | 2 | 1.13 | 107544.7 | AP2A1 ADTAA CLAPA1 |
| sense | sp\|Q9BUJ2\|HNRL1 | 2 | 0.93 | 95738 | HNRNPUL1 E1BAP5 HNRPUL1 |
| sense | sp\|Q99497\|PARK7 | 2 | 10.05 | 19890.9 | PARK7 |
| sense | sp\|Q96RQ3\|MCCA | 2 | 1.52 | 80472.4 | MCCC1 MCCA |
| sense | sp\|Q92945\|FUBP2 | 2 | 1.83 | 73115.2 | KHSRP FUBP2 |
| sense | sp\|Q14566\|MCM6 | 2 | 1.22 | 92888.4 | MCM6 |
| sense | sp\|Q10713\|MPPA | 2 | 3.24 | 58252.4 | PMPCA INPP5E KIAA0123 MPPA |
| sense | sp\|P55072\|TERA | 2 | 3.60 | 89320.9 | VCP HEL-220 HEL-S-70 |
| sense | sp\|P50991\|TCPD | 2 | 6.31 | 57923.6 | CCT4 CCTD SRB |
| sense | sp\|P50990\|TCPQ | 2 | 3.10 | 59620.1 | CCT8 C21orf112 CCTQ KIAA0002 |
| sense | sp\|P47914\|RL29 | 2 | 9.43 | 17751.9 | RPL29 |
| sense | sp\|P46940\|IQGA1 | 2 | 1.15 | 189250.4 | IQGAP1 KIAA0051 |
| sense | sp\|P45974\|UBP5 | 2 | 2.80 | 95785.4 | USP5 ISOT |
| sense | sp\|P42704\|LPPRC | 2 | 1.36 | 157903.4 | LRPPRC LRP130 |
| sense | sp\|P35237\|SPB6 | 2 | 4.52 | 42621.6 | SERPINB6 PI6 PTI |
| sense | sp\|P23528\|COF1 | 2 | 16.87 | 18502.3 | CFL1 CFL |
| sense | sp\|P13489\|RINI | 2 | 2.17 | 49972.7 | RNH1 PRI RNH |
| sense | sp\|P10696\|PPBN;sp\|P05187\|PPB1 | 2 | 3.57 | 57953.3 | ALPG ALPPL ALPPL2 |
| sense | sp\|P09874\|PARP1 | 2 | 1.78 | 113082.9 | PARP1 ADPRT PPOL |
| sense | sp\|P09622\|DLDH | 2 | 3.73 | 54176.9 | DLD GCSL LAD PHE3 |
| sense | sp\|P07951\|TPM2 | 2 | 3.87 | 32850.4 | TPM2 TMSB |
| sense | sp\|P06744\|G6PI | 2 | 2.69 | 63146.7 | GPI |
| sense | sp\|P05198\|IF2A | 2 | 3.81 | 36111.8 | EIF2S1 EIF2A |
| sense | sp\|P05023\|AT1A1 | 2 | 1.66 | 112895 | ATP1A1 |
| sense | sp\|P00390\|GSHR | 2 | 4.41 | 56256.6 | GSR GLUR GRD1 |
| sense | sp\|O94979\|SC31A | 2 | 1.48 | 133013.6 | SEC31A KIAA0905 SEC31L1 HSPC275 HSPC334 |
| sense | sp\|O75534\|CSDE1 | 2 | 2.88 | 88884.1 | CSDE1 D1S155E KIAA0885 NRU UNR |
| sense | sp\|O75367\|H2AY | 2 | 10.48 | 39616.7 | MACROH2A1 H2AFY |
| sense | sp\|O43390\|HNRPR | 2 | 3.48 | 70942.8 | HNRNPR HNRPR |
| sense | sp\|Q9Y5S9\|RBM8A | 2 | 18.39 | 19888.9 | RBM8A RBM8 HSPC114 MDS014 |
| sense | sp\|Q9Y285\|SYFA | 2 | 2.76 | 57563.2 | FARSA FARS FARSL FARSLA |
| sense | sp\|Q9UN86\|G3BP2 | 2 | 5.81 | 54120.9 | G3BP2 KIAA0660 |
| sense | sp\|Q9NR45\|SIAS | 2 | 7.80 | 40307.3 | NANS SAS |
| sense | sp\|Q99729\|ROAA | 2 | 6.32 | 36224.8 | HNRNPAB ABBP1 HNRPAB |
| sense | sp\|Q96EY1\|DNJA3 | 2 | 4.79 | 52488.3 | DNAJA3 HCA57 TID1 |
| sense | sp\|Q96B49\|TOM6 | 2 | 22.97 | 8001.9 | TOMM6 OBTP TOM6 |
| sense | sp\|Q96AE4\|FUBP1 | 2 | 1.55 | 67560.2 | FUBP1 |
| sense | sp\|Q92597\|NDRG1 | 2 | 12.69 | 42835.2 | NDRG1 CAP43 DRG1 RTP |
| sense | sp\|Q8TDL5\|BPIB1 | 2 | 1.86 | 52440.9 | BPIFB1 C20orf114 LPLUNC1 UNQ706/PRO1357 |
| sense | sp\|Q8NHW5\|RLA0L;sp\|P05388\|RLA0 | 2 | 4.10 | 34273.2 | RPLP0P6 |
| sense | sp\|Q86V81\|THOC4 | 2 | 10.51 | 26887.8 | ALYREF ALY BEF THOC4 |
| sense | sp\|Q16881\|TRXR1 | 2 | 3.08 | 70905.6 | TXNRD1 GRIM12 KDRF |
| sense | sp\|Q16181\|SEPT7;sp\|Q15019\|SEPT2 | 2 | 3.66 | 41487.2 | SEPTIN7 CDC10 SEPT7 |
| sense | sp\|Q15084\|PDIA6 | 2 | 3.18 | 48120.9 | PDIA6 ERP5 P5 TXNDC7 |
| sense | sp\|Q14974\|IMB1 | 2 | 4.79 | 97169.2 | KPNB1 NTF97 |
| sense | sp\|Q13185\|CBX3;sp\|P83916\|CBX1 | 2 | 8.74 | 20811.2 | CBX3 |
| sense | sp\|Q13057\|COASY | 2 | 3.37 | 62328.2 | COASY PSEC0106 |
| sense | sp\|Q02978\|M2OM | 2 | 7.01 | 34061.4 | SLC25A11 SLC20A4 |
| sense | sp\|Q01650\|LAT1 | 2 | 3.55 | 55009.6 | SLC7A5 CD98LC LAT1 MPE16 |
| sense | sp\|P80723\|BASP1 | 2 | 10.57 | 22693.2 | BASP1 NAP22 |
| **sense** | **sp\|P67809\|YBOX1** | **2** | **11.11** | **35924.1** | **YBX1 NSEP1 YB1** |
| sense | sp\|P63096\|GNAI1;sp\|P08754\|GNAI3;sp\|P04899\|GNAI2 | 2 | 4.24 | 40531.8 | GNAI1 |
| sense | sp\|P62879\|GBB2;sp\|P62873\|GBB1 | 2 | 6.76 | 37376.6 | GNB2 |
| sense | sp\|P62854\|RS26 | 2 | 13.04 | 13015.3 | RPS26 |
| sense | sp\|P62266\|RS23 | 2 | 19.58 | 15807.5 | RPS23 |
| sense | sp\|P62244\|RS15A | 2 | 15.38 | 14839.4 | RPS15A OK/SW-cl.82 |
| sense | sp\|P62191\|PRS4 | 2 | 5.68 | 49184.1 | PSMC1 |
| sense | sp\|P62140\|PP1B | 2 | 3.06 | 37186.5 | PPP1CB |
| sense | sp\|P61513\|RL37A | 2 | 19.57 | 10275.2 | RPL37A |
| sense | sp\|P61313\|RL15 | 2 | 12.75 | 24145.9 | RPL15 EC45 TCBAP0781 |
| sense | sp\|P60900\|PSA6 | 2 | 7.32 | 27399.2 | PSMA6 PROS27 |
| sense | sp\|P52907\|CAZA1 | 2 | 5.25 | 32922.5 | CAPZA1 |
| sense | sp\|P49368\|TCPG | 2 | 3.67 | 60533.3 | CCT3 CCTG TRIC5 |
| sense | sp\|P42765\|THIM | 2 | 8.56 | 41923.8 | ACAA2 |
| sense | sp\|P37802\|TAGL2 | 2 | 9.04 | 22391.4 | TAGLN2 KIAA0120 CDABP0035 |
| sense | sp\|P35998\|PRS7 | 2 | 3.69 | 48633.4 | PSMC2 MSS1 |
| sense | sp\|P32969\|RL9 | 2 | 21.35 | 21863.3 | RPL9 OK/SW-cl.103; RPL9P7; RPL9P8; RPL9P9 |
| sense | sp\|P31949\|S10AB | 2 | 30.48 | 11740.3 | S100A11 MLN70 S100C |
| sense | sp\|P31689\|DNJA1 | 2 | 7.56 | 44868 | DNAJA1 DNAJ2 HDJ2 HSJ2 HSPF4 |
| sense | sp\|P30685\|1B35;sp\|P30491\|1B53;sp\|P30484\|1B46;sp\|P30466\|1B18;sp\|P30464\|1B15;sp\|P18465\|1B57;sp\|P10319\|1B58 | 2 | 9.39 | 40336.8 | HLA-B HLAB |
| sense | sp\|P26038\|MOES | 2 | 2.77 | 67819.6 | MSN |
| sense | sp\|P25787\|PSA2 | 2 | 8.97 | 25898.4 | PSMA2 HC3 PSC3 |
| sense | sp\|P25311\|ZA2G | 2 | 3.36 | 34258.5 | AZGP1 ZAG ZNGP1 |
| sense | sp\|P25205\|MCM3 | 2 | 2.47 | 90979.9 | MCM3 |
| sense | sp\|P23396\|RS3 | 2 | 7.41 | 26688.1 | RPS3 OK/SW-cl.26 |
| sense | sp\|P20042\|IF2B | 2 | 7.51 | 38388.1 | EIF2S2 EIF2B |
| sense | sp\|P18669\|PGAM1 | 2 | 10.63 | 28803.7 | PGAM1 PGAMA CDABP0006 |
| sense | sp\|P14406\|CX7A2 | 2 | 28.92 | 9395.9 | COX7A2 COX7AL |
| sense | sp\|P13010\|XRCC5 | 2 | 4.24 | 82703.8 | XRCC5 G22P2 |
| sense | sp\|P0DP25\|CALM3;sp\|P0DP24\|CALM2;sp\|P0DP23\|CALM1 | 2 | 14.77 | 16837.5 | CALM3 CALML2 CAM3 CAMC CAMIII |
| sense | sp\|P07910\|HNRPC | 2 | 5.23 | 33669.7 | HNRNPC HNRPC |
| sense | sp\|P05186\|PPBT | 2 | 3.63 | 57304.4 | ALPL |
| sense | sp\|P01591\|IGJ | 2 | 7.55 | 18098.4 | JCHAIN IGCJ IGJ |
| sense | sp\|O95881\|TXD12 | 2 | 22.67 | 19205.6 | TXNDC12 TLP19 UNQ713/PRO1376 |
| sense | sp\|O75964\|ATP5L | 2 | 24.27 | 11428.4 | ATP5MG ATP5L |
| sense | sp\|O75822\|EIF3J | 2 | 5.04 | 29062.2 | EIF3J EIF3S1 PRO0391 |
| sense | sp\|O60884\|DNJA2 | 2 | 3.64 | 45745.4 | DNAJA2 CPR3 HIRIP4 |
| sense | sp\|O43169\|CYB5B | 2 | 23.29 | 16332 | CYB5B CYB5M OMB5 |
| sense | sp\|O00410\|IPO5 | 2 | 1.73 | 123628.9 | IPO5 KPNB3 RANBP5 |
| sense | sp\|O00159\|MYO1C | 2 | 0.85 | 121680.6 | MYO1C |
| sense | sp\|Q5T749\|KPRP | 1.64 | 1.73 | 64135.2 | KPRP C1orf45 |
| sense | sp\|P41252\|SYIC | 1.42 | 1.74 | 144496.9 | IARS1 IARS |
| sense | sp\|Q01518\|CAP1 | 1.39 | 3.79 | 51901.1 | CAP1 CAP |
| sense | sp\|P31327\|CPSM | 1.36 | 1.00 | 164938.1 | CPS1 |
| antisense | sp\|P60709\|ACTB | 44.55 | 68.00 | 41736.4 | ACTB |
| antisense | sp\|P08238\|HS90B | 26.58 | 30.52 | 83263.5 | HSP90AB1 HSP90B HSPC2 HSPC3 HSPCB |
| antisense | sp\|P04406\|G3P | 24.2 | 56.42 | 36053 | GAPDH GAPD CDABP0047 OK/SW-cl.12 |
| antisense | sp\|P06733\|ENOA | 19.45 | 41.24 | 47168.6 | ENO1 ENO1L1 MBPB1 MPB1 |
| antisense | sp\|P11021\|GRP78 | 16 | 17.43 | 72332.4 | HSPA5 GRP78 |
| antisense | sp\|P78371\|TCPB | 14.89 | 20.00 | 57487.6 | CCT2 99D8.1 CCTB |
| antisense | sp\|P00338\|LDHA | 14.74 | 40.06 | 36688.5 | LDHA PIG19 |
| antisense | sp\|P14625\|ENPL | 13.51 | 12.33 | 92468.1 | HSP90B1 GRP94 HSPC4 TRA1 |
| antisense | sp\|P13639\|EF2 | 12.47 | 13.99 | 95337.4 | EEF2 EF2 |
| antisense | sp\|P10809\|CH60 | 10.93 | 20.77 | 61054.2 | HSPD1 HSP60 |
| antisense | sp\|P38646\|GRP75 | 10.02 | 13.70 | 73680 | HSPA9 GRP75 HSPA9B mt-HSP70 |
| antisense | sp\|P11498\|PYC | 10.01 | 5.60 | 129632.6 | PC |
| antisense | sp\|P68363\|TBA1B | 10 | 20.40 | 50151.2 | TUBA1B |
| antisense | sp\|Q5VTE0\|EF1A3;sp\|P68104\|EF1A1 | 9.4 | 20.78 | 50140.6 | EEF1A1P5 EEF1AL3 |
| antisense | sp\|Q9UHB6\|LIMA1 | 8.65 | 6.46 | 85224.9 | LIMA1 EPLIN SREBP3 PP624 |
| antisense | sp\|P07437\|TBB5 | 8.04 | 18.92 | 49670.5 | TUBB TUBB5 OK/SW-cl.56 |
| antisense | sp\|P14618\|KPYM | 8.03 | 14.50 | 57936.4 | PKM OIP3 PK2 PK3 PKM2 |
| antisense | sp\|P06576\|ATPB | 8 | 17.58 | 56559.4 | ATP5F1B ATP5B ATPMB ATPSB |
| antisense | sp\|P04075\|ALDOA | 7.47 | 25.27 | 39419.7 | ALDOA ALDA |
| antisense | sp\|Q9BTM1\|H2AJ;sp\|Q99878\|H2A1J;sp\|Q96KK5\|H2A1H;sp\|P20671\|H2A1D;sp\|P0C0S8\|H2A1 | 7.18 | 58.14 | 13936.2 | H2AJ H2AFJ |
| antisense | sp\|P60174\|TPIS | 7.12 | 22.73 | 30790.8 | TPI1 TPI |
| antisense | sp\|P06748\|NPM | 6.61 | 19.05 | 32574.8 | NPM1 NPM |
| antisense | sp\|P11142\|HSP7C | 6.26 | 16.25 | 70897.6 | HSPA8 HSC70 HSP73 HSPA10 |
| antisense | sp\|P07355\|ANXA2 | 6.19 | 8.85 | 38603.6 | ANXA2 ANX2 ANX2L4 CAL1H LPC2D |
| antisense | sp\|P07900\|HS90A | 6.03 | 24.73 | 84659 | HSP90AA1 HSP90A HSPC1 HSPCA |
| antisense | sp\|P29401\|TKT | 6 | 11.24 | 67876.9 | TKT |
| antisense | sp\|Q9UQ80\|PA2G4 | 5.96 | 15.23 | 43786.6 | PA2G4 EBP1 |
| antisense | sp\|P08865\|RSSA | 5.89 | 10.85 | 32853.8 | RPSA LAMBR LAMR1 |
| antisense | sp\|P05783\|K1C18 | 5.12 | 11.86 | 48057.4 | KRT18 CYK18 PIG46 |
| antisense | sp\|Q05682\|CALD1 | 4.33 | 3.78 | 93230.5 | CALD1 CAD CDM |
| antisense | sp\|P07195\|LDHB | 4.03 | 14.37 | 36638.2 | LDHB |
| antisense | sp\|P61978\|HNRPK | 4.02 | 3.67 | 50975.8 | HNRNPK HNRPK |
| antisense | sp\|Q96EP5\|DAZP1 | 4.02 | 10.32 | 43383.3 | DAZAP1 |
| antisense | sp\|Q06830\|PRDX1 | 4.02 | 14.57 | 22110.2 | PRDX1 PAGA PAGB TDPX2 |
| antisense | sp\|P53396\|ACLY | 4.01 | 3.91 | 120838.3 | ACLY |
| antisense | sp\|Q15366\|PCBP2 | 4.01 | 8.77 | 38579.7 | PCBP2 |
| antisense | sp\|Q71UI9\|H2AV;sp\|P0C0S5\|H2AZ | 4 | 42.97 | 13552.6 | H2AZ2 H2AFV H2AV |
| antisense | sp\|P08670\|VIME | 4 | 4.72 | 53651.2 | VIM |
| antisense | sp\|Q71DI3\|H32 | 4 | 24.26 | 15387.9 | H3C15 HIST2H3A; H3C14 H3F2 H3FM HIST2H3C; H3C13 HIST2H3D |
| antisense | sp\|P14923\|PLAK | 4 | 3.22 | 81744 | JUP CTNNG DP3 |
| antisense | sp\|Q13835\|PKP1 | 4 | 3.48 | 82859.8 | PKP1 |
| antisense | sp\|Q00839\|HNRPU | 4 | 2.55 | 90584.1 | HNRNPU C1orf199 HNRPU SAFA U21.1 |
| antisense | sp\|P68431\|H31 | 4 | 24.26 | 15403.9 | H3C1 H3FA HIST1H3A; H3C2 H3FL HIST1H3B; H3C3 H3FC HIST1H3C; H3C4 H3FB HIST1H3D; H3C6 H3FD HIST1H3E; H3C7 H3FI HIST1H3F; H3C8 H3FH HIST1H3G; H3C10 H3FK HIST1H3H; H3C11 H3FF HIST1H3I; H3C12 H3FJ HIST1H3J |
| antisense | sp\|P19338\|NUCL | 4 | 2.54 | 76613.9 | NCL |
| antisense | sp\|P07814\|SYEP | 4 | 2.25 | 170589.7 | EPRS1 EPRS GLNS PARS QARS QPRS PIG32 |
| antisense | sp\|Q9HCC0\|MCCB | 4 | 3.91 | 61332.7 | MCCC2 MCCB |
| antisense | sp\|P80723\|BASP1 | 4 | 22.91 | 22693.2 | BASP1 NAP22 |
| antisense | sp\|P63241\|IF5A1 | 4 | 34.42 | 16832.1 | EIF5A |
| antisense | sp\|P62258\|1433E | 4 | 12.16 | 29173.6 | YWHAE |
| antisense | sp\|P55809\|SCOT1 | 4 | 6.15 | 56157.2 | OXCT1 OXCT SCOT |
| antisense | sp\|P17987\|TCPA | 4 | 8.99 | 60342.9 | TCP1 CCT1 CCTA |
| antisense | sp\|P08195\|4F2 | 4 | 4.76 | 67993.3 | SLC3A2 MDU1 |
| antisense | sp\|P07737\|PROF1 | 4 | 13.57 | 15054.1 | PFN1 |
| antisense | sp\|P04792\|HSPB1 | 4 | 12.68 | 22782.3 | HSPB1 HSP27 HSP28 |
| antisense | sp\|P23528\|COF1 | 3.77 | 23.49 | 18502.3 | CFL1 CFL |
| antisense | sp\|P10696\|PPBN;sp\|P05187\|PPB1 | 3.38 | 8.46 | 57953.3 | ALPG ALPPL ALPPL2 |
| antisense | sp\|P63104\|1433Z | 3.32 | 17.55 | 27744.8 | YWHAZ |
| antisense | sp\|Q99880\|H2B1L;sp\|Q99879\|H2B1M;sp\|Q99877\|H2B1N;sp\|Q93079\|H2B1H;sp\|Q8N257\|H2B3B;sp\|Q5QNW6\|H2B2F;sp\|Q16778\|H2B2E;sp\|P62807\|H2B1C;sp\|P58876\|H2B1D;sp\|P57053\|H2BFS;sp\|P33778\|H2B1B;sp\|P23527\|H2B1O;sp\|P06899\|H2B1J;sp\|O60814\|H2B1K | 3.29 | 19.05 | 13890 | H2BC13 H2BFC HIST1H2BL |
| antisense | sp\|P11586\|C1TC | 2.96 | 7.49 | 101558.4 | MTHFD1 MTHFC MTHFD |
| antisense | sp\|P15924\|DESP | 2.61 | 0.63 | 331771.2 | DSP |
| antisense | sp\|P81605\|DCD | 2.61 | 32.73 | 11283.7 | DCD AIDD DSEP |
| antisense | sp\|P48643\|TCPE | 2.58 | 3.51 | 59670.5 | CCT5 CCTE KIAA0098 |
| antisense | sp\|P26599\|PTBP1 | 2.19 | 9.04 | 57220.9 | PTBP1 PTB |
| antisense | sp\|Q92688\|AN32B | 2.07 | 17.93 | 28787.4 | ANP32B APRIL PHAPI2 |
| antisense | sp\|P63261\|ACTG | 2.06 | 68.00 | 41792.5 | ACTG1 ACTG |
| antisense | sp\|P49327\|FAS | 2.04 | 2.39 | 273424.1 | FASN FAS |
| antisense | sp\|P12956\|XRCC6 | 2.03 | 4.27 | 69842.4 | XRCC6 G22P1 |
| antisense | sp\|P84243\|H33 | 2.02 | 23.53 | 15327.7 | H3-3A H3.3A H3F3 H3F3A PP781; H3-3B H3.3B H3F3B |
| antisense | sp\|P60900\|PSA6 | 2.02 | 7.32 | 27399.2 | PSMA6 PROS27 |
| antisense | sp\|P55084\|ECHB | 2.02 | 8.86 | 51294 | HADHB MSTP029 |
| antisense | sp\|P30101\|PDIA3 | 2.02 | 5.15 | 56781.8 | PDIA3 ERP57 ERP60 GRP58 |
| antisense | sp\|Q14204\|DYHC1 | 2.01 | 0.39 | 532403.4 | DYNC1H1 DHC1 DNCH1 DNCL DNECL DYHC KIAA0325 |
| antisense | sp\|O43175\|SERA | 2.01 | 2.81 | 56650 | PHGDH PGDH3 |
| antisense | sp\|P55072\|TERA | 2.01 | 3.60 | 89320.9 | VCP HEL-220 HEL-S-70 |
| antisense | sp\|P35232\|PHB | 2.01 | 7.35 | 29803.8 | PHB1 PHB |
| antisense | sp\|Q6FI13\|H2A2A;sp\|Q16777\|H2A2C | 2 | 57.69 | 13988.3 | H2AC18 H2AFO HIST2H2AA HIST2H2AA3; H2AC19 HIST2H2AA4 |
| antisense | sp\|P0DMV9\|HS71B;sp\|P0DMV8\|HS71A | 2 | 8.11 | 70051.6 | HSPA1B HSP72 |
| antisense | sp\|Q15365\|PCBP1 | 2 | 8.99 | 37497.5 | PCBP1 |
| antisense | sp\|P09923\|PPBI | 2 | 8.52 | 56811.7 | ALPI |
| antisense | sp\|Q99832\|TCPH | 2 | 4.60 | 59366.1 | CCT7 CCTH NIP7-1 |
| antisense | sp\|P58107\|EPIPL | 2 | 3.06 | 555615.9 | EPPK1 EPIPL |
| antisense | sp\|O43390\|HNRPR | 2 | 3.48 | 70942.8 | HNRNPR HNRPR |
| antisense | sp\|Q86VP6\|CAND1 | 2 | 2.20 | 136374.1 | CAND1 KIAA0829 TIP120 TIP120A |
| antisense | sp\|P15880\|RS2 | 2 | 3.75 | 31324.2 | RPS2 RPS4 |
| antisense | sp\|P06753\|TPM3 | 2 | 4.56 | 32949.6 | TPM3 |
| antisense | sp\|Q96RQ3\|MCCA | 2 | 1.52 | 80472.4 | MCCC1 MCCA |
| antisense | sp\|Q86V81\|THOC4 | 2 | 10.51 | 26887.8 | ALYREF ALY BEF THOC4 |
| antisense | sp\|Q01844\|EWS | 2 | 2.13 | 68478.2 | EWSR1 EWS |
| antisense | sp\|Q01650\|LAT1 | 2 | 3.55 | 55009.6 | SLC7A5 CD98LC LAT1 MPE16 |
| antisense | sp\|Q00610\|CLH1 | 2 | 0.66 | 191613 | CLTC CLH17 CLTCL2 KIAA0034 |
| antisense | sp\|P62879\|GBB2 | 2 | 6.76 | 37330.6 | GNB2 |
| antisense | sp\|P62266\|RS23 | 2 | 19.58 | 15807.5 | RPS23 |
| antisense | sp\|P60842\|IF4A1 | 2 | 4.19 | 46153.5 | EIF4A1 DDX2A EIF4A |
| antisense | sp\|P49748\|ACADV | 2 | 2.59 | 70389.6 | ACADVL VLCAD |
| antisense | sp\|P26038\|MOES | 2 | 1.73 | 67819.6 | MSN |
| antisense | sp\|O95831\|AIFM1 | 2 | 1.79 | 66900.1 | AIFM1 AIF PDCD8 |
| antisense | sp\|O00159\|MYO1C | 2 | 0.85 | 121680.6 | MYO1C |
| antisense | sp\|Q9BYE4\|SPR2G;sp\|P35326\|SPR2A;sp\|P35325\|SPR2B;sp\|P22532\|SPR2D;sp\|P22531\|SPR2E | 2 | 17.81 | 7905.3 | SPRR2G |
| antisense | sp\|Q99733\|NP1L4 | 2 | 7.20 | 42823.1 | NAP1L4 NAP2 |
| antisense | sp\|Q99623\|PHB2 | 2 | 6.69 | 33296.1 | PHB2 BAP REA |
| antisense | sp\|Q92598\|HS105 | 2 | 2.56 | 96864.3 | HSPH1 HSP105 HSP110 KIAA0201 |
| antisense | sp\|Q8NC51\|PAIRB | 2 | 5.15 | 44965.2 | SERBP1 PAIRBP1 CGI-55 |
| antisense | sp\|Q16836\|HCDH | 2 | 11.78 | 34293.3 | HADH HAD HAD1 HADHSC SCHAD |
| antisense | sp\|Q15631\|TSN | 2 | 9.65 | 26182.7 | TSN |
| antisense | sp\|Q15517\|CDSN | 2 | 3.40 | 51521.6 | CDSN |
| antisense | sp\|Q13011\|ECH1 | 2 | 6.71 | 35815.8 | ECH1 |
| antisense | sp\|Q12874\|SF3A3 | 2 | 4.39 | 58848.4 | SF3A3 SAP61 |
| antisense | sp\|Q04941\|PLP2 | 2 | 8.55 | 16690.5 | PLP2 A4 |
| antisense | sp\|Q01813\|PFKAP | 2 | 3.32 | 85595.4 | PFKP PFKF |
| antisense | sp\|Q01518\|CAP1 | 2 | 3.79 | 51901.1 | CAP1 CAP |
| antisense | sp\|P63244\|RACK1 | 2 | 3.16 | 35076.5 | RACK1 GNB2L1 HLC7 PIG21 |
| antisense | sp\|P63096\|GNAI1;sp\|P08754\|GNAI3;sp\|P04899\|GNAI2 | 2 | 4.24 | 40531.8 | GNAI1 |
| antisense | sp\|P62826\|RAN | 2 | 8.80 | 24423 | RAN ARA24 OK/SW-cl.81 |
| antisense | sp\|P62753\|RS6 | 2 | 6.83 | 28680.4 | RPS6 OK/SW-cl.2 |
| antisense | sp\|P62191\|PRS4 | 2 | 5.68 | 49184.1 | PSMC1 |
| antisense | sp\|P61247\|RS3A | 2 | 6.44 | 29944.8 | RPS3A FTE1 MFTL |
| antisense | sp\|P52907\|CAZA1 | 2 | 5.25 | 32922.5 | CAPZA1 |
| antisense | sp\|P52272\|HNRPM | 2 | 4.52 | 77515.3 | HNRNPM HNRPM NAGR1 |
| antisense | sp\|P47914\|RL29 | 2 | 9.43 | 17751.9 | RPL29 |
| antisense | sp\|P40925\|MDHC | 2 | 7.49 | 36425.8 | MDH1 MDHA |
| antisense | sp\|P39687\|AN32A | 2 | 13.65 | 28585.1 | ANP32A C15orf1 LANP MAPM PHAP1 |
| antisense | sp\|P31947\|1433S | 2 | 10.48 | 27773.8 | SFN HME1 |
| antisense | sp\|P31930\|QCR1 | 2 | 2.50 | 52645.3 | UQCRC1 |
| antisense | sp\|P30050\|RL12 | 2 | 9.09 | 17818.4 | RPL12 |
| antisense | sp\|P27708\|PYR1 | 2 | 1.98 | 242981.7 | CAD |
| antisense | sp\|P27348\|1433T | 2 | 5.71 | 27763.9 | YWHAQ |
| antisense | sp\|P26641\|EF1G | 2 | 7.78 | 50118.4 | EEF1G EF1G PRO1608 |
| antisense | sp\|P25787\|PSA2 | 2 | 8.97 | 25898.4 | PSMA2 HC3 PSC3 |
| antisense | sp\|P25311\|ZA2G | 2 | 3.36 | 34258.5 | AZGP1 ZAG ZNGP1 |
| antisense | sp\|P24752\|THIL | 2 | 6.32 | 45199.2 | ACAT1 ACAT MAT |
| antisense | sp\|P22626\|ROA2 | 2 | 7.65 | 37429.7 | HNRNPA2B1 HNRPA2B1 |
| antisense | sp\|P21796\|VDAC1 | 2 | 7.77 | 30772.4 | VDAC1 VDAC |
| antisense | sp\|P20042\|IF2B | 2 | 7.51 | 38388.1 | EIF2S2 EIF2B |
| antisense | sp\|P14406\|CX7A2 | 2 | 28.92 | 9395.9 | COX7A2 COX7AL |
| antisense | sp\|P13489\|RINI | 2 | 2.17 | 49972.7 | RNH1 PRI RNH |
| antisense | sp\|P12277\|KCRB | 2 | 10.24 | 42643.9 | CKB CKBB |
| antisense | sp\|P12004\|PCNA | 2 | 8.81 | 28768.5 | PCNA |
| antisense | sp\|P10599\|THIO | 2 | 26.67 | 11737.4 | TXN TRDX TRX TRX1 |
| antisense | sp\|P09622\|DLDH | 2 | 3.73 | 54176.9 | DLD GCSL LAD PHE3 |
| antisense | sp\|P06744\|G6PI | 2 | 2.69 | 63146.7 | GPI |
| antisense | sp\|P02545\|LMNA | 2 | 1.51 | 74138.8 | LMNA LMN1 |
| antisense | sp\|O00422\|SAP18 | 1.8 | 12.42 | 17561 | SAP18 GIG38 |
| antisense | sp\|P61981\|1433G | 1.75 | 5.67 | 28302.3 | YWHAG |
| antisense | sp\|Q92945\|FUBP2 | 1.62 | 1.83 | 73115.2 | KHSRP FUBP2 |
| antisense | sp\|Q02978\|M2OM | 1.54 | 7.01 | 34061.4 | SLC25A11 SLC20A4 |
| antisense | sp\|Q8IX12\|CCAR1 | 1.51 | 1.74 | 132820 | CCAR1 CARP1 DIS |
| antisense | sp\|P25705\|ATPA | 1.39 | 6.51 | 59750.1 | ATP5F1A ATP5A ATP5A1 ATP5AL2 ATPM |

**Supplementary Figures**

Fig. S1 Transcriptome-based WGCNA analysis identified hub lncRNA CARMN related to the NOR-CIN-CC transition

(a). The FPKM distribution of samples. The plot showed the density distribution of the log10-transformed FPKM values across different sample groups, normal (NOR), CIN, and tumor samples (CC). (b). The violin plot of sample expression. The plot illustrated the expression levels of individual samples using a violin plot, where log10-transformed FPKM values are plotted for each sample group, providing a comparison of expression variability across normal, CIN, and tumor categories. (c). The PCA results demonstrated the differences and dispersion among the samples. (d) Sample dendrogram and trait heatmap displaying clustering and trait association in gene expression data. (e) Scale independence and mean connectivity plots for soft thresholding power selection in WGCNA (β = 9). (f) The eigengene adjacency heatmap of the 34 modules. (g) Network heatmap plot of selected genes (top 800) showing co-expression modules. (h) The heatmap of gene expression from Blue and Lightyellow modules. (i-j) GO enrichment analysis of hub mRNAs in the Blue (i) and Lightyellow (j) Module. (k) The constructed mRNA-lncRNA networks in the Blue and Lightyellow modules. Squares and circles represent lncRNAs and mRNAs, respectively. The solid line represents a positive correlation, while the dashed line represents a negative correlation. (l) The constructed mRNA-TF-lncRNA networks in the two modules. TFs were predicted by PROMO website.

Fig. S2 Normalization and batch effect correction of the CC dataset

(a) Gene distribution in datasets before and after normalization. (b) Gene distribution before and after batch effect correction. (c) The PCA results showed that the sample characteristics varied significantly across datasets before batch correction.

Fig. S3 Construction of CC diagnostic models and identification of key lncRNAs using machine learning algorithms

(a-j) Diagnostic models for CC were constructed using ten different machine learning algorithms: Decision Tree (DT), Random Forest (RF), Extreme Gradient Boosting (XGBoost), Elastic Net Regression (ENR), Support Vector Machine (SVM), Multi-Layer Perceptron (MLP), Light Gradient Boosting Machine (LightGBM), k-Nearest Neighbors (KNN), Logistic Regression (LR), and Stacking Ensemble (Stacking) For all models except XGBOOST, KNN, and LR, the following components are presented in five parts each: ROC curves of the model; variable importance interpretation based on DALEX; variable importance based on SHAP values; summary of variable importance based on SHAP values, with results ranked for each group; and a stacked bar plot of variable importance based on SHAP values. For models XGBOOST, KNN, and LR, the summary of variable importance based on SHAP values is already shown in Fig. 1.

Fig. S4 CARMN expression in TCGA database. CESC, Cervical Squamous Cell Carcinoma and Endocervical Adenocarcinoma; READ, Rectum Adenocarcinoma; BLCA, Bladder Urothelial Carcinoma; UCEC, Uterine Corpus Endometrial Carcinoma; BRCA, Breast Invasive Carcinoma; ESCA, Esophageal Carcinoma; LUSC, Lung Squamous Cell Carcinoma; STAD, Stomach Adenocarcinoma; KICH, Kidney Chromophobe; PRAD, Prostate Adenocarcinoma; HNSC, Head and Neck squamous cell carcinoma; COAD, Colon Adenocarcinoma; LUAD, Lung Adenocarcinoma; PAAD, Pancreatic Adenocarcinoma; THCA, Thyroid Carcinoma; PCPG, Pheochromocytoma and Paraganglioma; LIHC, Liver Hepatocellular Carcinoma; CHOL, Cholangiocarcinoma; KIRP, Kidney Renal Papillary Cell Carcinoma.

Fig. S5 CARMN overexpression inhibited the malignant phenotype of CC cells

(a) The expression levels of CARMN in the CARMN stably high-expressed C33A cells. (b) The effect of CARMN overexpression on the proliferation of C33A cells by CCK-8. (c) The effect of CARMN overexpression on the migration and invasion abilities of C33A cells by transwell assays. (d) The effect of CARMN overexpression on the cell cycles of C33A cells by flow cytometry. (e-f) The effect of CARMN overexpression on the expression levels of cell cycle-related proteins in HeLa (e) and C33A (f) cells by WB. (g) The effect of CARMN overexpression on the apoptosis of C33A cells by flow cytometry. ns, *P* ≥ 0.05; *, *P* < 0.05; **, *P* < 0.01; ***, *P* < 0.001.

Fig. S6 CARMN inhibition of the malignant phenotype in CC cells was independent of miR-143

(a) The expression levels of CARMN in the CARMN-ASO C33A cells. (b) The effect of CARMN knockdown (at a concentration of 50 nM ASO) on the proliferation of C33A cells by CCK-8. (c-d) The effect of CARMN-ASO (at a concentration of 50 nM) on the cell cycles (c) and apoptosis (d) of C33A cells by flow cytometry. (e) The volume of xenografts. (f) The expression of miR-143-3p and miR-145-5p from the RNA-seq of HeLa-CARMN and -NC cells, respectively. (g) The expression of CARMN after miR-143-3p inhibitor treatment in HeLa cells by qPCR. (h) The effect of miR-143-3p inhibitor on cell cycle by flow cytometry in the HeLa-CARMN cells, respectively. ns, *P* ≥ 0.05; *, *P* < 0.05; **, *P* < 0.01; ***, *P* < 0.001.

Fig. S7 CARMN inhibited the Akt-mTOR signaling pathway in CC cells.

(a) The expression of p-Akt (Thr308), p-mTOR (Ser2448) and their total proteins in C33A cells with or without CARMN overexpression. (b) Exploring the optimal treatment concentration of SC79 through the level of AKT activation. (c) The effect of SC79 on the cell cycle in the C33A-CARMN cells. (d-e) The expression of cell cycle-related proteins after SC79 treatment in HeLa (d) and C33A (e) cells with CARMN overexpression. (f) The effect of SC79 on the colony formation ability of C33A -CARMN cells by plate colony formation assay. ns, *P* ≥ 0.05; *, *P* < 0.05; **, *P* < 0.01; ***, *P* < 0.001.

Fig. S8 CARMN inhibited autophagic flux by suppressing the Akt/mTOR pathway

(a) The expression levels of LC3 and p62 mRNA and protein in C33A cells with or without CARMN overexpression. (b-c) The levels of LC3B and p62 proteins in the HeLa cells treated with different concentrations of CQ (b, 10μM) or Baf-A1 (c, 100 nM). (d-e) Detection of the effects of Baf-A1 (d) or CQ (e) treatment on autophagy levels in CARMN-overexpressing cells by WB. (f) The cell cycles of the C33A-CARMN cells treated with CQ or Baf-A1. (g-h) The colony formation ability (g) and proliferation (h) of C33A-CARMN cells treated with CQ or Baf-A1. (i) The expression of LC3B and p62 proteins after SC79 treatment in C33A-CARMN cells. ns, *P* ≥ 0.05; *, *P* < 0.05; **, *P* < 0.01; ***, *P* < 0.001.

Fig. S9 CARMN inhibits Akt/mTOR signaling pathway activation by upregulating ROS levels in cells

(a) ROS levels using DHE probe with flow cytometry in C33A cells with or without CARMN overexpression. (b) Detection of the Relative Quantity of ROS-Positive Cells by Flow Cytometry. (c) The effect of NAC on the Akt and mTOR expression in C33A cells with CARMN overexpression. (d) Cell cycles in the C33A-CARMN cells treated with NAC. (e) WB revealing expression of cell cycle-related markers in the C33A-CARMN cells treated with NAC. (f-g) The proliferation of HeLa-CARMN (f) and C33A-CARMN (g) treated with NAC by CCK-8. ns, *P* ≥ 0.05; *, *P* < 0.05; **, *P* < 0.01; ***, *P* < 0.001.

Fig. S10 CARMN promoted ferroptosis-independent ROS levels by inhibiting the nuclear translocation of Nrf2

(a) qPCR analysis of ferroptosis-related genes in the HeLa-CARMN and -NC cells. (b) WB detecting GPX4 expression. (c) The effect of TBHQ on the protein levels of Nrf2 in the HeLa-CARMN cells. (d) The effect of TBHQ on the target genes of Nrf2 in the HeLa-CARMN cells. (e) The effect of TBHQ on the protein levels of Nrf2 and its targets in the HeLa-CARMN cells. (f) The effect of CARMN on the immunoprecipitated Keap1 and ubiquitinated Nrf2 by co-IP. ns, *P* ≥ 0.05; *, *P* < 0.05; **, *P* < 0.01; ***, *P* < 0.001.

Fig. S11 CARMN inhibited MAPK13-mediated MAPK cascade

(a) The expression of MAPK cascade in the C33A cells with or without CARMN overexpression. (b) CCK-8 assay results showed that honokiol treatment enhanced the proliferation ability of CARMN-overexpressing C33A cells. (c) The expression of eight dysregulated genes related to MAPK signaling pathway in the C33A-CARMN and -NC cells validated by qPCR. (d) qPCR detection of the effect of knocking down miR-143-3p on MAPK13 expression in HeLa CAMRN and C33A-CARMN (e) MAPK13 expression in the HeLa and C33A cells treated with or without MAPK13 by qPCR and WB. (f) The effect of MAPK13 on the expression of branches of MAPK cascade in the C33A-CARMN and -NC cells. (g) The proliferation of C33A-CARMN cells with or without MAPK13 overexpression by CCK8. (h) The migration abilities of HeLa- and C33A-CARMN cells with or without MAPK13 overexpression. ns, *P* ≥ 0.05; *, *P* < 0.05; **, *P* < 0.01; ***, *P* < 0.001.

Fig. S12 CARMN inhibited the MAPK cascade by suppressing the transcriptional activation of MAPK13

(a) The effect of TFAP2α overexpression plasmid and ASO on the protein expression of MAPK13 in the CC cells. (b) The effect of TFAP2α overexpression plasmid (left) and ASO (right) on the mRNA expression of MAPK13 in the CC cells detected by qPCR. (c) The effect of TFAP2α overexpression plasmid and ASO on the expression of CARMN in the CC cells detected by qPCR. (d) Luciferase reporter assay detecting the influence of TFAP2α on the transcriptional activity of MAPK13 promoter in SiHa cells. (e) Luciferase reporter plasmids containing varied length of MAPK13 promoter regions were constructed to determine the potential binding sites of TFAP2α in SiHa cell. (f) qPCR detecting TFAP2α mRNA expression in CC cells with or without CARMN overexpression. (g) ChIP assay detecting the levels of immunoprecipitated chromatin by anti-TFAP2α and control IgG in SiHa cell. (h) RIP assay revealed the directly binding of CARMN to TFAP2α in C33A cell. (i) The directly binding of CARMN to TFAP2α via its sense strand by RNA-pulldown assay in HeLa cell. ns, *P* ≥ 0.05; *, *P* < 0.05; **, *P* < 0.01; ***, *P* < 0.001.

Fig. S13 Transcriptional dysregulation of CARMN in CC

(a) Luciferase activities of reporter plasmids containing varied CARMN promoters in SiHa cell. (b-c) The effect of SP1-siR NA on the expression of SP1 protein (b) and mRNA (c) in CC cells. (d) Luciferase reporter assay detecting the effect of SP1 on the transcriptional activity of CARMN using SP1-siRNA in SiHa cell. (e) The expression of CARMN and miR-143/145 in SiHa cell treated with or without SP1-siRNA. (f) Luciferase reporter assay detecting the effect of SNP rs12517403 T to C transition on the transcriptional activity of CARMN in SiHa cell. (g) The effect of SNP rs12517403 in combination of SP1 on the transcriptional activity of CARMN in SiHa cell. (h) The effect of rs12517403 polymorphism on CARMN expression in SiHa cell. ns, *P* ≥ 0.05; *, *P* < 0.05; **, *P* < 0.01; ***, *P* < 0.001.

Fig. S14 Post-transcriptional dysregulation of CARMN in CC

(a) qPCR detecting the expression of components of CRD after ASO treatment in HeLa cell. (b) Expression of CARMN in cells after RBP knockdown by ASO in SiHa cell. ns, *P* ≥ 0.05; *, *P* < 0.05; **, *P* < 0.01; ***, *P* < 0.001.
